# Supplementary material for: Exploring online sensor parameters as proxies for polar organic chemicals—An innovative approach for combined sewer overflow monitoring
Source: PLoS One. 2025 Oct 13;20(10):e0333173. doi: 10.1371/journal.pone.0333173 (PMC12517529; doi:10.1371/journal.pone.0333173)
Supplement: S1 File — This file provides additional information on statistical analyses, methodological details, and supporting data visualization. (PDF) [file pone.0333173.s001.pdf]

S1_File: Supporting information

Exploring online sensor parameters as proxies for polar organic chemicals – An innovative approach for combined sewer overflow monitoring

Laura Waldner[1,2]*, Viviane Furrer[1], Pierre Lechevallier[1,2], Fabienne Maire[1,2], Heinz Singer[1], Lena Mutzner[1]*

[1] Department of Urban Water Management, Swiss Federal Institute of Aquatic Science and Technology (Eawag), 8600 Dübendorf, Switzerland
[2] Institute of Environmental Engineering (IfU), ETH Zürich, 8093 Zurich, Switzerland
*Corresponding authors: laura.waldner@eawag.ch, lena.mutzner@eawag.ch

Table of contents

1. Sampling 1
2. Analysis of organic chemicals 4
3. Online sensors 8
4. Cost estimates for organic chemical monitoring 11
5. Details on data analysis 12
6. Analysis of dilution time lag of organic chemicals from municipal wastewater 13
7. Hierarchical clustering of organic chemicals from catchments of different sizes 16
8. Correlations between organic chemicals of one chemical group 19
9. Temporal dynamics of sensor parameters 21
10. Correlations between organic chemicals and sensor parameters 24
11. Correlations between organic chemicals and UV-Vis absorbance in catchment M 31
12. Predicting organic chemical concentrations from sensor parameters 33
13. Random forest regression models for catchment L 43

References 45

1. Sampling

S1 Table 1 shows rain duration, rain intensity, and preceding dry periods estimated based on precipitation measurements. The sewer flow rate during the sampling time was measured with a flowmeter (S1 Table 7). The preceding dry period was calculated as the time between two rain events (rain > 1 mm/h for > 10 minutes in catchments S and L, rain > 0.1 mm for > 10 minutes in catchment M).

S1 Table 1. Characteristics of rain events and flow rate during the sampling period. This table shows the sampling time, rain duration and volume, preceding dry period, and flow rate during the sampling time. This study defined a rain event period during which the CSO is active (catchments S and L) or the flow exceeds its dry weather baseline (catchment M). Rain and flow data are available from two openly accessible datasets in the Eawag Research Data Institutional Collection [1] (catchment M) and [2] (catchments S and L).

	Sampling time	Rain duration [hh:mm]	Rain [mm] (max. intensity [mm/h])	Preceding dry period [dd:hh]	Sewer flow rate [m^3/h]
**Catchment S**					
Rain event S.1	19:21-20:44 10.09.2021	00:13	3.2 (25.8)	41:00	40-2231 (avg. 345)
Rain event S.2	18:48-20:01 16.09.2021	02:05	19.6 (45.0)	00:04	1391-2970 (avg. 2273)
Rain event S.3	08:48-10:16 02.11.2021	01:58	2.8 (6.6)	00:05	92-925 (avg. 283)
Rain event S.4	16:15-19:04 01.12.2021	02:23	1.9 (9.6)	00:13	226-676 (avg. 311)
Rain event S.5	06:12-10:01 04.12.2021	14:32	24.6 (7.8)	14:00	295-981 (avg. 470)
Rain event S.6	08:01-09:41 08.12.2021	25:24	30.6 (66.6)	00:19	277-835 (avg.355)
Rain event S.7	18:57-19:35 30.03.2022	00:35	2.5 (18.6)	00:05	186-1209 (avg. 485)
Rain event S.8	09:24-11:04 01.04.2022	22:09	28.2 (9.0)	00:05	241-991 (avg. 335)
Rain event S.9	18:40-22:38 24.04.2022	11:30	28.1 (16.2)	00:13	238-979 (avg. 483)
Rain event S.10	08:49-12:09 26.04.2022	02:36	9.0 (12.6)	00:06	218-1368 (avg. 605)
Rain event S.11	17:06-19:26 05.05.2022	07:54	53.2 (37.2)	01:00	1467-7986 (avg. 4613)
Rain event S.12	09:11-12:41 06.05.2022	04:16	4.4 (7.2)	00:02	514-990 (avg. 768)
Rain event S.13	16:40-18:00 23.05.2022	00:42	6.1 (29.4)	00:12	172-2265 (avg. 655)
Rain event S.14	06:11-07:21 24.05.2022	00:45	1.6 (5.4)	00:06	133-816 (avg. 250)
Rain event S.15	16:25-17:15 01.06.2022	00:09	0.4 (16.2)	00:01	118-200 (avg. 143)
Rain event S.16	06:54-08:24 04.06.2022	00:51	2.5 (10.2)	00:15	91-993 (avg. 223)
Rain event S.17	13:27-15:27 05.06.2022	01:04	6.1 (35.4)	01:06	79-2215 (avg. 590)
Rain event S.18	21:17-00:57 05/06.06.2022	00:57	5.4 (74.4)	00:05	105-274 (avg. 203)
Rain event S.19	04:22-05:42 16.06.2022	00:22	0.8 (42.6)	02:23	177-4011 (avg. 915)
**Catchment M**					

Rain event M.1	09:37-13:00<br>21.06.2023	00:20	9.4<br>(50.4)	00:12	365-2447<br>(avg. 1091)
Rain event M.2	08:44-11:14<br>14.10.2023	02:30	3.5<br>(3.8)	21:17	321-840<br>(avg. 538)
Rain event M.3	13:00-16:40<br>20.10.2023	06:10	3.1<br>(1.4)	05:20	368-703<br>(avg. 588)
Rain event M.4	10:00-14:30<br>24.10.2023	03:40	5.8<br>(4.7)	02:14	300-1577<br>(avg. 843)
**Catchment L**					
Rain event L.1	04:08-16:58<br>19.08.2022	11:53	77.2<br>(54.0)	00:06	4831-11583<br>(avg. 8824)
Rain event L.2	15:30-16:04<br>20.08.2022	00:27	12.5<br>(65.0)	00:01	5429-7696<br>(avg.6606)
Rain event L.3	20:02-21:03<br>20.08.2022	01:04	15.2<br>(48.0)	00:04	4777-8491<br>(avg. 7489)
Rain event L.4	12:04-14:16<br>31.08.2022	03:13	18.8<br>(19.0)	00:03	5333-9011<br>(avg. 7712)
Rain event L.5	21:13-21:53<br>07.09.2022	01:05	10.0<br>(19.0)	00:13	5288-7992<br>(avg. 7086)
Rain event L.6	00:55-07:16<br>08.09.2022	7:16	47.5<br>(38.0)	00:02	5279-9966<br>(avg. 8770)
Rain event L.7	20:25-21:39<br>15.09.2022	02:21	14.5<br>(14.0)	00:02	5454-8930<br>(avg. 7658)
Rain event L.8	01:46-03:49<br>16.09.2022	02:08	22.0<br>(48.0)	00:01	5288-10736<br>(avg. 9426)
Rain event L.9	09:33-15:16<br>28.09.2022	18.39	619.5<br>(49.0)	00:07	4847-11898<br>(avg. 9289)
Rain event L.10	09:00-10:19<br>29.09.2022	04:06	6.4<br>(4.0)	00:00	4765-8128<br>(avg. 7166)
Rain event L.11	16:30-00:10<br>09./10.05.2023	09:11	31.8<br>(34.0)	00:17	2638-9302<br>(avg. 7221)
Rain event L.12	17:20-21:40<br>22.06.2023	00:41	4.2<br>(13.0)	01:23	1750-5173<br>(avg. 3301)
Rain event L.13	05:36-07:00<br>23.06.2023	03:10	12.2<br>(12.0)	00:02	5307-8862<br>(avg. 7640)

S1 Table 2. Sampling times, sample types, and number of samples taken per rain event. Organic chemical data are available from two openly accessible datasets in the Eawag Research Data Institutional Collection [1] (catchment M) and [2] (catchments S and L).

	Sampling time	Sample type	Number of samples
**Catchment S**			
Rain event S.1	19:21-20:44 10.09.2021	Grab	11
Rain event S.2	18:48-20:01 16.09.2021	Grab	7
Rain event S.3	08:48-10:16 02.11.2021	Grab	10
Rain event S.4	16:15-19:04 01.12.2021	Grab	18
Rain event S.5	06:12-10:01 04.12.2021	Grab	24
Rain event S.6	08:01-09:41 08.12.2021	Grab	11
Rain event S.7	18:57-19:35 30.03.2022	Grab	13
Rain event S.8	09:24-11:04 01.04.2022	Grab	17
Rain event S.9	18:40-22:38 24.04.2022	Grab, composite	22, 17
Rain event S.10	08:49-12:09 26.04.2022	Grab, composite	21, 16
Rain event S.11	17:06-19:26 05.05.2022	Composite	15
Rain event S.12	09:11-12:41 06.05.2022	Composite	21
Rain event S.13	16:40-18:00 23.05.2022	Composite	9
Rain event S.14	06:11-07:21 24.05.2022	Composite	8
Rain event S.15	16:25-17:15 01.06.2022	Composite	6
Rain event S.16	06:54-08:24 04.06.2022	Composite	10
Rain event S.17	13:27-15:27 05.06.2022	Composite	11
Rain event S.18	21:17 05.06.2022 - 00:57 06.06.2022	Composite	23
Rain event S.19	04:22-05:42 16.06.2022	Composite	9
**Catchment M**			
Rain event M.1	09:37-13:00 21.06.2023	Grab	21
Rain event M.2	08:44-11:14 14.10.2023	Grab	16
Rain event M.3	13:00-16:40 20.10.2023	Composite	23
Rain event M.4	10:00-14:30 24.10.2023	Composite	26
**Catchment L**			
Rain event L.1	04:08-16:58 19.08.2022	Grab, composite	17, 66
Rain event L.2	15:30-16:04 20.08.2022	Composite	4
Rain event L.3	20:02-21:03 20.08.2022	Composite	7
Rain event L.4	12:04-14:16 31.08.2022	Composite	14
Rain event L.5	21:13-21:53 07.09.2022	Composite	5
Rain event L.6	00:55-07:16 08.09.2022	Composite	39
Rain event L.7	20:25-21:39 15.09.2022	Composite	9
Rain event L.8	01:46-03:49 16.09.2022	Composite	10
Rain event L.9	09:33-15:16 28.09.2022	Composite	19
Rain event L.10	09:00-10:19 29.09.2022	Composite	9
Rain event L.11	17:30 09.05.2023 - 00:10 10.05.2023	Composite	41
Rain event L.12	18:20-21:40 22.06.2023	Composite	21
Rain event L.13	05:36-07:00 23.06.2023	Composite	9

2. Analysis of organic chemicals

S1 Table 3. Measured organic chemicals, their use, and $LogK_{ow}$. $LogK_{ow}$ values were obtained from the Royal Society of Chemistry [3] and the National Library of Medicine [4]. Abbreviations: personal care product (PCP), plant protection product (PPP).

Chemical group	Organic chemical (abbreviation)	InChIKey	Source/use	$LogK_{ow}$
Municipal wastewater: in-door	Acesulfame (ACE)	YGCFIWIQZPHFLU-UHFFFAOYSA-N	Food additive (sweetener)	-1.33
	Caffeine (CAF)	RYYVLZVUVIJVGH-UHFFFAOYSA-N	Food additive (coffee & tea)	-0.07
	Cyclamate (CYC)	HCAJEUSONLESMK-UHFFFAOYSA-N	Food additive (sweetener)	-1.61
	Candesartan (CAN)	HTQMVQVXFRQIKW-UHFFFAOYSA-N	Pharmaceutical (antihyperten-sive drug)	4.79
	Citalopram (CIT)	WSEQXVZVJXJVFP-UHFFFAOYSA-N	Pharmaceutical (antidepressant)	1.39
	Diclofenac (DCF)	DCOPUUMXTXDBNB-UHFFFAOYSA-N	Pharmaceutical (non-steroidal anti-inflammatory drug)	4.51
	Hydrochlorothiazide (HCT)	JZUFKLXOESDKRF-UHFFFAOYSA-N	Pharmaceutical (antihyperten-sive drug)	-0.07
	Triclosan (TCS)	XEFQLINVKFYRCS-UHFFFAOYSA-N	Biocide in PCPs (anti-microbial agent)	4.76
Stormwater: road	1,3-diphenylguanidine (DPG)	OWRCNXZUPFZXOS-UHFFFAOYSA-N	Tire wear (vulcanization accel-erator)	2.89
	6PPD-quinone (6PPDQ)	UBMGKRIXKUIXFQ-UHFFFAOYSA-N	Tire wear (ozone protection and antioxidant in tires)	4.47
	Hexa(methoxyme-thyl)melamine (HMMM)	BNCADMBVWNPPIZ-UHFFFAOYSA-N	Tire wear (cross-linking agent in coatings and tires)	-0.05
Stormwater: bio-cides & PPPs	2,4-dichlorophenoxya-cetic acid (2,4-D)	OVSKIKFHRZPJSS-UHFFFAOYSA-N	PPP	2.81
	Carbendazim (CBZ)	TWFZGCMQGLPBSX-UHFFFAOYSA-N	Biocide (facades) & legacy PPP	1.52
	Diuron (DCMU)	XMTQQYYKAHVGBJ-UHFFFAOYSA-N	Biocide (facades) & legacy PPP	2.68
	2-methyl-4-chlorophe-noxyacetic acid (MCPA)	WHKUVVPPKQRRBV-UHFFFAOYSA-N	PPP	3.25
	Mecoprop-p (MPP)	WNTGYJSOUMFZEP-UHFFFAOYSA-N	Biocide (flat roofs) & PPP	3.13
	2-n-octyl-4-isothia-zolin-3-on (OIT)	JPMIIZHYYWMHDT-UHFFFAOYSA-N	Biocide (facades)	2.45
Municipal wastewater & stormwater: di-verse	1H-benzotriazole (BT)	CMGDVUCDZOBDNL-UHFFFAOYSA-N (4-MBT), LRUDIIUSNGCQKF-UHFFFAOYSA-N (5-MBT)	Household (dishwash detergent) & industry (corrosion inhibitor, UV-stabilizer)	1.71
	4-&5-methylbenzotria-zole (MeBT)	QRUDEWIWKLJBPS-UHFFFAOYSA-N	Household (dishwash detergent) & industry (corrosion inhibitor, UV-stabilizer)	1.44
	N,N-diethyl-meta-to-luamide (DEET)	MMOXZBCLCQITDF-UHFFFAOYSA-N	PCP (insect repellent sprays) & biocide	2.02

S1 Table 4. List of limits of quantification (LOQ) and isotope-labelled standards for identification of target analytes. For catchment M, two LOQ values are given as samples were analyzed in two runs. The first number applies to samples taken during rain event M.1 and M.4, and the second number to samples taken during rain events M.2 and M.3. The information displayed in this table is obtained from Furrer et al. [5,6] and Lechevallier et al. [7].

Group	Sub-stance	Catchments S & L		Catchment M	
		**LOQ [ng/L]**	**Isotope-labelled standard**	**LOQ [ng/L]**	**Isotope-labelled standard**
Indoor	ACE	50	Acesulfame-D4	25, 10	Acesulfame-D4
	CAF	100	Caffeine-D9	50, 50	Benzotriazole-D4
	CYC	50	Cyclamate-D11	25, 25	Hydrochlorothiazide-13C,D2
	CAN	10	Candesartan-D5	5, 25	Candesartan-D5
	CIT	5	Citalopram-D6	5, 25	Venlafaxine-D6
	DCF	10	Diclofenac-D4	5, 25	Diclofenac-D4
	HCT	25	Hydrochlorothiazide-C13,D2	5, 25	Hydrochlorothiazide-13C,D2
	TCS	10	Triclosan-D3	50, 75	Mecoprop-p-D6
Road	DPG	10	Lidocain-D10	10, 25	Benzotriazole-D4
	6PPDQ	10	6PPD-quinone-D5	10, 25	6PPD-quinone-D5
	HMMM	25	Terbutryn-D5	2.5, 5	DEET-D10
PPPs & bio-cides	2,4-D	5	2,4-D-D3	25, 50	2,4-D-D3
	CBZ*	5	Carbendazim-D4	5, 10	Carbendazim-D4
	DCMU	10	Diuron-D6	25, 10	Diuron-D6
	MCPA	10	MCPA-D3	25, 25	MCPA-D3
	MPP	10	Mecoprop-p-D6	25, 10	Mecoprop-p-D6
	OIT	5	Octhilinon-D17	2.5, 100	Metolachlor-D6
Diverse	BT	100	Benzotrizaole-D4	250, 250	Benzotriazole-D4
	MeBT	25	4-&5-methylbenzotriazole-D6	25, 10	Venlafaxine-D6
	DEET	10	DEET-D10	100, 75	DEET-D10

*CBZ was only measured in catchments S and M, due to problems with the isotope-labelled internal standard when measuring samples taken in catchment L.

S1 Table 5. Relative recovery of spiked samples from catchments S and L. To quantify the relative recovery, four samples were spiked with a known concentration (S1 and S2 with 250 ng/L; S3 and S4 with 2,500 ng/L). The relative recovery was calculated as: Rel. recovery [%] = (conc_spiked_sample – conc_unspiked_sample) / conc_spiked * 100.“<LOQ” indicates that a chemical's concentration was below the limit of quantification (LOQ) in the sample. “n.a.” indicates that the relative recovery could not be calculated. The information displayed in this table is obtained from Furrer et al. [5,6].

		Concentration in samples [ng/L]				Rel. recovery [%]			
**Group**	**Substance**	**S1**	**S2**	**S3**	**S4**	**S1**	**S2**	**S3**	**S4**
Indoor	ACE	1390	3683	1793	3089	n.a.	n.a.	95	103
	CAF	4434	8211	4507	9658	n.a.	n.a.	142	124
	CYC	3258	4824	6730	13468	n.a.	n.a.	81	78
	CAN	37	17	59	33	105	100	n.a.	n.a.
	CIT	<LOQ	8	5	11	100	98	n.a.	n.a.
	DCF	343	3150	399	1458	n.a.	n.a.	102	99
	HCT	78	258	67	303	100	94	n.a.	n.a.
	TCS	<LOQ	33	<LOQ	27	96	100	n.a.	n.a.
Road	DPG	821	414	753	387	83	99	93	101
	6PPDQ	34	<LOQ	35	<LOQ	154	135	n.a.	n.a.
	HMMM	446	569	419	543	105	105	103	110
PPPs & biocides	2,4-D	<LOQ	11	9	11	106	99	n.a.	n.a.
	CBZ	<LOQ.	7	5	7	108	106	n.a.	n.a.
	DCMU	<LOQ	15	<LOQ	14	111	107	n.a.	n.a.
	MCPA	<LOQ	10	7	11	101	95	n.a.	n.a.
	MPP	123	182	138	185	106	110	n.a.	n.a.
	OIT	<LOQ	12	<LOQ	9	113	103	n.a.	n.a.
Diverse	BT	1250.	1338	1108	1658	n.a.	n.a.	108	93
	MeBT	608	403	546	382	169	105	107	105
	DEET	18	37	21	27	115	108	n.a.	n.a.

S1 Table 6. Relative recovery of spiked samples from catchment M. To quantify the relative recovery, eight samples were spiked with two known concentrations (500 ng/L and 2,500 ng/L). The relative recovery was calculated as: Rel. recovery [%] = (conc_spiked_sample – conc_unspiked_sample) / conc_spiked * 100. The average relative recovery of both spiked samples (500 ng/L and 2,500 ng/L) is shown here. When the relative recovery of one spike was 130% > x or < 70%, only the spike with relative recovery in the range 70-130% is shown. "<LOQ" indicates that a chemical's concentration was below the limit of quantification (LOQ) in the sample. "n.a." indicates that the relative recovery could not be calculated. The information displayed in this table is obtained from Lechevallier et al. [7].

Group	Substance	Concentration in samples [ng/L]								Rel. recovery [%]							
		S1	S2	S3	S4	S5	S6	S7	S8	S1	S2	S3	S4	S5	S6	S7	S8
Indoor	ACE	85010	27405	5859	12407	15708	33664	67838	52151	101	104	111	101	110	107	102	96
	CAF	205080	94466	5113	28101	42584	84285	117231	60758	104	115	108	120	132	135	114	106
	CYC	123094	58510	8955	29540	20965	54765	145589	88226	115	108	109	104	119	112	97	97
	CAN	933	435	220	425	433	573	1295	844	77	78	96	92	92	90	86	80
	CIT	161	45	<LOQ	115	69	65	108	53	97	98	120	121	117	110	99	97
	DCF	3116	1188	501	2016	1666	2217	5483	1499	122	106	149	223	184	176	116	120
	HCT	960	351	157	367	646	761	1254	519	99	102	116	118	123	109	98	98
	TCS	247	112	<LOQ	<LOQ	<LOQ	<LOQ	<LOQ	<LOQ	96	105	109	117	112	130	114	100
Road	DPG	195	2553	777	697	329	865	780	4660	97	85	210	162	100	93	74	63
	6PPDQ	<LOQ	96	40	<LOQ	<LOQ	<LOQ	<LOQ	89	101	111	112	105	95	95	107	107
	HMMM	226	502	233	193	126	549	5866	2346	105	107	113	99	99	100	200	117
PPPs & biocides	2,4-D	<LOQ	<LOQ	<LOQ	<LOQ	<LOQ	<LOQ	<LOQ	141	110	108	113	118	111	117	112	102
	CBZ	22	41	<LOQ	<LOQ	<LOQ	<LOQ	14	14	99	99	109	107	n.a.	202	115	105
	DCMU	61	<LOQ	129	181	19318	6266	630	891	121	115	116	115	119	114	116	122
	MCPA	<LOQ	75	<LOQ	<LOQ	<LOQ	<LOQ	<LOQ	<LOQ	104	108	106	105	104	100	106	107
	MPP	218	1241	55	380	70	117	<LOQ	158	94	92	120	131	117	115	94	92
	OIT	6	17	<LOQ	<LOQ	400	120	41	41	99	99	32	89	110	125	90	85
Diverse	BT	412	3296	473	604	1397	1977	4681	1069	87	77	185	145	264	142	173	111
	MeBT	2845	1274	636	6888	2573	2495	6950	2164	110	91	159	n.a.	254	178	n.a.	99
	DEET	4351	1156	608	477	1386	954	1225	667	168	122	156	129	168	126	121	104

3. Online sensors

S1 Table 7. Characteristics of the sensors used to measure wastewater characteristics. "-" indicates that sensor measurements are missing. The sensor information summarized in this table is obtained from Furrer et al. [5,6] and Lechevallier et al. [7].

	Sensor name (manufacturer)	Measurement interval	Location	Maintenance	Calibration
**Rain**					
Catchment S	Pluvo^{2}L (OTT HydroMet)	1 min	Weather station Russikon	Manual cleaning (2x yearly)	2x yearly weight accuracy calibration
Catchment M	Pluvio^{2}S (OTT HydroMet)	10 min	Weather station Eawag	Manual cleaning (every few weeks, if necessary)	Every 2 years
Catchment L	Pluvio^{2}L (OTT HydroMet)	1 min	Weather station Siehbach	Manual cleaning (every 3 months)	None
**Level**					
Catchment S	Micropilot FMR20 (Endress+Hauser)<br><br>EchoMax XRS-5 (Siemens)	10 s<br><br>1 min	Sewer pipe before overflow<br>Retention basin	Manual cleaning at installation (temporary measurement)<br>Manual cleaning (1x yearly)	1x after first rain event<br><br>1x yearly manual reference measurement
Catchment M	i3 (Nivus)	2 min	Sewer pipe	Manual cleaning (1-2x yearly)	1-2x yearly manual reference measurement
Catchment L	-	-	-	-	-
**Flow**					
Catchment S*	Flow CSO to river 2021: Flo-Dar (Hach company)<br><br>Flow CSO to river 2022: EchoMax XRS-5 (Siemens)<br><br>Flow CSO to WWTP: Flo-Dar (Hach company)	5 min<br><br>1 min<br><br>5 min	Overflow pipe (from CSO to river)<br><br>Overflow edge<br><br>Sewer pipe downstream of CSO	Manual cleaning at installation (temporary measurement)<br><br>n.a. (calculated from water level sensor)<br><br>Manual cleaning (2x yearly)	1x at installation<br><br>n.a. (calculated from water level sensor)<br><br>2x yearly
Catchment M	CS2 (Nivus)	2 min	Sewer pipe	Manual cleaning (1-2x yearly)	1-2x yearly
Catchment L	POA V2D and V2H (Nivus)	1 min	Sewer pipe	Manual cleaning (every 3 months)	None (regular comparison to water volumes in WWTP)
**Temperature**					
Catchment S	-	-	-	-	-
Catchment M	ISEmax CAS40D (Endress+Hauser)	2 min	Sewer bypass	Manual cleaning (every 1-2 wk)	None
Catchment L	1200-S sc V2 (Hach Lange)	1 min	Sewer bypass	Manual cleaning (1x weekly)	Every 3 months
**EC**					

Catchment S	2021: Memosens CLS82D (Endress+Hauser)<br><br>2022: Indumax CLS50 (Endress+Hauser)	1 s<br><br>10 s	Sewer by-pass<br><br>Sewer pipe before over-flow	Manual cleaning (1x weekly)	At installation (tempo-rary measurement)
Catchment M	E53<br>(GLI International)	2 min	Primary clar-ifier	None	None
Catchment L	3798-S sc V2<br>(Hach Lange)	1 min	Sewer by-pass	Manual cleaning (1x weekly)	Every 3 months
**pH**					
Catchment S	-	-	-	-	-
Catchment M	ISEmax CAS40D (Endress+Hauser)	2 min	Sewer by-pass	Manual cleaning (every 1-2 wk)	None
Catchment L	1200-S sc V2<br>(Hach Lange)	1 min	Sewer by-pass	Manual cleaning (1x weekly)	Every 3 months
**Turbidity**					
Catchment S	-	-	-	-	-
Catchment M	Turbimax CUS51D (Endress+Hauser)	2 min	Sewer by-pass	Automatic pressurized air cleaning (every 10 min), manual cleaning (every 1-2 wk)	At installation (no drift observed)
Catchment L	-	-	-	-	-
**$SAC_{254\ nm}$**					
Catchment S	-	-	-	-	-
Catchment M	ISA<br>(GO Sys-temelektronik)	2 min	Sewer by-pass	Automatic pressurized air cleaning (every 10 min), manual cleaning (every 1-2 wk)	At installation (no drift observed)
Catchment L	Spectro::lyser IN-FLUENTV160 (S::CAN)	1 min	Sewer by-pass	Manual cleaning (1x weekly)	None
**NH_4-N**					
Catchment S	Ion chromatog-raphy<br>930 Compact IC Flex,<br>(Methrom)<br><br>Photometry:<br>Lachat QC8500 (Hach Lange)<br><br>Dr. Lange:<br>LCK304/0<br>(Hach Lange)	10 min	Lab	n.a.	n.a.
Catchment M	Photometry:<br>Lachat QC8500 (Hach Lange)	10 min	Lab	n.a.	n.a.
Catchment L	Ion chromatog-raphy:<br>930 Compact IC Flex<br>(Methrom)<br><br>Photometry:	10 min	Lab	n.a.	n.a.

	Lachat QC8500 (Hach Lange)<br>Dr. Lange: LCK304/05 (Hach Lange)				

*Furrer et al. [2] calculated the total inflow to the CSO structure based on the CSO discharge to the river, the discharge to the WWTP, and the flow to fill the inflow channel and retention basin (based on water level and geometry of the inlet channel and the retention basin).

4. Cost estimates for organic chemical monitoring

S1 Tables 8-9 provide an overview of the estimated investment costs and operational effort associated with sensor-based monitoring and LC-MS-based monitoring of organic chemicals. The cost estimates are based on our expertise and the equipment used for organic chemical and wastewater monitoring by Furrer et al. [5,6] and Lechevallier et al. [7]. Comparing these tables indicates that sensor-based monitoring is generally more cost- and time-efficient than automated sampling followed by LC-MS analysis for long-term monitoring campaigns (see manuscript section 3.5 Practical considerations for future application of sensor-based monitoring and remaining research needs).

S1 Table 8. Estimated investment cost and operational effort for the sensor-based monitoring approach. The table displays approximate cost ranges, as exact costs depend strongly on the sensor type, quality, and data transmissions.

	**Equipment**	**Estimated investment cost [€]**	**Operational effort**
Sensors	Level	1,000-2,000	Calibration: approx. 30 min/sensor<br>Maintenance: approx. 15 min/sensor
	Flow	6,500-12,000	
	Temperature	<1,000	
	EC	1,500-2,500	
	pH (incl. temperature)	1,500-2,000	
	Turbidity	2,000-3,000	
	UV-Vis ($SAC_{254\ nm}$)	20,000-30,000	
	Ion selective electrode (NH_4-N, pH, temperature)	4,000-6,000	
Data handling	Data logger & transmitter	1,500-13,000	None
	Data interpretation	None	1 hour/week

S1 Table 9. Estimated investment cost and operational effort for automated water sampling followed by LC-MS analysis. The table displays approximate costs as exact costs vary by equipment type and quality. Investment costs are provided for in-house laboratory analysis (excluding staff costs) and for outsourcing to a contract laboratory.

	**Equipment**	**Estimated investment cost [€]**	**Operational effort**
Sampling	Automated sampler	5,000-13,000	Hours-days
In-house sample processing and measurement	Centrifuge	5,000	Hours
	LC-MS device	300,000-400,000 (for a Triple Quadrupole)	Days-weeks
	Software for data interpretation and validation	Included in costs for LC-MS device	A few weeks
Sample processing and measurement in a contract laboratory	None	1,000/sample	None

5. Details on data analysis

Pretreatment of sensor data. Sensor data was available at various resolutions (1-second, 1-minute, 2-minute, and 5-minute, S1 Table 7). Linear interpolation was used to convert 5-minute and 2-minute to 1-minute resolution data, where necessary.

Linear correlations. The PCC was calculated as follows: $PCC = \frac{\sum_{i=1}^{n}(x_i-\bar{x})(y_i-\bar{y})}{\sqrt{\sum_{i=1}^{n}(x_i-\bar{x})^2}\sqrt{\sum_{i=1}^{n}(y_i-\bar{y})^2}}$, where n: number of data points, x_i, y_i: chemical/parameter at the time point i, $\bar{x}$, $\bar{y}$: mean of chemical/parameter x, y.
The Z-score was calculated as follows: $z = \frac{x-\bar{x}}{\sigma}$, where σ: standard deviation of chemical/sensor parameter.

Linear regression models. The ordinary least squares linear regression function used (LinearRegression from the `sklearn` module `linear_model`) cannot handle missing values (concentrations < LOQ). Thus, when data was missing, either the sensor or the rain event was excluded from the regression model, depending on the size of the remaining dataset:

- Catchment S: rain events without NH_4-N measurements (S.7, S.8, S.10, S.13, and S.14) were excluded.
- Catchment M: chemicals and sensors unavailable for all four rain events (2,4-D, Carbendazim, MCPA, OIT, and EC) were excluded.
- Catchment L: Carbendazim data was excluded (problems with the isotope-labelled internal standard during measurements).

The absolute and relative root mean square error (RMSE and RRMSE) between measured and predicted concentrations was calculated to evaluate model predictions. RRMSEs were calculated to compare chemicals with different concentration ranges and to measure the relative error of prediction. The RMSE and RRMSE are easily interpretable measures of the standard deviation of the model prediction and its relative error. However, these measures could not be calculated when a chemical was not detected in the validation set and no reference measurement data were available. The R^2 of prediction was also calculated. The R^2 is maximal 1 for a perfect model prediction, but it can be strongly negative for a model performing worse than a horizontal line. Thus, models with $R^2 < -1$ have a disproportionately strong influence on the median R^2. Therefore, RMSE and RRMSE are considered better measures of prediction error than R^2.

6. Analysis of dilution time lag of organic chemicals from municipal wastewater

Delayed dilution of organic chemicals from municipal wastewater was observed during some rain events. In these cases, maximum flow values were reached before minimum concentrations were measured. This phenomenon was observed in all three catchments, as shown in S1 Fig. 1. However, the length of the time lag varied between rain events.

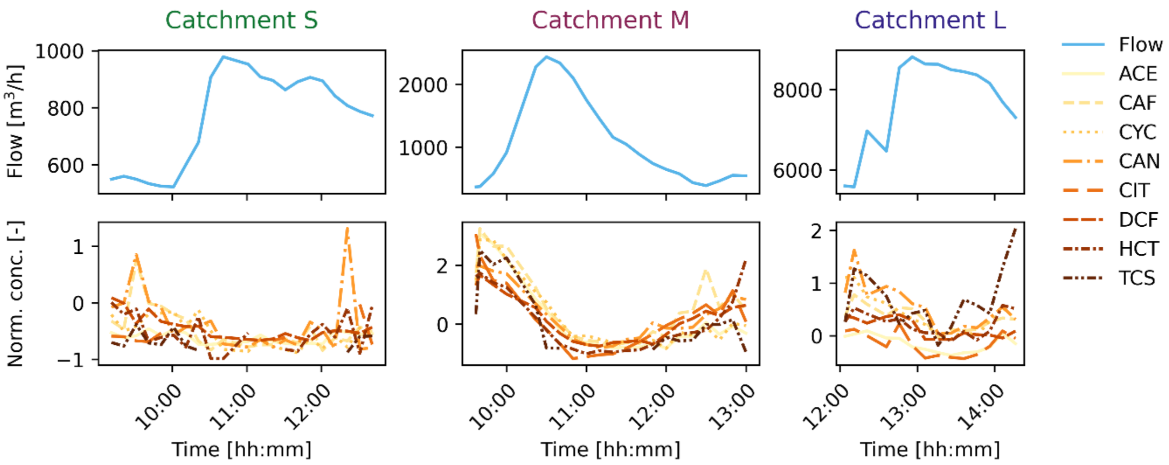

S1 Fig. 1. Examples of delayed dilution of organic chemicals from municipal wastewater. The figure shows the observed flow and concentration patterns for rain events S.12, M.1, and L.4.

Dilution of organic chemicals from wastewater by stormwater has been observed in other studies [8,9], but not with a time lag. The time lag suggests that processes other than dilution were important in the catchments studied. Daily discharge patterns had a negligible effect on concentration dynamics during rain events, as shown in S1 Fig. 2 for ammonium in catchment M. Therefore, we hypothesize that one of the following processes could explain the time-shifted dilution process:

- Remobilization of sewer deposits triggered by increased flow rates can release organic chemicals that have accumulated during dry periods. For example, resuspension of sewer sediments was found to be a significant source for carbamazepine, a wastewater pollutant with LogK_{ow} of 2.45 [8,9]. However, polar chemicals are unlikely to accumulate in sewer sediments.
- Stormwater parcels could potentially arrive earlier in combined sewer systems or be pushed forward by wastewater parcels. In this case, the flow increases because "fast" stormwater parcels arrive before the municipal wastewater is diluted.

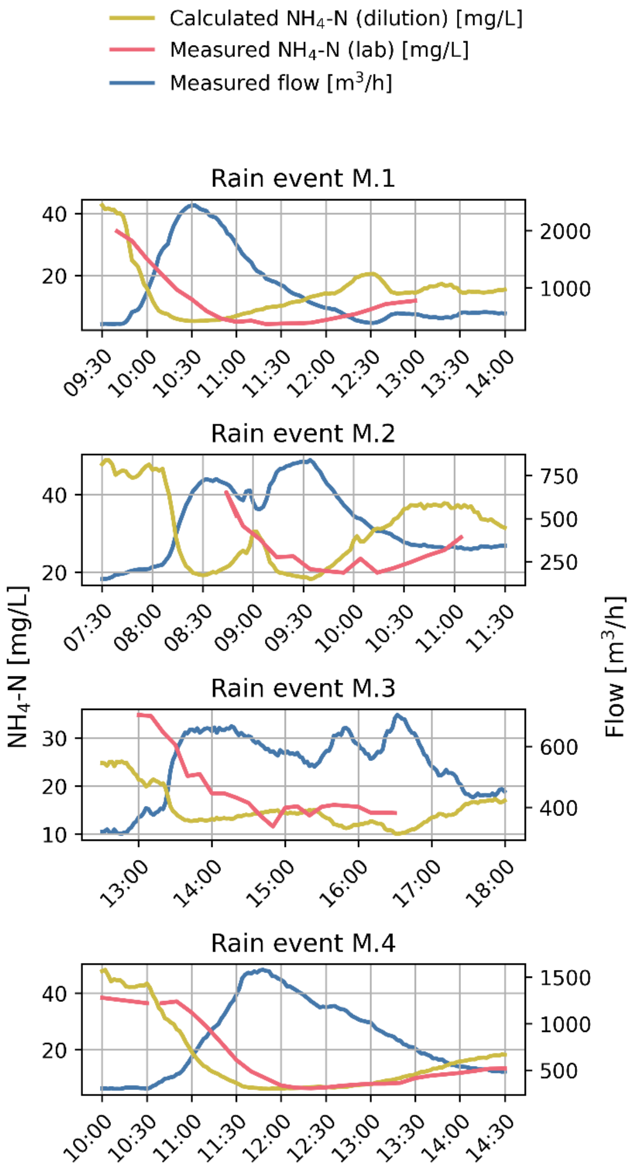

S1 Fig. 2. Measured vs. calculated dilution of NH_4-N in catchment M. Shown are the measured flow (blue), the measured NH_4-N concentrations (red), and the NH_4-N concentrations calculated (yellow) based on dilution of the average dry weather NH_4-N load by the wet weather flow. The figure shows that the measured minimum NH_4-N concentration occurred later than the minimum NH_4-N concentration calculated based on the daily pattern of the NH_4-N load and dilution by stormwater.

S1 Fig. 2 shows the dilution time lag for NH_4-N in catchment M. We calculated the expected NH_4-N patterns based on the dilution of the average dry weather NH_4-N load by the observed wet weather flow:

$$NH_{4,calc.}\left[\frac{mg}{L}\right] = \frac{dry\ flow\left[\frac{m^3}{h}\right] \times dry\ NH_4\left[\frac{mg}{L}\right]}{wet\ flow\ \left[\frac{m^3}{h}\right]}.$$

S1 Fig. 2 shows that the observed NH_4-N minima occur later than expected. This suggests that dilution of wastewater by stormwater alone cannot account for the observed minimum concentrations.

For every rain event, the exact length of the time lag was determined by correlation analysis. Therefore, Pearson correlation coefficients (PCC) were calculated for correlations between organic chemicals from municipal wastewater and time-shifted level and flow. Level and flow were shifted from -3 to 0 hours in 10-minute intervals. For each chemical and rain event, the time shift with the strongest negative correlation was selected. Then, the average time lag was calculated for each indoor chemical and the entire

group across all rain events, considering only significant correlations (|PCC| ≥ 0.5, S1 Table 10). The average time shift of all indoor chemicals was approx. 60 min in catchment S, approx. 40 min in catchment M, and approx. 50 min in catchment L when assessed based on flow measurements (S1 Table 12). Level and flow shifted by the average time shift were included in the predictor set of the SLR and MLR models for indoor chemicals. The statistical measures of the correlations between each indoor chemical and level and flow shifted by the time shift shown in S1 Table 10 are displayed in S2 Tables 1-3.

S1 Table 10. Time shift of dilution for every indoor chemical in catchments S, M, and L. The mean time shift (Mean) and standard deviation (STD) were calculated across all rain events in every catchment for every chemical (group) and parameter for which |PCC| ≥ 0.5. The minus sign indicates that maximum level/flow values occurred before minimum chemical concentrations.

	**Catchment S**				**Catchment M**				**Catchment L**	
	**Level**		**Flow**		**Level**		**Flow**		**Flow**	
	**Mean [min]**	**STD [min]**	**Mean [min]**	**STD [min]**	**Mean [min]**	**STD [min]**	**Mean [min]**	**STD [min]**	**Mean [min]**	**STD [min]**
**ACE**	-50	62	-51	65	-53	28	-58	30	-38	48
**CAF**	-62	67	-37	54	-40	37	-43	42	-49	64
**CYC**	-55	57	-68	63	-50	31	-50	36	-44	50
**CAN**	-92	70	-88	68	-55	33	-58	36	-50	60
**CIT**	-117	59	-30	30	-28	13	-30	14	-35	50
**DCF**	-28	45	-53	68	-43	16	-43	19	-65	65
**HCT**	-60	55	-85	74	-48	20	-45	19	-49	59
**TCS**	-94	73	-94	66	-30	0	-30	0	-64	62

7. Hierarchical clustering of organic chemicals from catchments of different sizes

Clustering algorithm. To investigate whether organic chemicals originating from the same urban source exhibit similar dynamics, a hierarchical clustering algorithm was used for grouping. The clustering algorithm used Ward's variance minimization algorithm to group the chemicals into four clusters. The distance between two clusters was calculated as:
$d(u,v) = \sqrt{\frac{|v|+|s|}{T} d(v,s)^2 + \frac{|v|+|t|}{T} d(v,t)^2 + \frac{|v|}{T} d(s,t)^2}$, where u is the newly joined cluster consisting of clusters s and t, v is an unused cluster in the forest, $T = |v| + |s| + |T|$, and $|*|$ is the cardinality of its argument [10]. Four clusters were chosen to reflect the chemical groups: indoor chemicals, chemicals from road runoff, PPPs and biocides, and chemicals from diverse sources.

Data pretreatment. As the clustering algorithms used cannot handle missing data, values below the LOQ were replaced by the value LOQ/2 [11]. The data were normalized using the Z-score to put the concentrations of all substances during all rain events on the same scale. Each rain event was normalized separately (with its respective mean and standard deviation) because the chemical concentrations varied between the rain events. The Z-score could not be calculated if a chemical was not detected during a rain event. A value of 0 was then used.

Results. S1 Table 11 shows the cluster assignments. S1 Figs. 3-5 show that organic chemicals in the same cluster show similar temporal dynamics. Thus, the clustering of organic chemicals from the same group into one cluster identifies source-specific behavior.

S1 Table 11. Results of hierarchical clustering of organic chemicals measured in catchments S, M, and L. In the table, each chemical group is represented in a distinct color: indoor chemicals are shown in orange, chemicals from road runoff in blue, PPPs and biocides in purple, and chemicals from diverse sources in green.

	Catchment S	Catchment M	Catchment L
Cluster 1	DPG, 6PPDQ, HMMM	DPG, 6PPDQ, HMMM	DPG, 6PPDQ, HMMM
Cluster 2	ACE, CAF, CYC, CIT, HCT	ACE, CAF, CYC, CAN, CIT, DCF, HCT	ACE, CAF, CYC, CAN, CIT, DCF, HCT, TCS
	BT		BT, DEET
Cluster 3	DCF, TCS	2,4-D, DCMU, MCPA, MPP, OIT	2,4-D, MCPA, MPP
	MPP		MeBT
Cluster 4	CAN	TCS	DCMU, OIT
	2,4-D, CBZ, DCMU, MCPA, OIT	CBZ	
	MeBT, DEET	MeBT, BT, DEET	

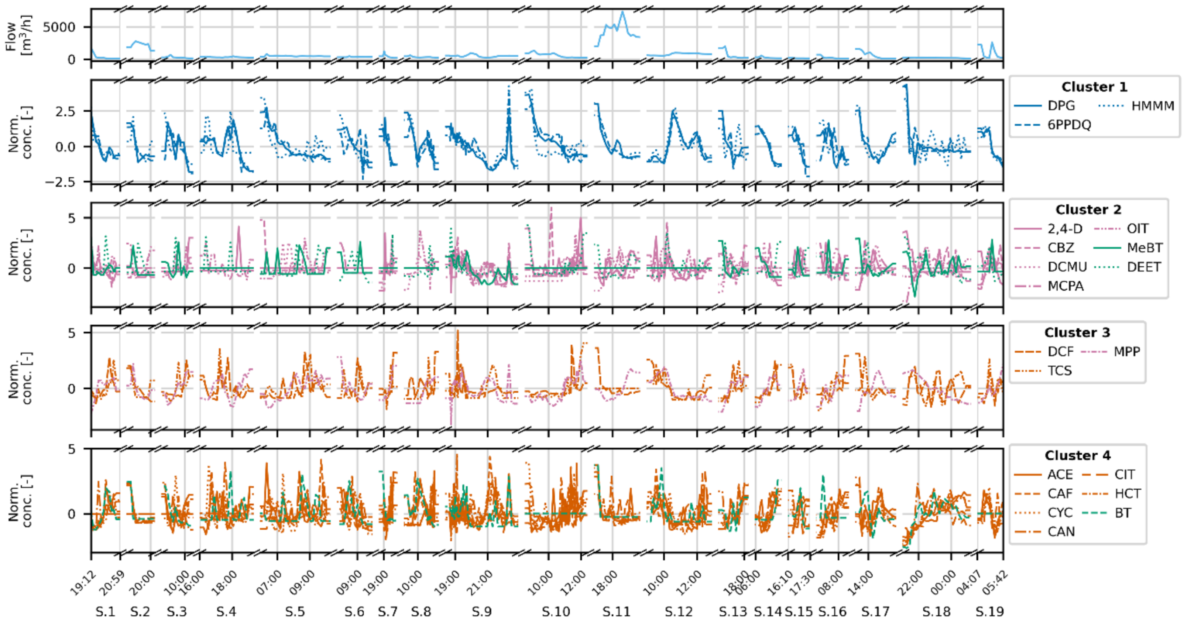

S1 Fig. 3. Hierarchical clustering of organic chemicals measured in catchment S. The hierarchical clustering algorithm used Ward's distance, and the number of clusters was set to four. Clustering was applied to normalized chemical concentrations (Z-score). Indoor chemicals are shown in orange, chemicals from road runoff in blue, PPPs and biocides in pink, and chemicals from diverse sources in green.

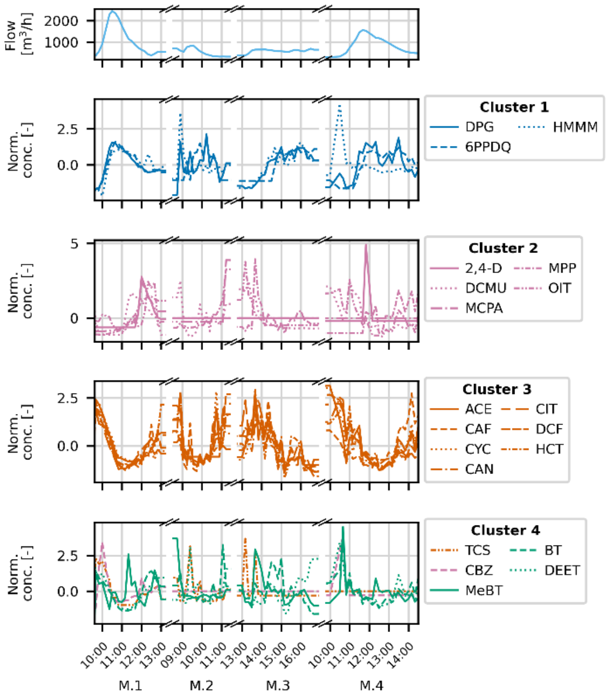

S1 Fig. 4. Hierarchical clustering of organic chemicals measured in catchment M. The hierarchical clustering algorithm used Ward's distance, and the number of clusters was set to four. Clustering was applied to normalized chemical concentrations (Z-score). Indoor chemicals are shown in orange, chemicals from road runoff in blue, PPPs and biocides in pink, and chemicals from diverse sources in green.

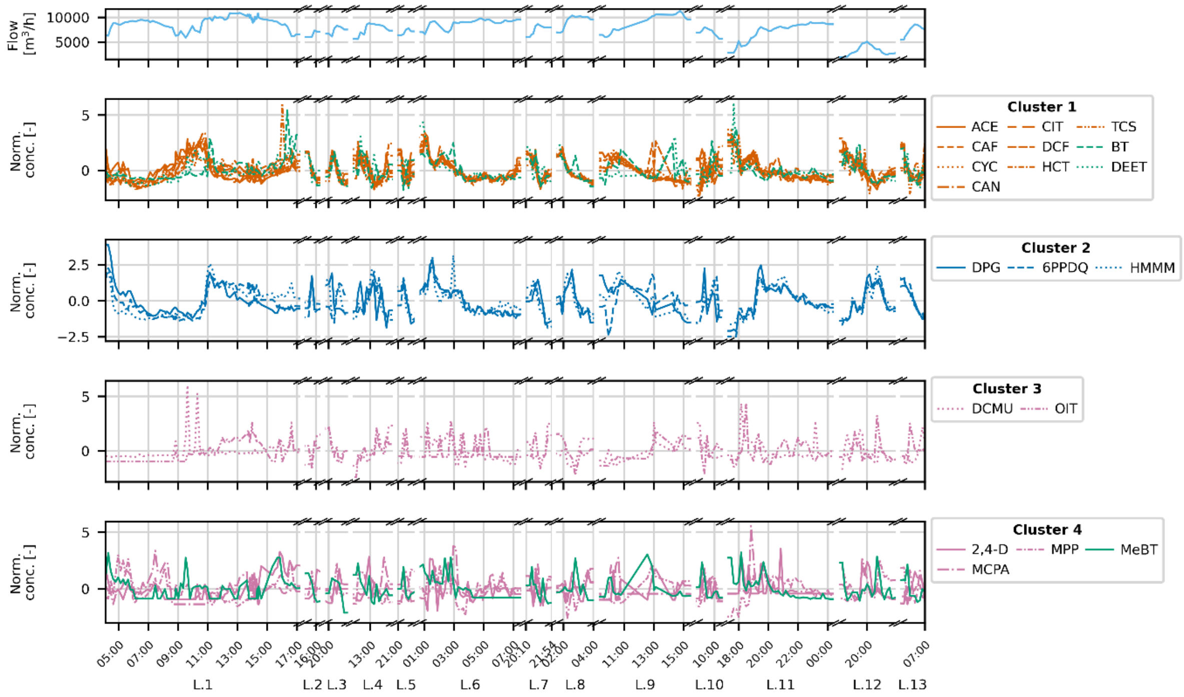

S1 Fig. 5. Hierarchical clustering of organic chemicals measured in catchment L. The hierarchical clustering algorithm used Ward's distance, and the number of clusters was set to four. Clustering was applied to normalized chemical concentrations (Z-score). Indoor chemicals are shown in orange, chemicals from road runoff in blue, PPPs and biocides in pink, and chemicals from diverse sources in green.

8. Correlations between organic chemicals of one chemical group

Correlation plot distinguishing PPPs and biocides. We grouped the organic chemicals into five groups according to their expected source (indoor, road, PPPs, biocides, and diverse). Then, the Pearson correlation coefficient for all substances within one group was calculated for each catchment. High median correlation coefficients indicate that the chemicals within one group exhibit similar temporal patterns, suggesting source-specific behavior. The median correlation coefficients of PPPs and biocides are low (S1 Fig. 6), indicating that these substances show different temporal dynamics. Thus, they are treated as one combined group in the analyses of the manuscript.

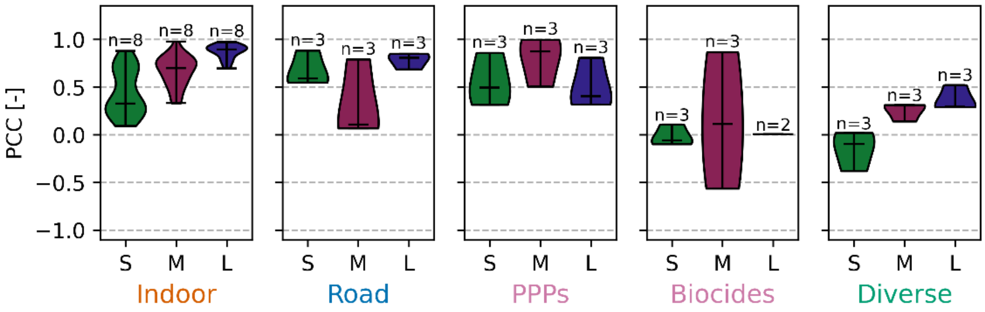

S1 Fig. 6. Correlations between organic chemicals of one chemical group. The figure displays the Pearson correlation coefficients (PCCs) for correlations between polar organic chemicals within each chemical group (indoor, road, PPPs, biocides, and diverse) across all rain events for catchments S, M, and L. The bar indicates the median of each group and catchment. *n* is the number of substances in each catchment and group. Further statistical measures of the correlations (*p*-value, confidence interval, etc.) can be found in S2 Tables 4-6. Abbreviation: PPPs: plant protection products.

Mass vs. volume curves for catchment M. S1 Fig. 7 shows the mass vs. volume M(v) curves for organic chemicals from stormwater (road runoff, PPPs and biocides) in catchment M. According to the definition of Betrand-Kraewski [12], a first flush occurs when at least 80% of the total pollutant mass is transported in the first 30% of the discharge volume. For this analysis, concentrations below the limit of quantification were replaced by LOQ/2 [11]. No organic chemicals from road runoff showed a first flush in catchment M (S1 Fig. 7). However, a higher proportion of HMMM mass was transported at the beginning of rain events M.2 and M.4 compared to the mass of 1,3-diphenylguanidine and 6PPD-quinone (S1 Fig. 7). PPPs and biocides exhibited diverse mobilization patterns depending on the substance and rain event (S1 Fig. 7). Diuron and OIT showed a first flush during rain event M.3 (S1 Fig. 7). M(v) curves for outdoor chemicals in catchments S and L can be found in Furrer et al. [6].

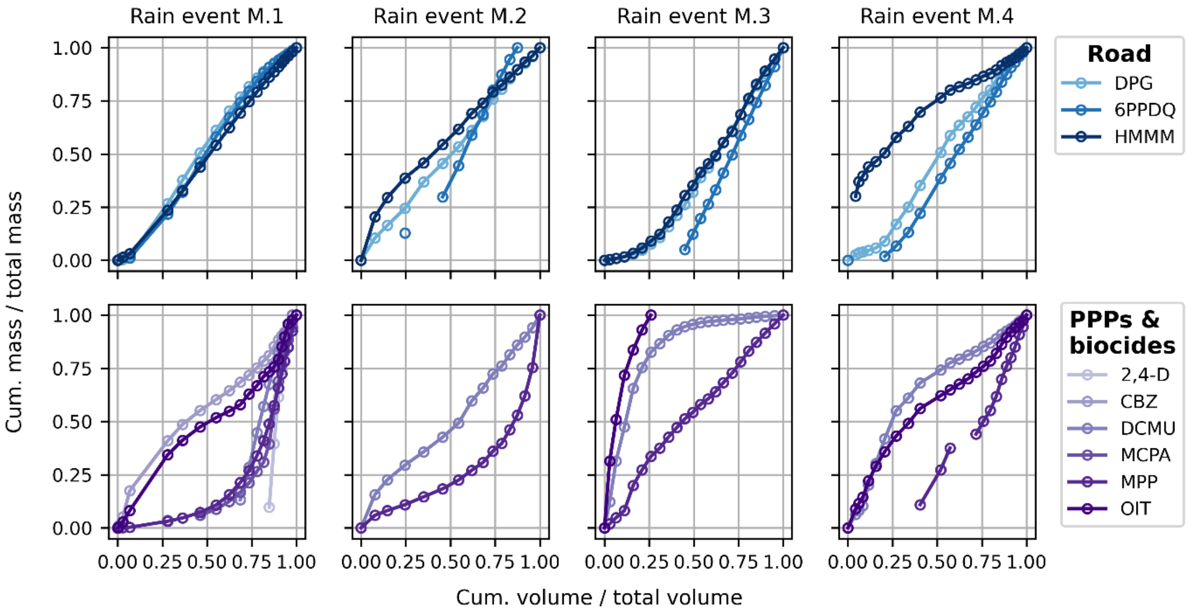

S1 Fig. 7. Mass vs. volume M(v) curves for organic chemicals from stormwater in catchment M. The upper row shows the M(v) curves for organic chemicals from road runoff and the bottom row shows the M(v) curves for PPPs and biocides. Abbreviation: PPPs: plant protection products.

9. Temporal dynamics of sensor parameters

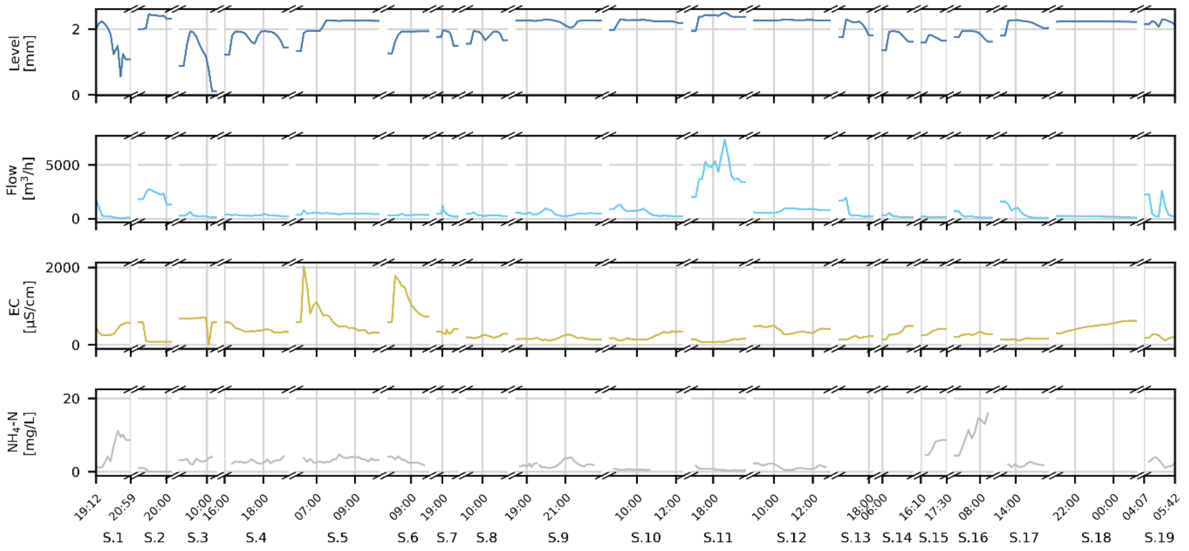

S1 Fig. 8. Temporal dynamics of sensor parameters measured in catchment S. S1 Table 7 shows more information about the sensor parameters shown here.

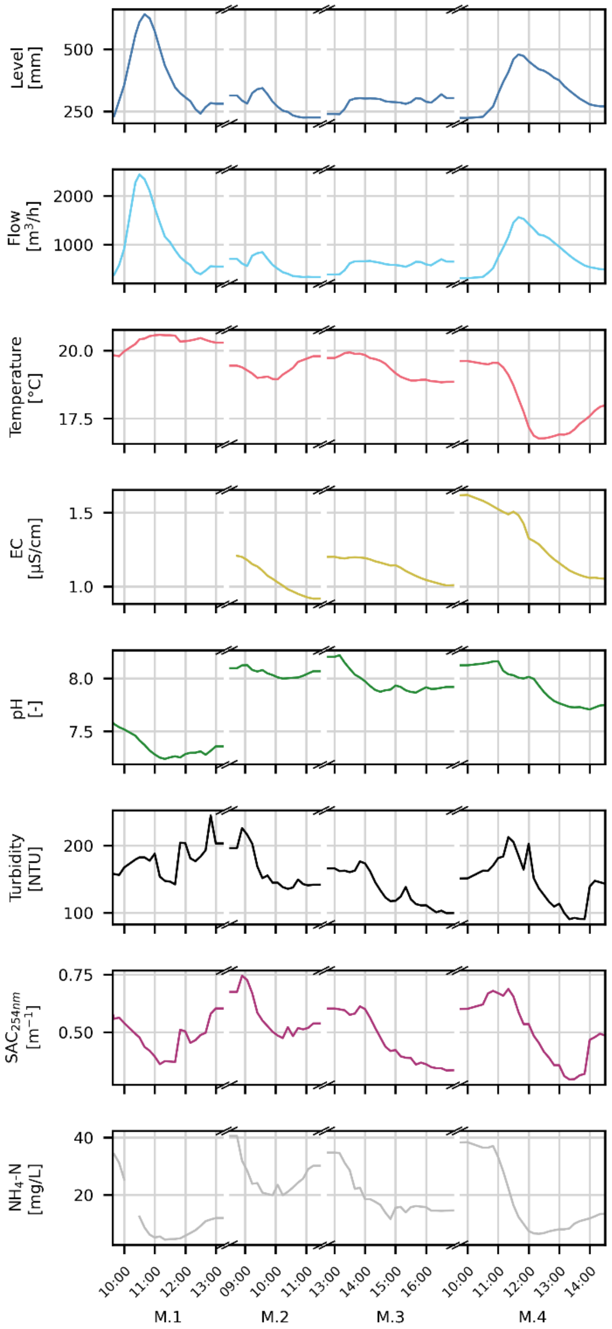

S1 Fig. 9. Temporal dynamics of sensor parameters measured in catchment M. S1 Table 7 shows more information about the sensor parameters shown here.

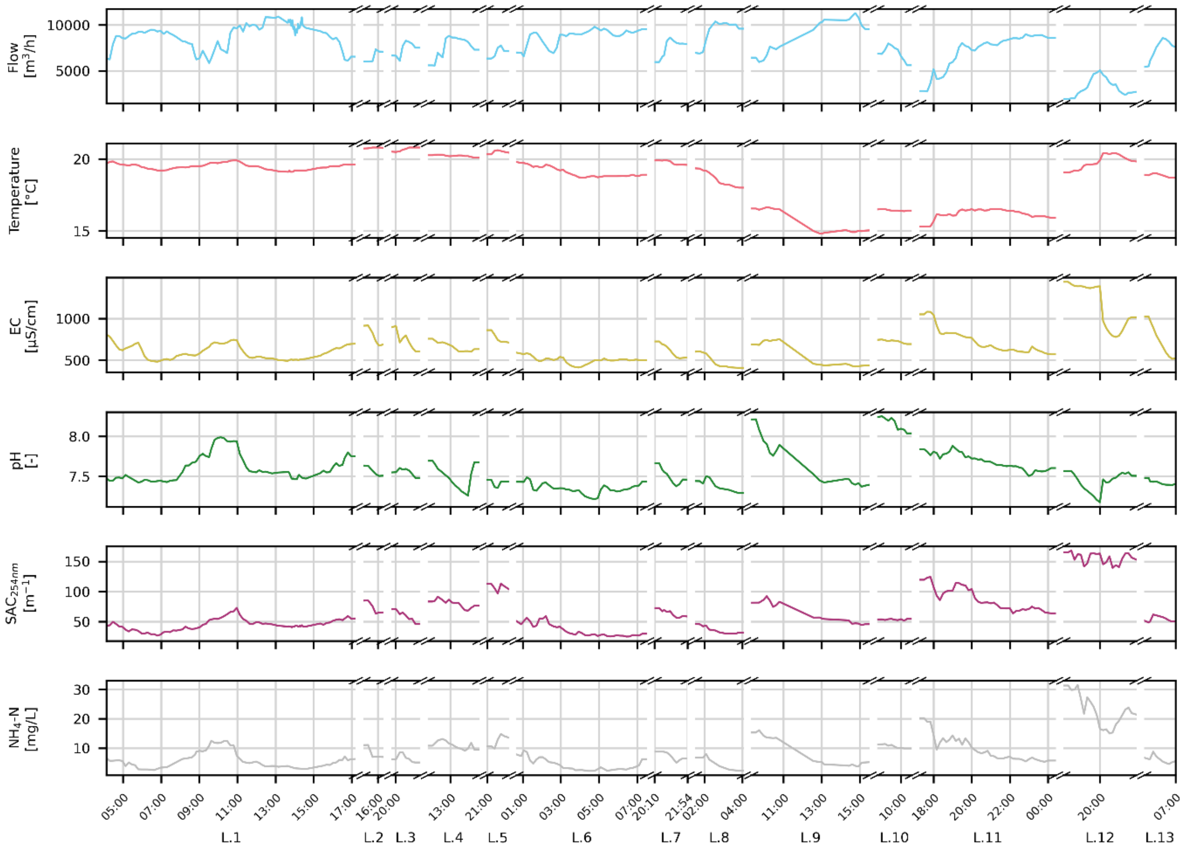

S1 Fig. 10. Temporal dynamics of sensor parameters measured in catchment L. S1 Table 7 shows more information about the sensor parameters shown here.

10. Correlations between organic chemicals and sensor parameters

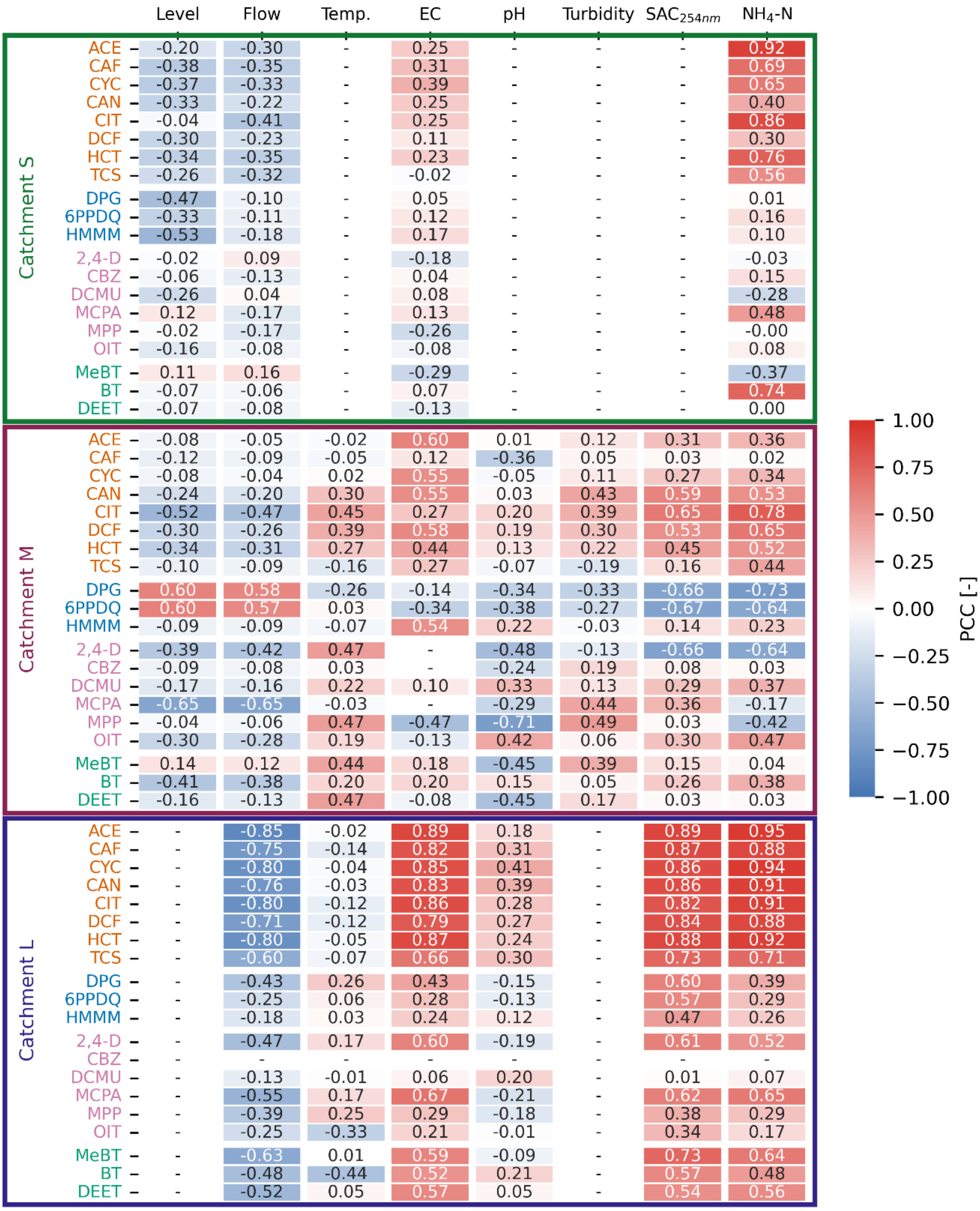

S1 Fig. 11. Correlations between organic chemicals and sensor parameters. The figure shows the Pearson correlation coefficients (PCC) between organic chemicals and sensor parameters in catchments S, M, and L. Positive correlation coefficients are colored red and negative correlations are colored blue. "-" indicates that a correlation coefficient could not be calculated because insufficient data were available. Further statistical measures of the correlations (*p*-value, confidence interval, etc.) can be found in S2 Tables 10-12. Abbreviations: EC: electrical conductivity, NH_4-N: ammonium, $SAC_{254\ nm}$: spectral absorption coefficient at 254 nm.

We grouped the organic chemicals into four groups according to their expected source (indoor, road, PPPs & biocides, and diverse). Then, the Pearson correlation coefficient between all substances within one group and each sensor parameter was calculated for every catchment. High median correlation coefficients indicate that a sensor parameter can serve as a proxy for a group of organic chemicals. S1 Fig. 12 displays the same information as S1 Fig. 11, but shows the distribution of the correlation coefficients for each group and their medians. Further statistical measures of the correlations (*p*-value, confidence interval, etc.) can be found in S2 Tables 10-12.

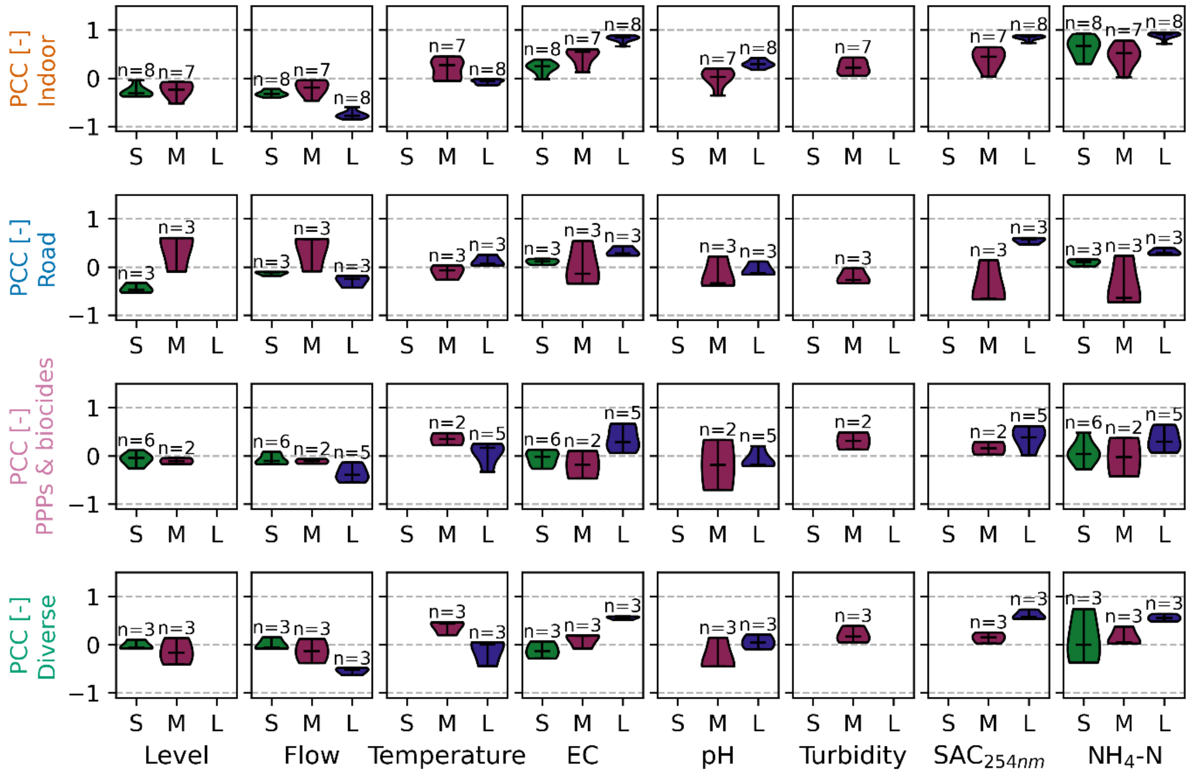

S1 Fig. 12. Correlation coefficients between organic chemicals of one chemical group and sensor parameter. The figure shows the Pearson correlation coefficients (PCC) between organic chemicals of one substance group (indoor, road, PPPs & biocide, and diverse) and every sensor parameter in catchments S, M, and L. The bar indicates the median of each group and catchment. *n* is the number of correlations summarized in one violin plot. Further statistical measures of the correlations (*p*-value, confidence interval, etc.) can be found in S2 Tables 10-12. Abbreviations: EC: electrical conductivity, NH_4-N: ammonium, $SAC_{254\ nm}$: spectral absorption coefficient at 254 nm, PPPs: plant protection products.

S1 Fig. 13 shows Pearson correlation coefficients between organic chemicals from municipal wastewater and time-shifted level and flow. They were calculated because we identified a time shift in dilution in S1 Fig. 1. For some indoor chemicals, correlations were stronger with time-shifted level or flow than with unshifted level or flow (S1 Fig. 11).

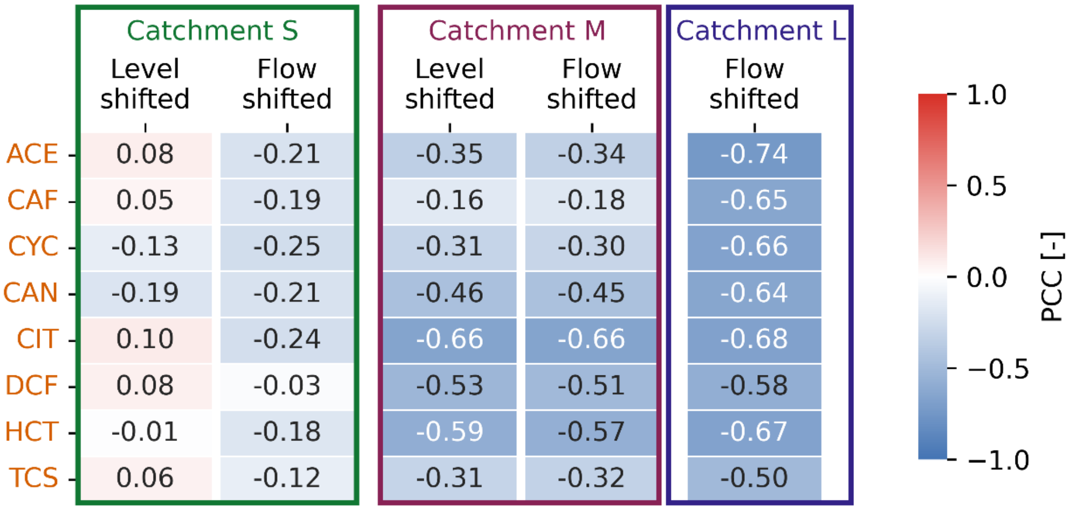

S1 Fig. 13. Correlations between indoor chemicals and time-shifted level and flow. The figure shows the Pearson correlation coefficients (PCC) between organic chemicals and time-shifted level and flow in catchments S, M, and L. Positive correlation coefficients are colored red and negative correlations are colored blue. The statistical measures of the correlations (*p*-value, confidence interval, etc.) displayed in this figure are shown in S2 Tables 13-15. S1 Table 12 shows the average time shift for indoor chemicals in every catchment that was used for this calculation.

Additionally, we calculated correlations between every chemical group and time-shifted flow and level to assess whether time-lagged flow or level could serve as proxies for these chemical groups. Pearson correlation coefficients (PCC) were calculated for correlations between organic chemicals and time-shifted level and flow. Level and flow were shifted in 10-minute intervals from -3 to 0 hours for indoor chemicals (dilution time lag) and from -3 to +3 hours for the other chemical groups. For each chemical and rain event, the time shift with the strongest correlation was selected. Then, the average time shift across all substances within a chemical group and rain events was calculated, considering only significant correlations (|PCC| ≥ 0.5) (S1 Table 12).

S1 Table 12. Mean time shift between chemical concentrations and time-shifted flow or level for each chemical group in catchments S, M, and L. The mean time shift (Mean) and standard deviation (STD) were calculated over all rain events in every catchment for every chemical (group) and parameter for which |PCC| ≥ 0.5. The minus sign indicates that level/flow is shifted backward in time compared to organic chemical concentrations.

	Catchment S				Catchment M				Catchment L	
	Level		Flow		Level		Flow		Flow	
	Mean [min]	STD [min]	Mean [min]	STD [min]	Mean [min]	STD [min]	Mean [min]	STD [min]	Mean [min]	STD [min]
**Indoor**	-70	67	-63	67	-43	28	-44	27	-49	58
**Road**	-25	69	-64	91	63	62	77	47	-33	89
**PPPs & biocides**	0.3	106	-4	104	-12	32	-13	32	9	113
**Diverse**	12	108	21	122	-33	59	-35	59	-30	74

As explained in the manuscript section 2.5 Data analysis, correlations between a chemical group and a sensor parameter were calculated using normalized values (Z-score). S1 Fig. 14 shows the correlations (PCC) between each indoor chemical group and level and flow shifted by the average time shift (S1 Table 12). Most correlations are weak (PCC < 0.5), indicating that time-shifted level and flow are not suitable sensor proxies for the chemical groups studied, except for indoor chemicals, where we observed a time lag in dilution (S1 Fig. 1, S1 Fig. 13).

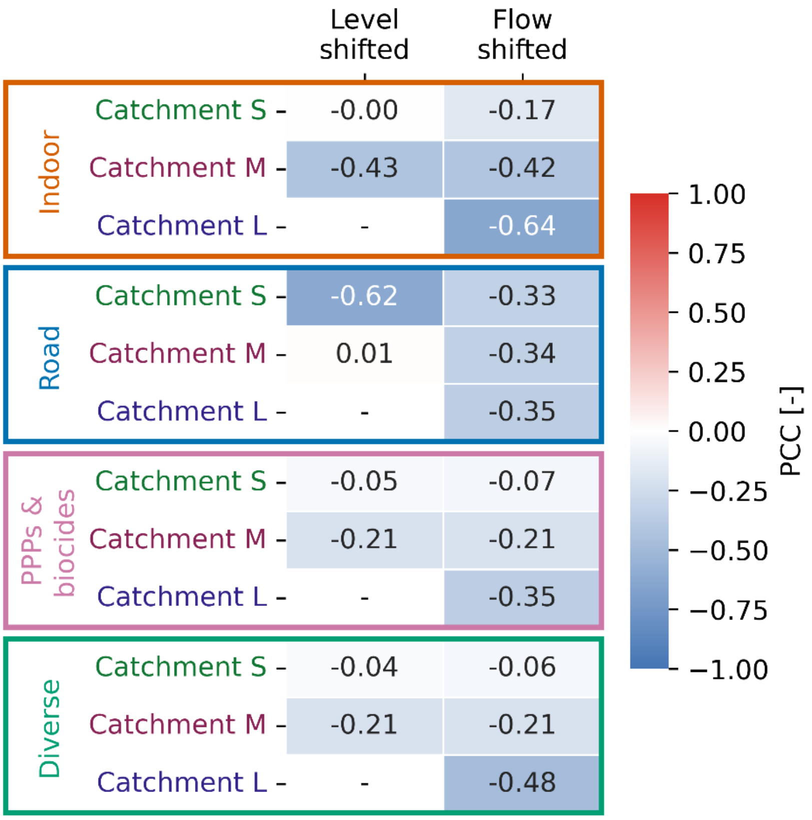

S1 Fig. 14. PCCs for correlations between polar organic chemicals of each chemical group and time-shifted level and flow. This figure shows the PCCs for correlations between polar organic chemicals within each chemical group (indoor, road, PPPs & biocides and diverse) and time-shifted level and flow across all rain events for the catchments S, M, and L. Positive PCCs are colored red, and negative PCCs are colored blue. “-” indicates that a correlation coefficient could not be calculated because sensor data were not available. Further statistical measures of the correlations (*p*-value, confidence interval, etc.) can be found in S2 Tables 13-15. Abbreviation: PCC: Pearson correlation coefficient.

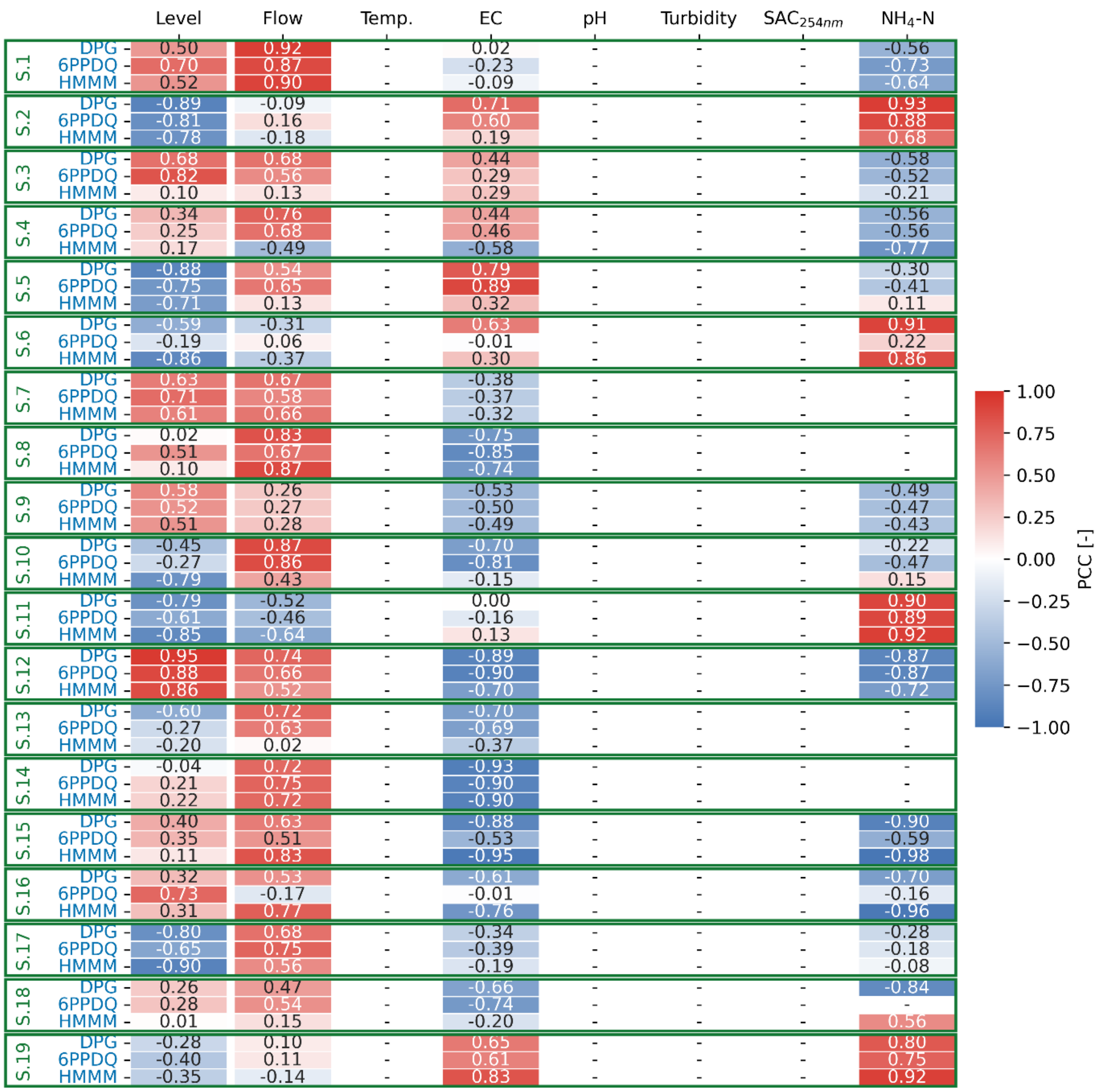

S1 Fig. 15. Correlations between organic chemicals from road runoff and sensor parameters in catchment S. The figure shows the Pearson correlation coefficients (PCC) between organic chemicals from road runoff and sensor parameters during all rain events in catchment S. Positive correlation coefficients are colored red and negative correlations are colored blue. “-” indicates that a correlation coefficient could not be calculated because not enough data were available. Further statistical measures of the correlations (*p*-value, confidence interval, etc.) can be found in S2 Table 16. Abbreviations: EC: electrical conductivity, NH_4-N: ammonium, $SAC_{254\,nm}$: spectral absorption coefficient at 254 nm.

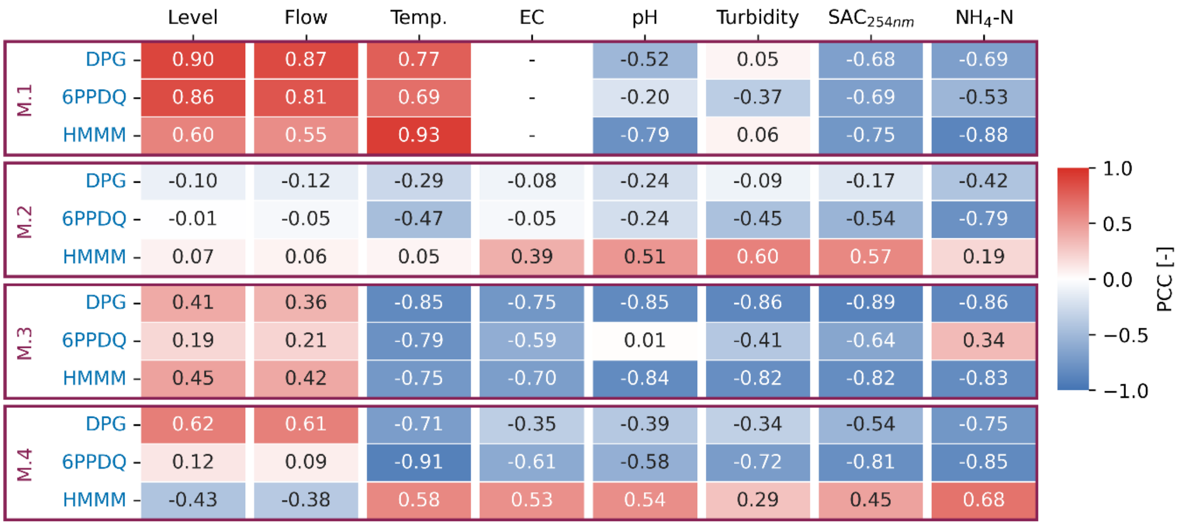

S1 Fig. 16. Correlations between organic chemicals from road runoff and sensor parameters in catchment M. The figure shows the Pearson correlation coefficients (PCC) between organic chemicals from road runoff and sensor parameters during all rain events in catchment M. Positive correlation coefficients are colored red and negative correlations are colored blue. “-” indicates that a correlation coefficient could not be calculated because not enough data were available. Further statistical measures of the correlations (*p*-value, confidence interval, etc.) can be found in S2 Table 17. Abbreviations: EC: electrical conductivity, NH_4-N: ammonium, $SAC_{254\ nm}$: spectral absorption coefficient at 254 nm.

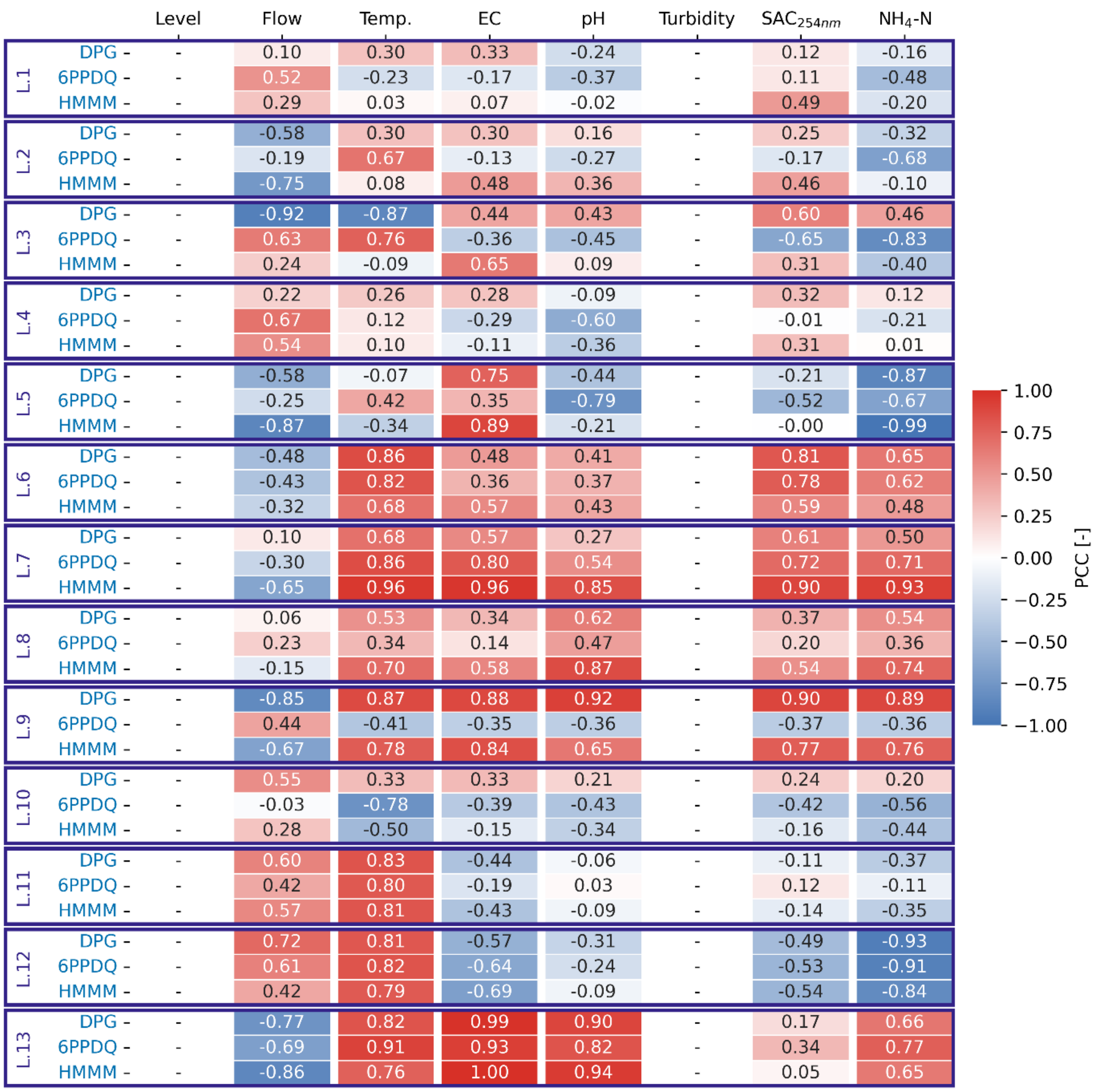

S1 Fig. 17. Correlations between organic chemicals from road runoff and sensor parameters in catchment S. The figure shows the Pearson correlation coefficients (PCC) between organic chemicals from road runoff and sensor parameters during all rain events in catchment S. Positive correlation coefficients are colored red and negative correlations are colored blue. "-" indicates that a correlation coefficient could not be calculated because not enough data were available. Further statistical measures of the correlations (*p*-value, confidence interval, etc.) can be found in S2 Table 18. Abbreviations: EC: electrical conductivity, NH_4-N: ammonium, $SAC_{254\ nm}$: spectral absorption coefficient at 254 nm.

11. Correlations between organic chemicals and UV-Vis absorbance in catchment M

Section 3.2 Correlations between polar organic chemicals and sensor parameters of the manuscript examines correlations between organic chemicals and absorption at 254 nm, representing DOC. However, there may be molecules in wastewater or stormwater that are more specific than DOC and absorb light at other wavelengths. The UV-Vis spectrum was available from 228-708 nm in catchment M. Thus, we determined at which wavelength absorption correlated best with organic chemical concentrations. Most indoor chemicals and chemicals from road runoff exhibited the strongest correlations with absorption at wavelengths around 230 nm (for p-values < 0.05, S1 Table 13). Indoor chemicals correlated positively with absorption at 230 nm, while chemicals from road runoff correlated negatively. Thus, S1 Table 13 suggests that absorption in this range is a good proxy for most of these chemicals. This can be explained by the change in the UV-Vis spectrum of wastewater during rain events. Absorption initially declined and then increased proportionally to the wastewater proportion in the sewer. This was particularly evident within the 230 nm range, where the slope is steeper, and changes are more pronounced than at 254 nm (S1 Fig. 18). This segment of the spectra reflects human urine and anionic surfactants [13], which are diluted by stormwater. Thus, absorption at 230 nm might be a better proxy than the $SAC_{254\,nm}$ for indoor chemicals (except caffeine) and chemicals from road runoff (except HMMM) in sewer systems.

S1 Table 13. Wavelengths at which absorption correlated best with organic chemicals in catchment M. Correlation analysis was used to determine the wavelength at which absorption with organic chemicals was strongest. The Pearson correlation coefficients and other statistical measures are also given. This table shows the number of non-NaN observations used in the calculation (*n*), the Pearson correlation coefficient (*r*), the 95%-confidence interval (95% CI), and the p-value (*p*-val) for a two-sided correlation of an organic chemical (X) and light absorption at a wavelength [nm] (Y) and an alpha level of 0.05.

X	Y	n	r	95% CI	p-val
ACE	230 nm	85	0.05	-0.17 0.26	0.65
CAF	692 nm	85	0.28	0.08 0.47	<0.05
CYC	230 nm	85	0.61	0.46 0.73	<0.05
CAN	290 nm	63	0.64	0.46 0.76	<0.05
CIT	232 nm	85	0.58	0.42 0.71	<0.05
DCF	230 nm	85	0.51	0.33 0.65	<0.05
HCT	230 nm	24	0.24	-0.18 0.59	0.26
TCS	234 nm	85	-0.71	-1.38	<0.05
DPG	232 nm	60	-0.74	-1.42	<0.05
6PPDQ	230 nm	84	0.16	-0.05 0.37	0.13
HMMM	230 nm	9	-0.67	-0.93	0.05
2,4-D	236 nm	23	0.07	-0.35 0.47	0.76
CBZ	520 nm	77	0.22	-0.00 0.42	0.05
DCMU	230 nm	17	0.32	-0.19 0.70	0.2
MCPA	448 nm	74	0.34	0.12 0.53	<0.05
MPP	706 nm	53	0.07	-0.21 0.33	0.63
OIT	230 nm	85	0.1	-0.12 0.30	0.38
MeBT	612 nm	85	0.15	-0.06 0.35	0.17
BT	230 nm	85	0.04	-0.17 0.25	0.71

DEET	618 nm	85	0.75	0.63 0.83	<0.05

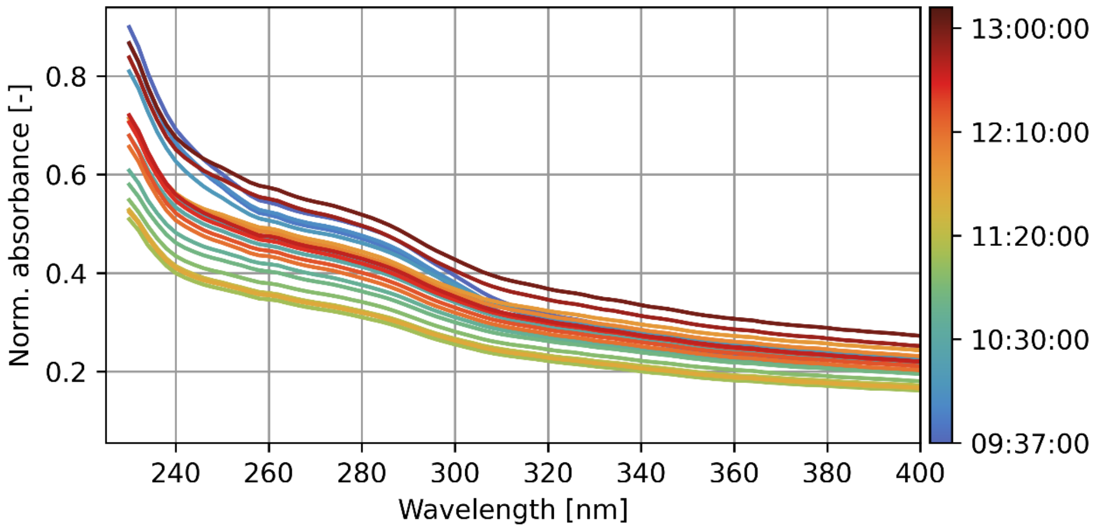

S1 Fig. 18. UV-Vis spectra of wastewater during rain event M.1. The figure shows how the UV-Vis spectra changed during rain event M.1. The spectra are normalized by the path length of the sensor. In the beginning of the rain event, when the wastewater proportion is high, the absorption is high. When the proportion of stormwater increases, absorption decreases. Finally, absorption increases again when the rain event ceases and the proportion of stormwater decreases. Similar patterns were also observed during other rain events.

12. Predicting organic chemical concentrations from sensor parameters

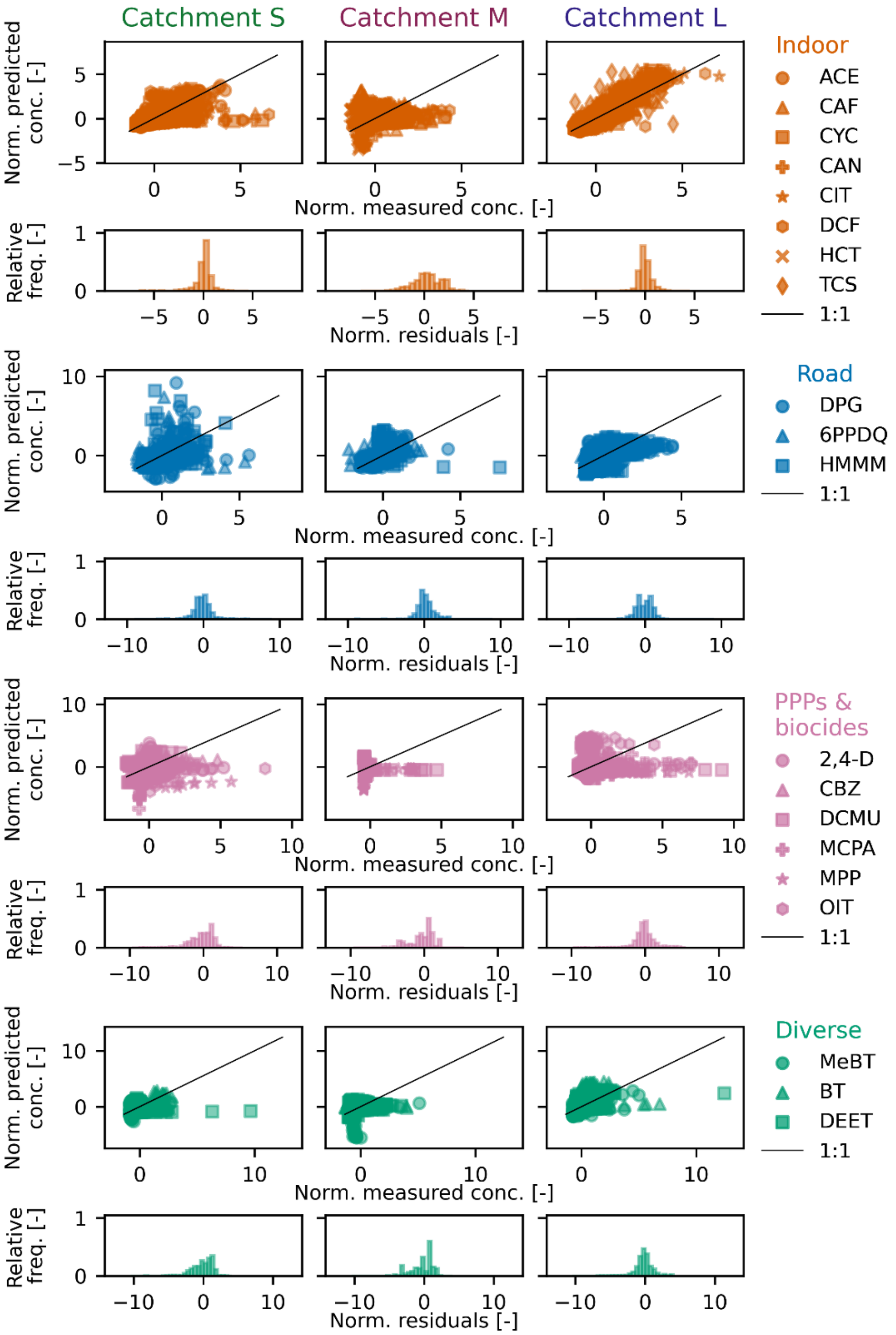

S1 Fig. 19. Predicted vs. measured concentrations and residual distributions of SLR models. Scatter plots show normalized (Z-score) predicted concentrations versus measured concentrations for each chemical group and catchment using SLR models. The black diagonal line represents perfect agreement (1:1 line). Corresponding histograms display the relative frequency of normalized residuals, illustrating the distribution of model errors for each chemical group and catchment. Table 4 in the manuscript shows which sensor was chosen for the SLR models of every chemical group. S3 Tables 22-38 show the predicted values of the SLR models displayed here.

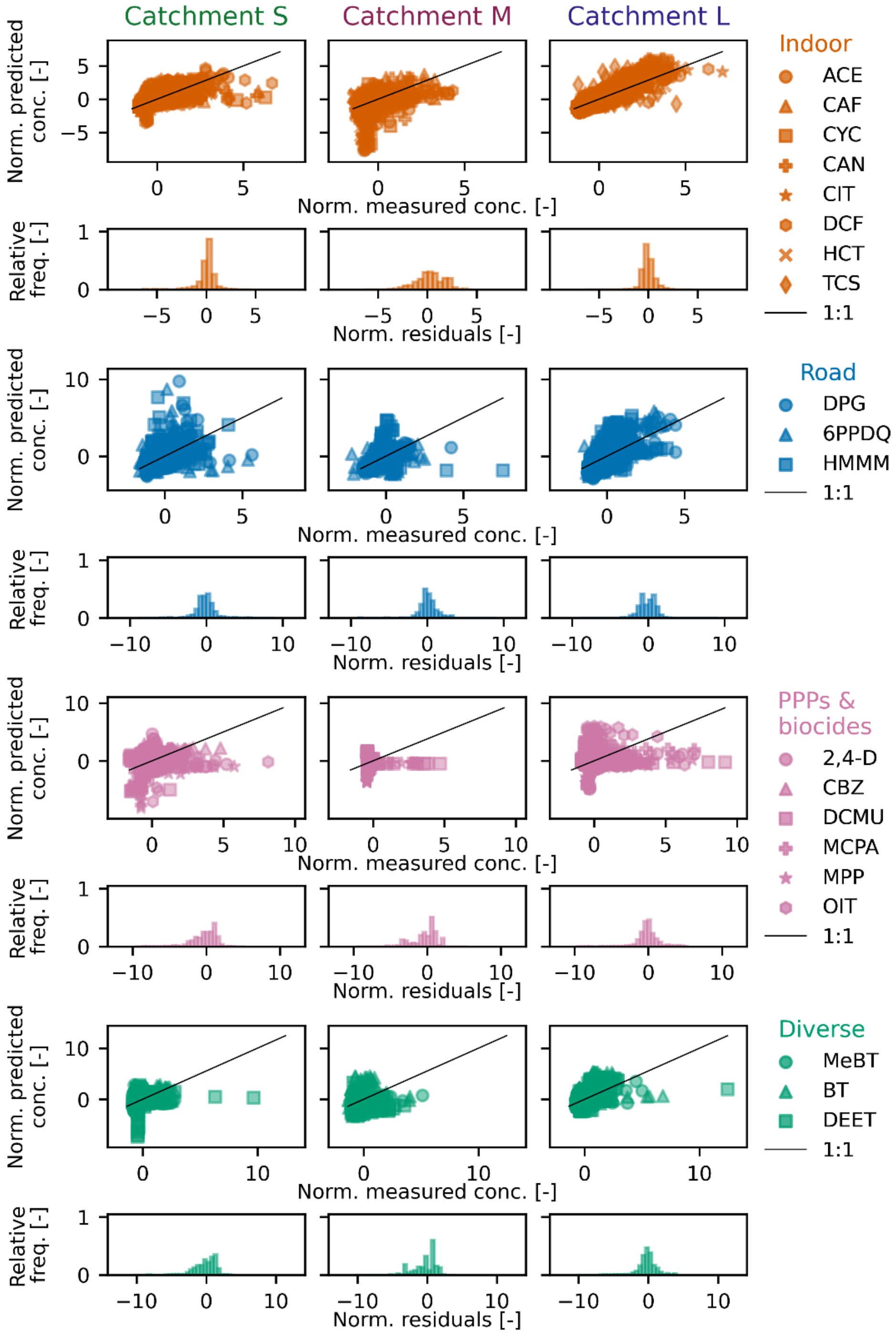

S1 Fig. 20. Predicted vs. measured concentrations and residual distributions of MLR models. Scatter plots show normalized (Z-score) predicted concentrations versus measured concentrations for each chemical group and catchment using MLR models. The black diagonal line represents perfect agreement (1:1 line). Corresponding histograms display the relative frequency of normalized residuals, illustrating the distribution of model errors for each chemical group and catchment. S3 Tables 39-41 show the predicted values of the MLR models displayed here. Abbreviation: PPPs: plant protection products.

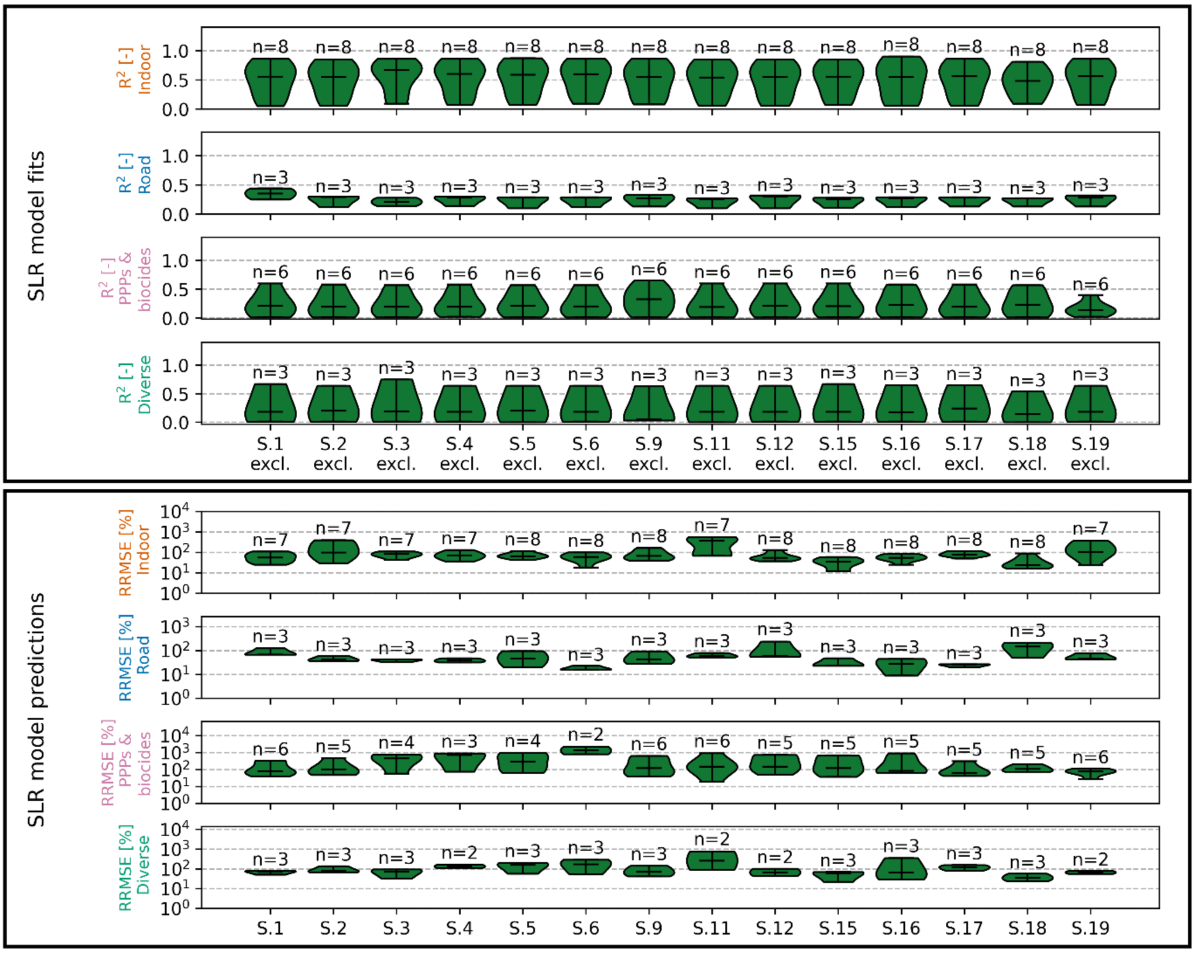

S1 Fig. 21. SLR model fits and predictions for catchment S. The violin plots in the upper panel display the R^2 of the model fits including all rain events except one in catchment S. The violin plots in the lower panel display the RRMSE of the model prediction for the excluded rain event. *n* indicates the number of substances > LOQ in every training set (upper panel)/testing set (lower panel). The horizontal line indicates the median. Table 4 in the manuscript shows which sensor was chosen for the SLR models of every chemical group. S3 Tables 1, 3 & 15 show further statistical measures of the SLR models displayed here. Abbreviations: SLR: simple linear regression, PPPs: plant protection products.

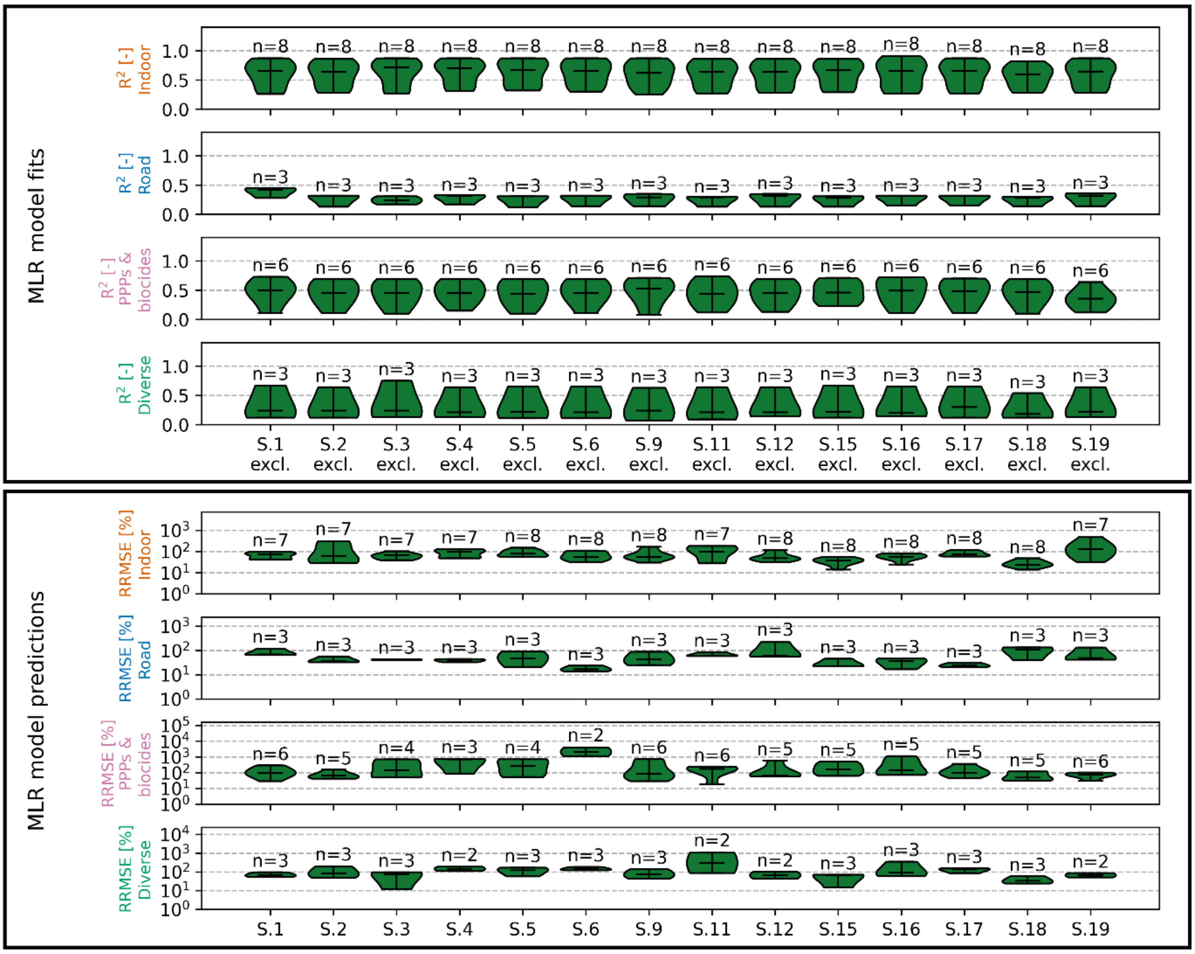

S1 Fig. 22. MLR model fits and predictions for catchment S. The violin plots in the upper panel display the R^2 of the model fits including all rain events except one in catchment S. The violin plots in the lower panel display the RRMSE of the model prediction for the excluded rain event. *n* indicates the number of substances > LOQ in every training set (upper panel)/testing set (lower panel). The horizontal line indicates the median. Table 4 in the manuscript shows which sensor was chosen for the MLR models of every chemical group. S3 Table 18 shows further statistical measures of the MLR models displayed here. Abbreviations: MLR: multiple linear regression, PPPs: plant protection products.

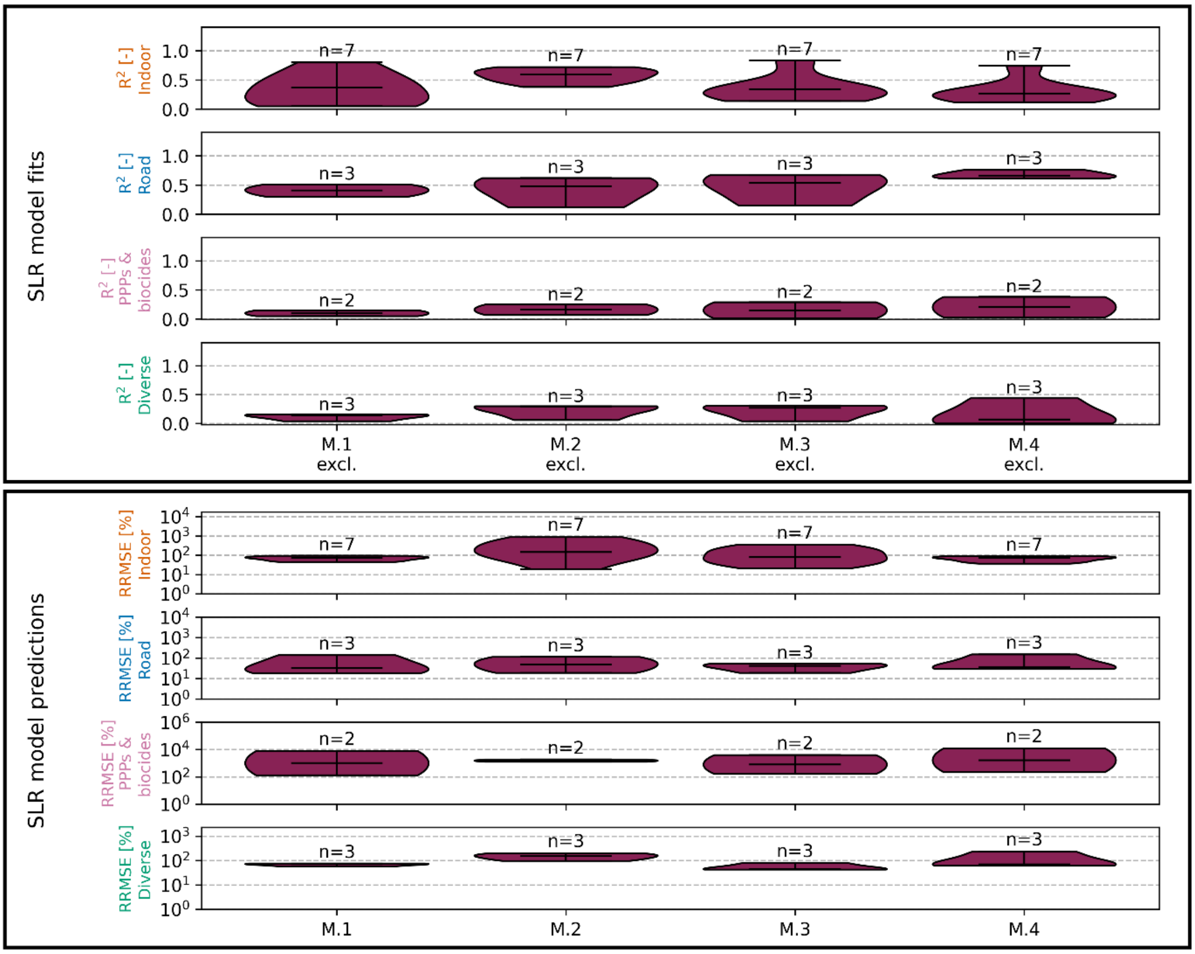

S1 Fig. 23. SLR model fits and predictions for catchment M. The violin plots in the upper panel display the R^2 of the model fits including all rain events except one in catchment M. The violin plots in the lower panel display the RRMSE of the model prediction for the excluded rain event. *n* indicates the number of substances > LOQ in every training set (upper panel)/testing set (lower panel). The horizontal line indicates the median. Table 4 in the manuscript shows which sensor was chosen for the SLR models of every chemical group. S3 Tables 2 & 6 show further statistical measures of the SLR models displayed here. Abbreviations: SLR: simple linear regression, PPPs: plant protection products.

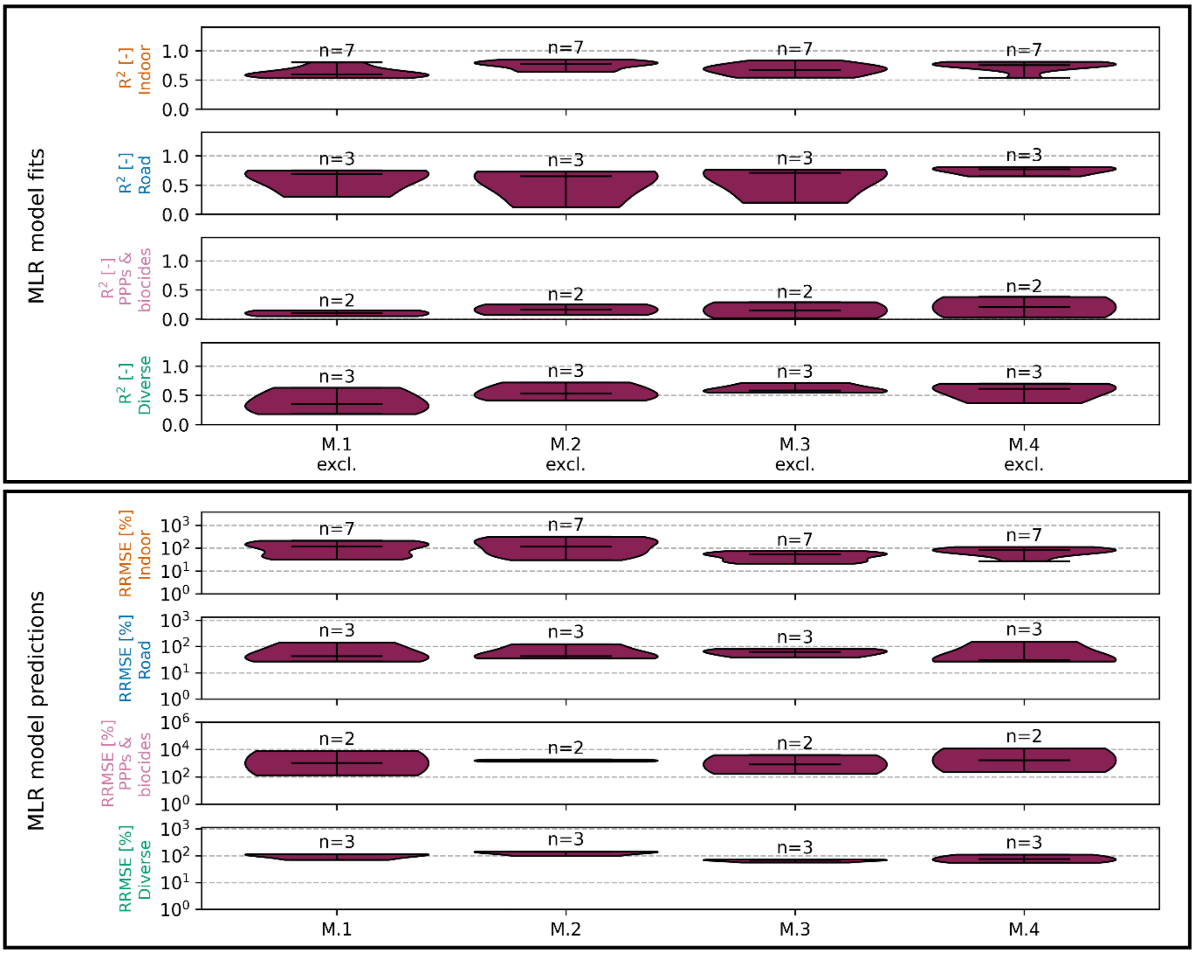

S1 Fig. 24. MLR model fits and predictions for catchment M. The violin plots in the upper panel display the R^2 of the model fits including all rain events except one in catchment M. The violin plots in the lower panel display the RRMSE of the model prediction for the excluded rain event. *n* indicates the number of substances > LOQ in every training set (upper panel)/testing set (lower panel). The horizontal line indicates the median. Table 4 in the manuscript shows which sensor was chosen for the MLR models of every chemical group. S3 Table 19 shows further statistical measures of the MLR models displayed here. Abbreviations: MLR: multiple linear regression, PPPs: plant protection products.

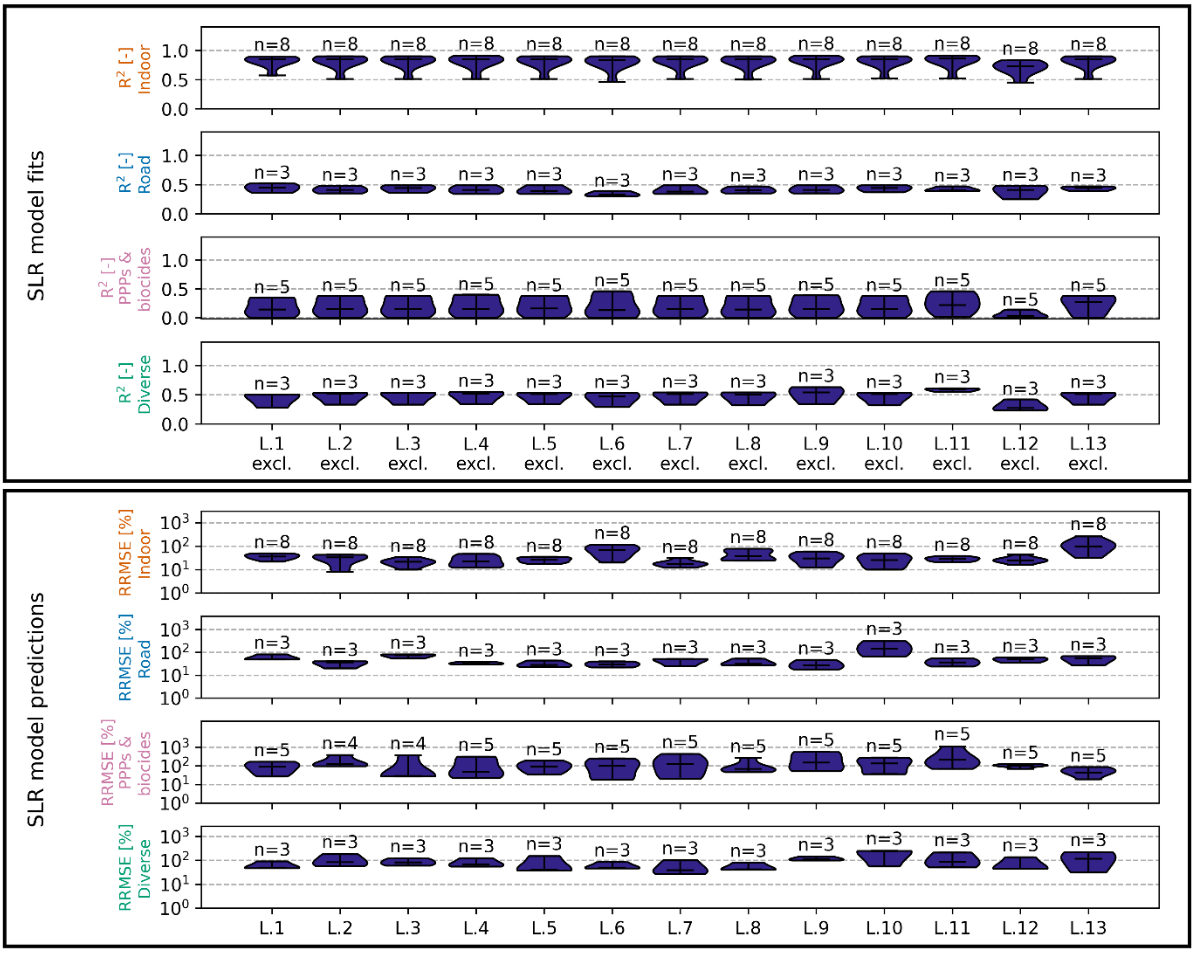

S1 Fig. 25. SLR model fits and predictions for catchment L. The violin plots in the upper panel display the R^2 of the model fits including all rain events except one in catchment L. The violin plots in the lower panel display the RRMSE of the model prediction for the excluded rain event. *n* indicates the number of substances > LOQ in every training set (upper panel)/testing set (lower panel). The horizontal line indicates the median. Table 4 in the manuscript shows which sensor was chosen for the SLR models of every chemical group. S3 Tables 5, 14 & 17 show further statistical measures of the SLR models displayed here. Abbreviations: SLR: simple linear regression, PPPs: plant protection products.

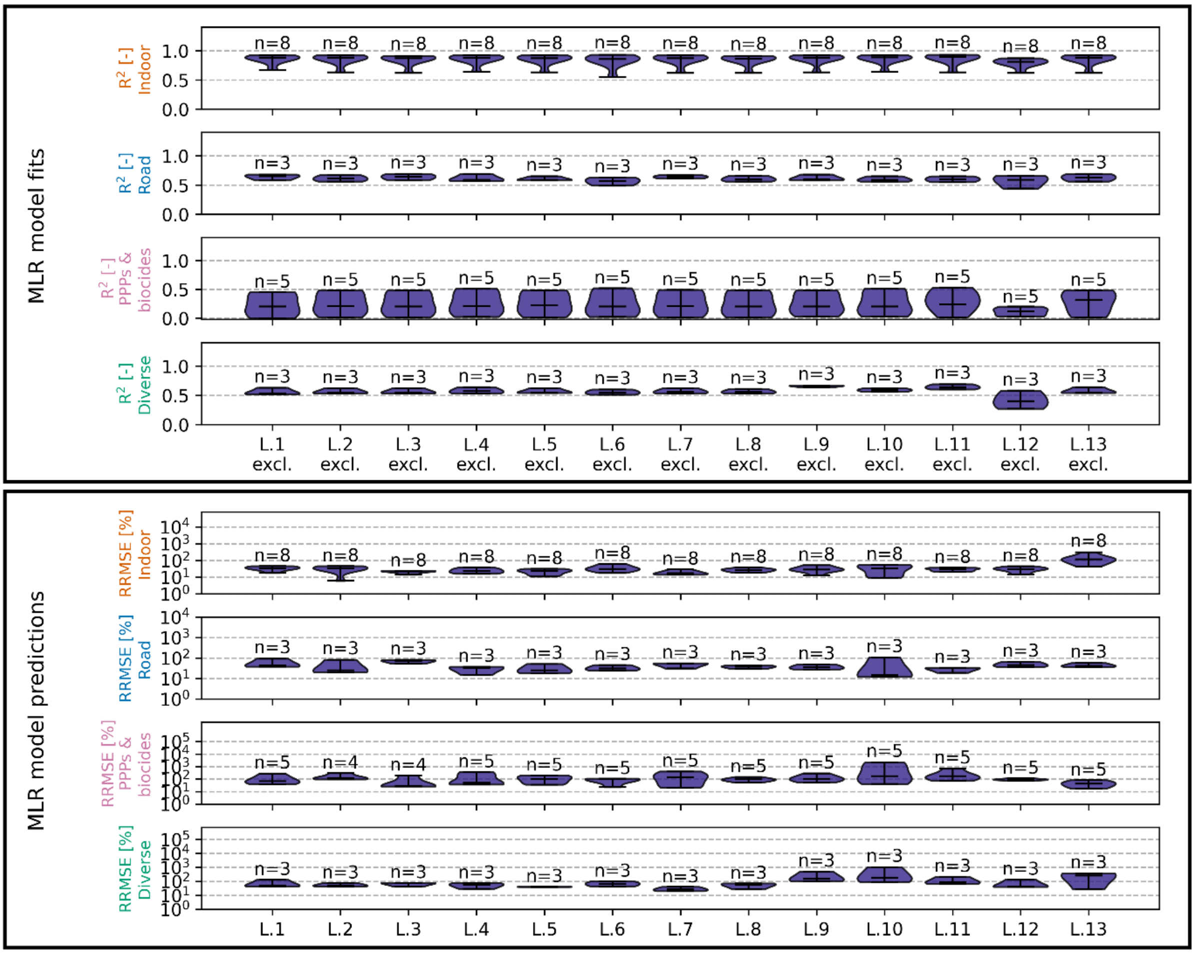

S1 Fig. 26. MLR model fits and predictions for catchment L. The violin plots in the upper panel display the R^2 of the model fits including all rain events except one in catchment L. The violin plots in the lower panel display the RRMSE of the model prediction for the excluded rain event. *n* indicates the number of substances > LOQ in every training set (upper panel)/testing set (lower panel). The horizontal line indicates the median. Table 4 in the manuscript shows which sensor was chosen for the MLR models of every chemical group. S3 Tables 20 show further statistical measures of the MLR models displayed here. Abbreviations: MLR: multiple linear regression, PPPs: plant protection products.

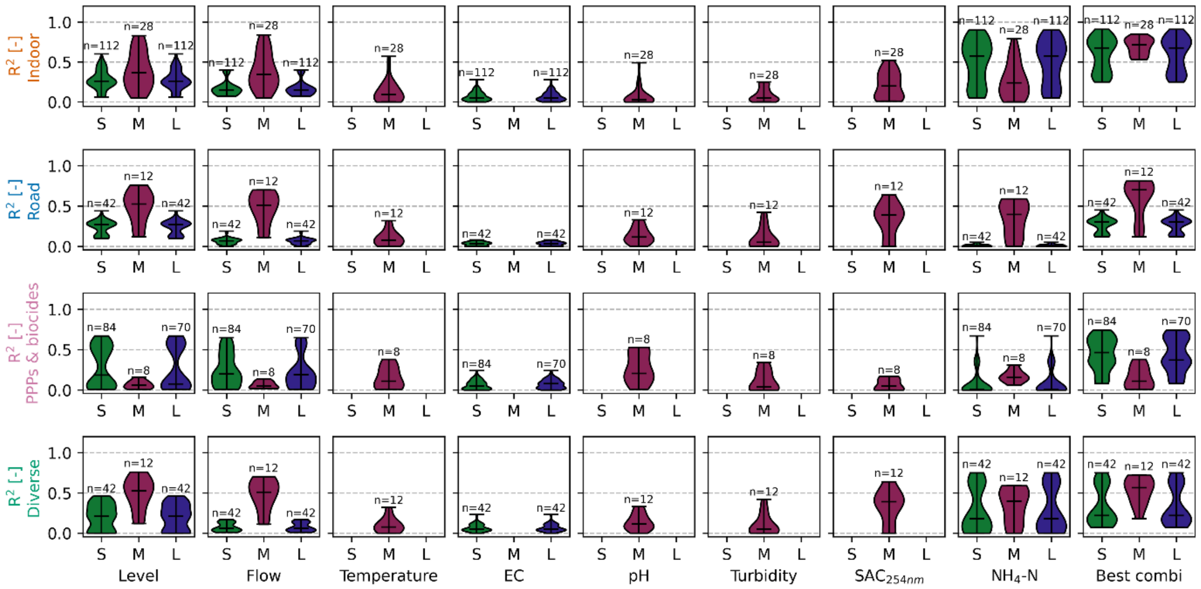

S1 Fig. 27. Goodness of fit of SRL models (sensors) and MRL models (best combi). The violin plots display the R^2 of the models fitted to chemical concentrations measured during all rain events, excluding one in catchments S, M, and L. The sensors are ordered accordingly to their complexity (costs, maintenance, and operation requirements). *n* indicates the number of model fits (substances*rain events) in every catchment. The horizontal line indicates the median. No boxplot is shown in case a sensor parameter was not measured in a catchment. S3 Tables 1-20 show further statistical measures of the linear regression models displayed here. Abbreviations: best combi: combination of sensors selected as predictors by the stepwise regression algorithm (manuscript Table 4), EC: electrical conductivity, NH_4-N: ammonium, $SAC_{254\ nm}$: spectral absorption coefficient at 254 nm, SLR: simple linear regression, PPPs: plant protection products.

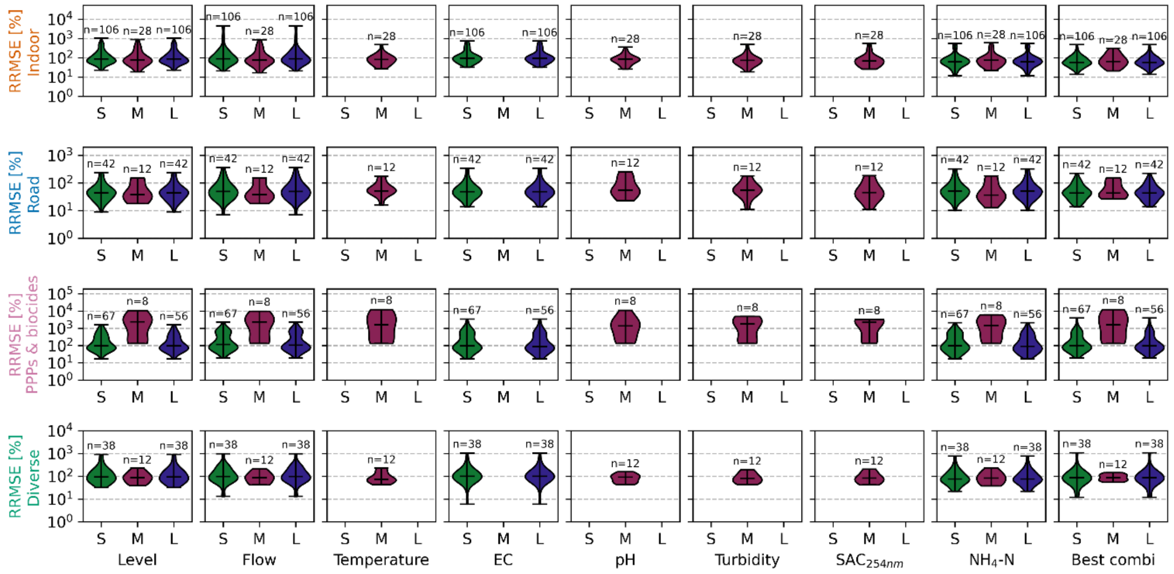

S1 Fig. 28. Error of prediction of SRL models (sensors) and MRL models (best combi). The violin plots display the RRMSE of the model predictions of chemical concentrations during one rain event in catchments S, M, and L. The sensors are ordered accordingly to their complexity (costs, maintenance, and operation requirements). *n* indicates the number of model predictions (substances*rain events) in every catchment. The horizontal line indicates the median. No boxplot is shown in case a sensor parameter was not measured in a catchment. S3 Tables 1-20 show further statistical measures of the linear regression models displayed here. Abbreviations: best combi: combination of sensors selected as predictors by the stepwise regression algorithm (manuscript Table 4), EC: electrical conductivity, MLR: multiple linear regression, NH_4-N: ammonium, $SAC_{254\,nm}$: spectral absorption coefficient at 254 nm, PPPs: plant protection products.

13. Random forest regression models for catchment L

Random forest regression (RFR) models were developed for all chemicals in catchment L to assess whether accounting for non-linear relationships improves the predictive performance compared to linear regression models. The data preprocessing followed the same procedure applied to the SLR and MLR models (manuscript section 2.5 Data analysis). Predictor variables were selected based on a feature importance threshold of 0.01 (S1 Table 14).

S1 Table 14. Sensors used by RFR models predicting every organic chemical in catchment L. ✖ indicates that a sensor was selected based on the feature importance threshold of 0.01. Abbreviations: EC: electrical conductivity, NH_4-N: ammonium, PPP: plant protection product, $SAC_{254\,nm}$: spectral absorption coefficient at 254 nm.

		Sensor parameter											
**Group**	**Chemical**	**Level**	**Dry level**	**Shifted level**	**Flow**	**Dry flow**	**Shifted flow**	**Temperature**	**EC**	**pH**	**Turbidity**	**$SAC_{254\,nm}$**	**NH_4-N**
Indoor	ACE				✖	✖	✖	✖	✖			✖	✖
	CAF				✖	✖	✖	✖	✖			✖	✖
	CYC				✖	✖	✖		✖	✖			✖
	CAN				✖	✖	✖		✖	✖		✖	✖
	CIT				✖	✖	✖	✖	✖	✖		✖	✖
	DCF				✖	✖	✖	✖	✖			✖	✖
	HCT				✖	✖	✖	✖	✖			✖	✖
	TCS				✖	✖	✖	✖	✖	✖		✖	✖
Road	DPG				✖	✖		✖	✖	✖		✖	✖
	6PPDQ				✖	✖		✖	✖	✖		✖	✖
	HMMM				✖	✖		✖	✖	✖		✖	✖
PPPs & biocides	2,4-D				✖	✖		✖	✖	✖		✖	✖
	DCMU				✖	✖		✖	✖	✖		✖	✖
	MCPA				✖	✖		✖	✖	✖		✖	✖
	MPP				✖	✖		✖	✖	✖		✖	✖
	OIT				✖	✖		✖	✖	✖		✖	✖
Diverse	MeBT				✖	✖		✖	✖	✖		✖	✖
	BT				✖	✖		✖	✖	✖		✖	✖
	DEET				✖	✖		✖	✖	✖		✖	✖

Performance comparisons indicate that RFR models did not outperform SLR or MLR models. Although their model fit was better for every chemical group (higher R^2), the predictive accuracy (RRMSE) was similar (S1 Fig. 29). These results suggest that overfitting is an issue for the RFR models. It is likely that the relationships between the sensor parameters and chemical concentrations in this dataset are predominantly linear, and more complex modelling approaches provide limited additional benefits.

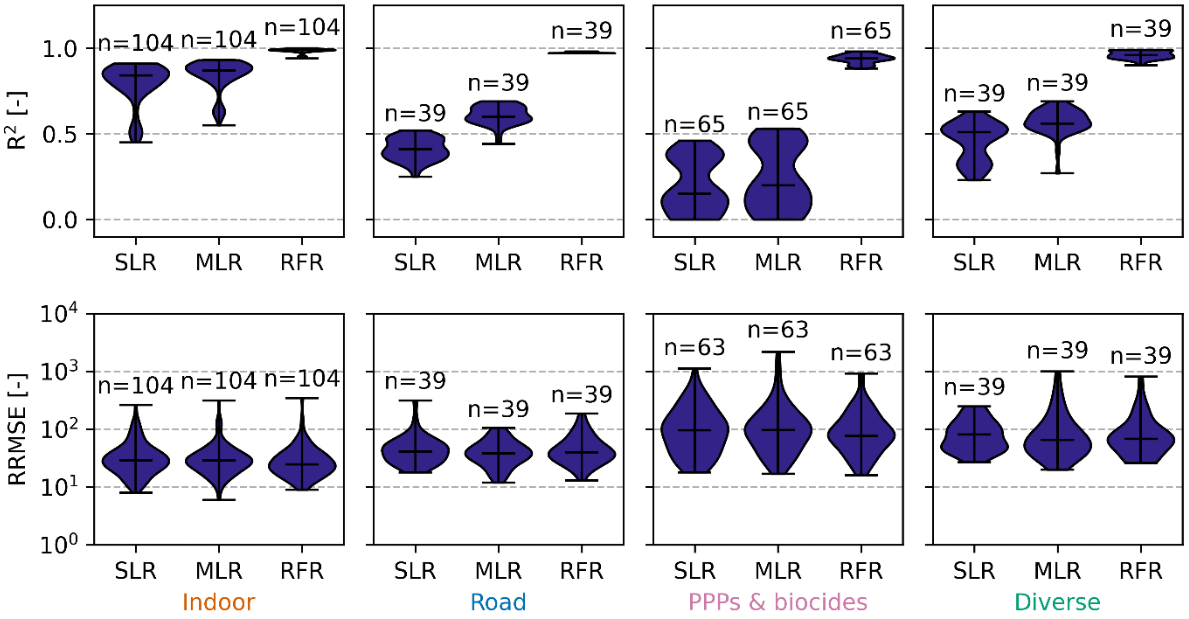

S1 Fig. 29. Comparison of SLR, MLR, and RFR model fits and predictions for organic chemicals in catchment L. The violin plots in the upper row show the R^2 of the SLR, MLR, and RFR models fitted to data from all rain events except one (training set). The violin plots in the lower row show the RRMSE of the SLR, MLR, and RFR model predictions for the excluded rain event (testing set). Table 4 of the manuscript (SLR and MLR models) and S1 Table 14 (RFR models) show which sensor parameters were used as predictors. The results of all chemicals of a chemical group (indoor, road, PPPs & biocides, and diverse) are agglomerated into one violin plot. *n* indicates the number of model fits (upper panel) and the number of model predictions (lower panel). The horizontal line indicates the median. S3 Table 21 shows further statistical measures of the RFR models displayed here. Abbreviations: SLR: single linear regression, MLR: multiple linear regression, RFR: random forest regression, PPP: plant protection products.

References

1. Lechevallier P, Gruber G, Bares V, Neuenhofer N, Waldner L, Mahajan A, et al. Dataset on wastewater quality monitoring with adsorption and reflectance spectroscopy in the UV/Vis range. 2024. doi:10.25678/000D3C.

2. Furrer V, Froemelt A, Singer H, Ort C. Data for: Source-specific dynamics of organic micropollutants in combined sewer overflows (Version 1.0). 2024. doi:10.25678/000DNY.

3. Royal Society of Chemistry. ChemSpider [Internet]. [cited 2023 Oct 27]. Available from: https://www.chemspider.com.

4. National Library of Medicine. PubChem [Internet]. [cited 2023 Oct 27]. Available from: https://pubchem.ncbi.nlm.nih.gov.

5. Furrer V, Mutzner L, Ort C, Singer H. Micropollutant concentration fluctuations in combined sewer overflows require short sampling intervals. Water Res X. 2023;21:100202. doi:10.1016/j.wroa.2023.100202.

6. Furrer V, Froemelt A, Singer H, Ort C. Source-specific dynamics of organic micropollutants in combined sewer overflows. Water Res. 2025;123416. doi:10.1016/j.watres.2025.123416.

7. Lechevallier P, Gruber G, Bares V, Neuenhofer N, Waldner L, Mahajan A, et al. Dataset on wastewater quality monitoring with adsorption and reflectance spectrometry in the UV-vis range. Sci Data. 2025;12:1296. doi:10.1038/s41597-025-05459-x.

8. Launay MA, Dittmer U, Steinmetz H. Organic micropollutants discharged by combined sewer overflows – Characterisation of pollutant sources and stormwater-related processes. Water Res. 2016;104:82–92. doi:10.1016/j.watres.2016.07.068.

9. Madoux-Humery AS, Dorner SM, Sauvé S, Aboulfadl K, Galarneau M, Servais P, et al. Temporal analysis of E. coli, TSS and wastewater micropollutant loads from combined sewer overflows: Implications for management. Environ Sci Process Impacts. 2015;17(5):965–74. doi:10.1039/c5em00093a.

10. linkage — SciPy v1.14.1 Manual [Internet]. [cited 2024 Dec 20]. Available from: https://docs.scipy.org/doc/scipy/reference/generated/scipy.cluster.hierarchy.linkage.html#scipy.cluster.hierarchy.linkage.

11. Hornung RW, Reed LD. Estimation of average concentration in the presence of nondetectable values. Appl Occup Environ Hyg. 1990 Jan 1;5(1):46–51. doi:10.1080/1047322X.1990.10389587.

12. Bertrand-Krajewski JL, Chebbo G, Saget A. Distribution of pollutant mass vs volume in stormwater discharges and the first flush phenomenon. Water Res. 1998;32(8):2341–56. doi:10.1016/S0043-1354(97)00420-X.

13. Thomas O, Thomas MF. Urban Wastewater. In: Thomas O, Burgess C, editors. UV-Visible Spectrophotometry of Water and Wastewater. 2nd ed. Elsevier B.V.; 2017. p. 281–315.
