# Supplementary material for: Exploring online sensor parameters as proxies for polar organic chemicals—An innovative approach for combined sewer overflow monitoring
Source: PLoS One. 2025 Oct 13;20(10):e0333173. doi: 10.1371/journal.pone.0333173 (PMC12517529; doi:10.1371/journal.pone.0333173)
Supplement: Attachment — Submitted filename: Editor Comments_v1.docx [file pone.0333173.s004.docx]

**Reviewer Comments#**
This manuscript presents original research evaluating the use of online sensor parameters (e.g., SAC254, NH₄-N, EC) as proxies for tracking polar organic chemicals in combined sewer overflows (CSOs) during wet weather. The authors use data from three urban catchments of varying sizes (S, M, L) to assess the predictive performance of these parameters, supported by regression modeling.

The study is timely and addresses a real-world monitoring gap. The experimental design is generally sound, the analyses are well-structured, and the conclusions are supported by the results.

However, I recommend minor revisions before publication to enhance clarity and reproducibility.

**Specific Comments:**

**Abstract>>**

- **Line 23**: It would be helpful to list what the traditional methods are when introducing online sensors.
- **Line 29**: Specifically, define "spectral absorption coefficient at 254 nm (SAC254 nm)".
- **Line 33**: The term "predict" may be too strong here. I recommend rephrasing to "prediction of concentrations of pesticides…".

**2 Materials and methods>>**

**Sampling Methodology (Lines 99–112)**

- How was the dry weather flow baseline defined for each catchment? Was it based on long-term averages, daily minimums, or another method?
- The dry weather baselines vary significantly between catchments (e.g., 288 m³/h in Catchment S vs. 5040 m³/h in Catchment L). Could the authors explain how these thresholds were selected, and if other factors like imperviousness or sewer size influenced this choice?
- Were samples collected using a time-based or flow-paced method? A brief explanation of the autosampler setup (e.g., sample interval, bottle volume, trigger mechanism) would clarify data consistency.
- Sampling for rain event M.2 started 50 minutes late. How did this delay affect the dataset and any subsequent analyses? Was this event treated differently in modeling?
- Two rain events in Catchment L (L.11 and L.12) included pre-rainfall samples, unlike others. Why was this done, and did it affect comparability across events?

**Polar Organic Chemicals (Lines 99–112)**

- How were the final 20 chemicals selected from the pool identified by Furrer et al. [24]? Were they ranked by frequency, concentration, or relevance?
- How did the authors determine the dominant source for each compound, especially for those like DEET or benzotriazole with multiple sources?
- Why was the LogKow ≤ 5 threshold chosen? Could this exclude slightly more hydrophobic compounds commonly found in CSOs?
- What criteria were used to classify compounds like DEET or benzotriazole as "diverse"? Was this based on data, modeling, or literature?
- Can the authors comment on the environmental or ecotoxicological significance of the selected chemicals for CSO impact assessment?

**Section 2.4: Online Sensors**

1. Why was NH₄-N only measured in the lab? Was there a reason online sensors weren’t used (e.g., cost, maintenance)?
2. Sensor availability varies across sites. How did this affect the comparability of proxy performance between catchments?
3. Please clarify how sensors were calibrated and maintained during field use.
4. Since turbidity and SAC254 were both available in M and L, was turbidity evaluated as a chemical proxy?
5. How were sensor readings aligned with sample collection times? Was any interpolation used?
6. Were data quality controls (e.g., filtering, outlier removal) applied to sensor data before analysis?
7. Can the authors briefly comment on the cost implications of deploying and maintaining these sensors in real-world settings?

**Reviewer Comments – Results and Discussion**

- The link between catchment size and dilution patterns is interesting. Can the authors comment on how this generalizes beyond the three sites?
- Was overfitting in catchment M assessed or mitigated in model development?
- Given negative predictions and high errors, why were only linear models used? Would log or nonlinear models help?
- Were long rain events weighted differently to avoid bias in model performance?
- Poor pesticide prediction is noted—could this be due to usage patterns, degradation, or sensor mismatch?
- How were incomplete events (e.g., M.2) handled in the modeling?
- Could larger datasets make machine learning models more viable in the future?
- Is a 30–40% error acceptable for practical CSO monitoring or mitigation decisions?

**Practical Suitability and Cost**

1. Can the authors comment on the practical feasibility of deploying and maintaining these sensors in typical municipal CSO sites (e.g., ease of access, vandalism risk, maintenance needs)?
2. What are the approximate costs (equipment + maintenance) per sensor type, and how might this scale across a network?
3. How much staff time or technical expertise is needed to operate these systems and interpret the data?
4. Are the proposed regression models robust enough for routine use by utilities without specialist data scientists?

**Conclusion**

- Why does catchment size impact indoor chemical behavior? Is there a size threshold affecting this?
- Why are SAC254 nm and NH₄-N more effective in larger catchments, and what challenges exist in smaller catchments?
- What other environmental factors could improve the prediction of road-runoff chemicals?
- Why do additional sensors not significantly improve predictions? Is it due to sensor limitations or chemical complexity?
- How can future studies capture complex chemical behaviors like those of PPPs and biocides?
- Could hybrid methods combining sensors and traditional monitoring improve accuracy for complex chemicals?

**References Section**

- Some references use different citation styles (e.g., references 3 and 18 don’t follow the journal article format properly).
- "Manasa RL, Mehta A. Environmental Biotechnology Vol. 2" is listed as a book chapter, but the journal article format isn't followed for the citation.
- Some references lack proper DOI formatting (e.g., references 26 and 27).
- References 23 and 24 appear to be from the same author (Launay et al.) but are slightly different in formatting and may be a duplicate of the same research.
- Reference 25 mentions a preprint ("OSF Prepr"), which should clearly indicate that it’s a preprint with proper notation.
- There are inconsistencies in punctuation, such as missing periods at the end of some references (e.g., reference 16).
