# Supplementary material for: Exploring online sensor parameters as proxies for polar organic chemicals—An innovative approach for combined sewer overflow monitoring
Source: PLoS One. 2025 Oct 13;20(10):e0333173. doi: 10.1371/journal.pone.0333173 (PMC12517529; doi:10.1371/journal.pone.0333173)
Supplement: Attachment — Submitted filename: Response_to_reviewers.docx [file pone.0333173.s005.docx]

# Response to reviewers

Title: Exploring online sensor parameters as proxies for polar organic chemicals – An innovative approach for combined sewer overflow monitoring

Laura Waldner, Viviane Furrer, Pierre Lechevallier, Fabienne Maire, Heinz Singer, Lena Mutzner

We thank the reviewers for their in-depth critical evaluation of our manuscript. This helped us to improve the manuscript for future readership. We have extended the manuscript by including more details on the methodology and context of the organic chemicals analyzed, as well as by adding a new section (3.5) on practical considerations for future application of the developed monitoring approach and further research needs, among other enhancements.

Please refer to our detailed point-by-point answers below (line numbers refer to the clean version of the manuscript). In addition to the suggested changes, we have made further revisions to the document to enhance the flow and key messages of the manuscript, and to comply with PLOS ONE’s style requirements fully.

## Reviewer 1

**Reviewer Comments**

This manuscript presents original research evaluating the use of online sensor parameters (e.g., SAC_254_, NH₄-N, EC) as proxies for tracking polar organic chemicals in combined sewer overflows (CSOs) during wet weather. The authors use data from three urban catchments of varying sizes (S, M, L) to assess the predictive performance of these parameters, supported by regression modeling.

The study is timely and addresses a real-world monitoring gap. The experimental design is generally sound, the analyses are well-structured, and the conclusions are supported by the results.

However, I recommend minor revisions before publication to enhance clarity and reproducibility.

Thank you for your detailed and positive assessment of our manuscript. We appreciate your acknowledgement of the study’s timeliness and methodological soundness. In response to your comments, we have carefully reviewed the manuscript and made several modifications to improve clarity and reproducibility, including:

- Clarifications in the methods section (e.g., sampling methodology, selection of organic chemicals, online sensor maintenance, and application)
- Clarifications on the results and discussion section (e.g., organic chemical dynamics, model settings)
- Introduction of a new section 3.5 “Practical considerations for future application of sensor-based monitoring and remaining research needs”
- Corrections of mistakes in the reference list

These changes are now reflected in the revised manuscript (see the manuscript with tracked changes and answers below, line numbers refer to the clean manuscript). We hope that these improvements address your comments and feel they further strengthen the manuscript.

**Specific comments:**

**Comments on abstract**

**1.1 Line 23**: It would be helpful to list what the traditional methods are when introducing online sensors.

We agree that this information was missing and have now added it to the abstract. Traditionally, grab or composite samples are taken manually or with an automated samplers at specific time intervals. The most commonly used methods for analysis of organic chemicals in wastewater are GC-MS and LC-MS. We added these methods to the text and changed “traditional” to “commonly used” for clarification.

Modifications in text, lines 23-25:

Currently, most overflow sites are not monitored because commonly used methods, such as automated grab sampling followed by laboratory analysis using liquid chromatography coupled with mass spectroscopy (LC-MS), are costly and time-consuming.

**1.2 Line 29**: Specifically, define "spectral absorption coefficient at 254 nm (SAC254 nm)".

Thanks for pointing out, we added the definition for SAC_254 nm_.

Modifications in text, lines 30-32:

In the largest catchment (160,000 inhabitants), indoor chemicals are strongly correlated with flow, electrical conductivity, the spectral absorption coefficient at 254 nm (SAC_254 nm_), and ammonium (NH_4_-N).

**1.3 Line 33**: The term "predict" may be too strong here. I recommend rephrasing to "prediction of concentrations of pesticides…".

We agree and have rephrased the sentence according to your suggestion.

Modifications in text, lines 36-37:

However, the prediction of pesticide concentrations remains limited, as these chemicals exhibit diverse patterns across rain events.

**Comments on 2. materials and methods**

**Comments on 2.2 sampling methodology (Lines 99–112)**

**1.4**: How was the dry weather flow baseline defined for each catchment? Was it based on long-term averages, daily minimums, or another method?

Thank you for highlighting the missing definition of the dry weather flow baseline. We added the information to the manuscript that in catchments S and L, sampling was not based on a dry weather baseline but was triggered when a CSO discharge was activated, at flow thresholds of 288 m³/h and 5,400 m³/h, respectively [1]. We also corrected a typo: the threshold for catchment L was previously listed incorrectly as 5,040 m³/h. In catchment M, the dry weather baseline was used to initiate sampling, as in this case, a bypass (rather than a CSO) was monitored. The baseline was defined as the average flow during dry weather days during the measurement campaign of Lechevallier et al. [2] (average of 235 days).

Modifications in text, lines 115-126:

In catchment S, combined sewage was sampled during 19 rain events by Furrer et al. [13,32]. The sampling of a rain event was initiated when the flow in the sewer exceeded the system capacity of 288 m^3^/h, indicating a CSO discharge. Sampling continued until the flow fell below 288 m3/h or until all bottles of the autosampler were full. In catchment M, four rain events were sampled by Lechevallier et al. [33]. As a bypass of the sewer was sampled, sampling started when the flow in the sewer increased above its dry weather baseline (300–380 m^3^/h, depending on the time of day), calculated from average dry weather flows. Sampling ended when it fell below the dry weather baseline or when all bottles of the autosampler were full. For rain event M.2, sampling started 50 minutes late due to problems with the autosampler. Despite the missing initial phase, rain event M.2 was retained for analysis due to the limited size of the dataset (comprising only four rain events). The potential impact of the delayed sampling start is discussed in section 3.4. In catchment L, 13 rain events were sampled. Sampling for all rain events except two was initiated when the flow in the sewer exceeded the system capacity of 5,400 m^3^/h [13,32].

**1.5**: The dry weather baselines vary significantly between catchments (e.g., 288 m³/h in Catchment S vs. 5040 m³/h in Catchment L). Could the authors explain how these thresholds were selected, and if other factors like imperviousness or sewer size influenced this choice?

See also response 1.4 for details on how the flow thresholds were defined (sewer capacity in catchments S and L, dry weather baseline in catchment M). The variation in flow thresholds reflects differences in catchment size, with a larger catchment size and more connected people in catchment L compared to catchment S. Additionally, the sewer infrastructure capacity, i.e., the volume that can be discharged to a wastewater treatment plant, varies. For example, catchment L covers a larger and more urban area and is serviced by a higher-capacity sewer system than catchment S, which explains the higher flow threshold. Additionally, thresholds for CSO activation are typically set by local utilities or municipalities based on hydraulic modeling and infrastructure design criteria, which may also account for differences between sites. We clarified this in the manuscript.

Modifications in text, lines 132-133:

The flow thresholds defined reflect differences in sewer system capacity and catchment characteristics, such as the number of connected people and the size of the connected impervious area.

**1.6**: Were samples collected using a time-based or flow-paced method? A brief explanation of the autosampler setup (e.g., sample interval, bottle volume, trigger mechanism) would clarify data consistency.

The sampling intervals and procedures are explained in the “Sampling procedure” section (lines 134-141). Samples were collected using either grab sampling or time-weighted composite sampling with automated samplers. Grab samples were taken every 10 minutes, while composite samples were created by combining five 2-minute subsamples to represent a 10-minute interval. Additionally, this section references S1 Table 2, which provides an overview of the sample type and number of samples collected during each rain event. The trigger mechanism for initiating sampling is explained in the previous paragraph (“Start of sampling”) and is based on flow thresholds specific to each catchment (288, 300-380, and 5,400 m^3^/h, see responses 1.4 and 1.5). We have added information on the bottle volume and sampling volume as suggested.

Modifications in text, lines 134-141:

Samples for analysis of polar organic chemicals were collected either as grab samples or as time-weighted composite samples in 1 L glass bottles using automated samplers (TP5 C, MAXX Mess- und Probenahmetechnik GmbH). Grab samples (250 mL) were taken every 10 minutes. For composite sampling, subsamples were taken every 2 minutes, 150 mL in catchments S and L, and 50 mL in catchment M. Five subsamples were combined to a composite sample (750 mL or 250 mL) representing 10 minutes. S1 Table 2 provides an overview of the sample types and the number of samples collected during each rain event. Further details on the sampling procedure can be found in Furrer et al. [13,32] and Lechevallier et al. [33].

**1.7**: Sampling for rain event M.2 started 50 minutes late. How did this delay affect the dataset and any subsequent analyses? Was this event treated differently in modeling?

Sampling for rain event M.2 started 50 minutes late due to a technical issue with the autosampler. As a result, the initial phase of the rain event, including peak runoff, was not captured. As noted in the limitation section (lines 454-458), the missing start of M.2 weakens correlations and model predictions (S1 Figs. 16, 23, and 24). This indicates that full event dynamics are crucial for accurately interpreting chemical transport patterns. However, as reliable interpolation was not possible (see response 2.6) and the dataset for catchment M was already small (only 4 rain events), we decided to retain event M.2 for the analyses.

Modifications in text, lines 122-125:

For rain event M.2, sampling started 50 minutes late due to problems with the autosampler. Despite the missing initial phase, rain event M.2 was retained for analysis due to the limited size of the dataset (comprising only four rain events). The potential impact of the delayed sampling start is discussed in section 3.4.

**1.8**: Two rain events in Catchment L (L.11 and L.12) included pre-rainfall samples, unlike others. Why was this done, and did it affect comparability across events?

Furrer et al. [1,3] originally included a 1-hour dry weather period before two rain events in catchment L to investigate whether including pre-event conditions would enhance correlations between indoor chemicals and other parameters (e.g., NH_4_-N, DOC) due to stronger dilution effects.

However, they did not observe a significant improvement in correlations, and we similarly found no impact on model performance when including these pre-rainfall samples (previously discussed in section 3.4). In response to your comment, we decided to exclude these samples from our analysis to ensure consistency across all events.

Modifications in text, lines 125-132:

In catchment L, 13 rain events were sampled. Sampling for all rain events except two was initiated when the flow in the sewer exceeded the system capacity of 5,400 m³/h [13,32]. However, sampling for rain events L.11 and L.12 originally included a one-hour dry weather period before the onset of rainfall [32]. Furrer et al. [32] sampled pre-rainfall to investigate whether pre-event dilution dynamics would enhance correlations between indoor-sourced chemicals and additional parameters (e.g., NH_4_-N, dissolved organic carbon). However, as no significant improvement in correlation was found [32], we excluded these pre-rainfall samples from our analysis to ensure consistency across all events.

**Comments on 2.3 polar organic chemicals (Lines 99–112)**

**1.9**: How were the final 20 chemicals selected from the pool identified by Furrer et al. [24]? Were they ranked by frequency, concentration, or relevance?

The 20 chemicals measured in Lechevallier et al. [2] were selected based on the findings of Furrer et al. [1,3] and the following selection criteria: (i) high polarity, (ii) frequent detection and elevated concentrations in urban runoff and wastewater, (iii) representation of a broad range of urban sources (e.g., household, traffic, pesticides), and (iv) the expectation of source-specific temporal dynamics. In this study, we only included the 20 chemicals that were measured by both studies/in all three catchments.

Modification in text, lines 144-149:

The selection criteria included polarity, high occurrence and concentration, representation of various urban sources, and expected source-specific behavior. Among the selected chemicals, triclosan, 1,3-diphenylguanidine, diuron, and mecoprop have been identified as major contributors to ecotoxicological risk in CSO discharges [3]. The selected compounds were included in both datasets reported by Furrer et al. [35] and Lechevallier et al. [33]. Only Carbendazim data is missing for catchment L.

**1.10**: How did the authors determine the dominant source for each compound, especially for those like DEET or benzotriazole with multiple sources?

The dominant sources for each compound were determined based on an extensive literature review, considering typical usage, emission pathways, and previous findings on the occurrence of these substances in urban environments. Compounds were assigned to the categories “indoor”, “road runoff”, and “plant protection products & biocides”. When a compound was associated with multiple sources (e.g., DEET or benzotriazole), it was assigned to the category “diverse.” This classification acknowledges the complexity of urban chemical emissions and reflects that several sources can contribute rather than a single dominant one. We added an explanation of this classification to the manuscript.

Modification in text, lines 155-157:

Compounds were assigned to the categories “indoor”, “road runoff”, and “plant protection products & biocides” based on a literature review of their typical uses. Substances with multiple contributing sources were categorized as “diverse” to reflect their mixed origins.

**1.11**: Why was the LogKow ≤ 5 threshold chosen? Could this exclude slightly more hydrophobic compounds commonly found in CSOs?

The LogK_OW_ ≤ 5 threshold was selected because the focus of this study was on polar, hydrophilic (i.e., dissolved) organic compounds, which are less well understood in the context of CSOs, as highlighted in the introduction (lines 81-82). By definition, polar chemicals are not hydrophobic and typically exhibit lower LogK_OW_ values. LogK_OW_ ≤ 5 was chosen as a threshold for polarity according to Zessner et al. [4]. This threshold reflects our intention to specifically investigate the behavior of more mobile, water-soluble substances rather than particle-bound or hydrophobic compounds.

Modification in text, lines 152-154:

Polar substances were selected because they are understudied in the context of CSOs. Following Zessner et al. [36], a threshold of LogK_ow_ ⩽ 5 was chosen for polarity to ensure dissolution and transport in the water phase.

**1.12**: What criteria were used to classify compounds like DEET or benzotriazole as "diverse"? Was this based on data, modeling, or literature?

As outlined in our response 1.10, compounds were classified as “diverse” based on literature sources when no dominant emission pathway could be identified due to multiple relevant uses. For example, DEET is used as both an insect repellent and an insecticide, while benzotriazole is present in various household and industrial products.

**1.13**: Can the authors comment on the environmental or ecotoxicological significance of the selected chemicals for CSO impact assessment?

The ecotoxicological relevance of the selected chemicals is discussed in detail by Furrer et al. [5]. Their study evaluated their risk contributions in CSOs from catchments S and L [5]. As shown in their Fig. 2, chemicals from the categories pharmaceuticals (referred to as indoor chemicals in our study), road chemicals, and pesticides contribute significantly to ecotoxicological risk. Among the 20 chemicals analyzed in our study, triclosan, 1,3-diphenylguanidine, diuron, and mecoprop are particularly relevant due to their high toxicity [5]. Nonetheless, the remaining substances are also important, given their frequent occurrence and persistence in urban stormwater systems.

Modifications in text, lines 146-147:

Among the selected chemicals, triclosan, 1,3-diphenylguanidine, diuron, and mecoprop have been identified as major contributors to ecotoxicological risk in CSO discharges [3].

**Comments on 2.4 online sensors**

**1.14**: Why was NH₄-N only measured in the lab? Was there a reason online sensors weren’t used (e.g., cost, maintenance)?

Lechevallier et al. [2] measured NH_4_-N both with an ISEmax online sensor and through laboratory analysis for samples collected in catchment M. For this study, we relied on laboratory measurements because the sensor data showed poor correlation with the reference laboratory values (see Table 4 in Lechevallier et al.) [2]). Despite several efforts to identify and correct potential issues, such as a possible time delay, lack of automated cleaning, or interference from industrial discharge, the sensor data remained inconsistent, particularly showing an unusual and unexplained nighttime peak (see Fig. 10 in Lechevallier et al. [2]). ). Given these reliability concerns, we chose to use the more accurate laboratory measurements for this analysis. In catchments S and L, Furrer et al. [1,3] measured NH_4_-N only in the laboratory, as no online sensor was available at the time of sampling.

Modifications in text, lines 174-176:

Although NH_4_-N can theoretically be measured with an ion-selective electrode sensor, this study used laboratory values because no sensor data were available for catchments S and L, and sensor measurements in catchment M proved unreliable [33].

**1.15**: Sensor availability varies across sites. How did this affect the comparability of proxy performance between catchments?

Sensor availability varied between catchments, but this had a limited impact on the overall comparability of proxy performance. Water level data were only available in catchments S and M, but flow data were available in all three catchments. Since water level and flow were strongly correlated (see Fig. 2, S1 Fig. 11), information on hydraulic dynamics was available in all catchments through flow data. Temperature, pH, and turbidity were missing in catchment S and/or L, but this did not affect the outcome, as these parameters consistently showed weak correlations with chemical concentrations.

The main limitation is the absence of EC and SAC_254 nm_ data in catchment S. Both are strong proxies for indoor chemicals, so their absence limits our ability to assess the influence of catchment size on their performance. However, findings from catchments M and L suggest that EC and SAC_254 nm_ become more reliable proxies with increasing catchment size (Fig. 2), likely due to more consistent and pronounced dilution patterns of indoor sources. This trend is similar to the one observed for NH_4_-N, another effective proxy for indoor chemicals. These limitations are now discussed in section 3.4 Limitations.

Modifications in text, lines 459-468:

Another limitation is the unequal availability of sensor data across catchments. While water flow data were available everywhere, water level was only measured in catchments S and M. However, due to the strong correlation between flow and level, information on hydraulic dynamics was available for all catchments. Temperature, pH, and turbidity sensors were missing in catchments S and/or L. However, this had a minimal impact on the outcomes of this study due to the generally low correlations between these parameters and chemical concentrations. The absence of EC and SAC_254 nm_ in catchment S is more critical. As these parameters are reliable proxies for indoor chemicals, their catchment-size dependence could not be fully assessed. However, findings from catchments S and M suggest that EC and SAC_254 nm_ become more reliable proxies with increasing catchment size, as is the case for NH_4_-N, which was available in all three catchments (Fig. 2).

**1.16**: Please clarify how sensors were calibrated and maintained during field use.

The maintenance of each sensor is specified in S1 Table 7. This table has been updated to include an additional column that clarifies the calibration of each sensor. The main manuscript now refers more clearly to this table, which ensures transparency regarding sensor handling during field deployment.

Modifications in text, lines 176-178:

S1 Table 7 provides an overview of the sensor models, measurement intervals, and locations, as well as the maintenance and calibration procedures for all sensors used in this study.

**1.17**: Since turbidity and SAC_254_ were both available in M and L, was turbidity evaluated as a chemical proxy?

Yes, turbidity was evaluated as a proxy using the same approach applied to all other sensor parameters, as shown in Fig. 2. However, since its correlations with all chemical groups were consistently low (Pearson correlation coefficients between 0.20 and 0.22), it is not a reliable proxy for any of the chemical groups. Hence, we did not discuss it in detail in the manuscript.

**1.18**: How were sensor readings aligned with sample collection times? Was any interpolation used?

Sensor readings were aligned with sample collection times as described in the methods section “Pre-treatment of sensor data”. For grab samples, sensor values at the exact sampling time were used. For composite samples, sensor readings at the five subsample time points were averaged to represent each 10-minute interval. In cases where sensor readings did not align precisely with sampling times, linear interpolation was applied. We realized this interpolation step was not clearly stated in the manuscript and have now added it.

Modifications in text, lines 196-199:

Sensor data were preprocessed to match the sampling time points. For grab samples, only sensor values at the exact sampling time were retained; for composite samples, the mean of sensor values measured at the five subsample time points was calculated. If sensor readings did not align with the (sub-)sampling times, sensor values were linearly interpolated.

**1.17**: Were data quality controls (e.g., filtering, outlier removal) applied to sensor data before analysis?

We did not apply additional quality controls to the data published on the ERIC/open data repositories [6,7]. However, Lechevallier [2] thoroughly explains the methods for preprocessing the data collected in catchment M. In brief, data pre-processing included timestamp harmonization and outlier removal by defining, for each sensor, a validity range outside of which data are considered invalid. Data validation was performed quantitatively by calculating the correlation between two independent data sources, such as laboratory measurements and sensor readings, or between two different sensors. They found high data reliability for all sensors except the ion-selective electrode, which was not used in this study, as explained in response 1.14.

In contrast, Furrer et al. [1,3] did not apply specific preprocessing procedures for the sensor data from catchments S and L, except for timestamp harmonization. To ensure consistency across datasets, we applied a minimal and transparent approach: single outliers were identified and replaced using linear interpolation (as described in the methods section and S1 Section 5). No additional filtering or smoothing was performed to minimize the introduction of subjective bias.

Modifications in text, lines 189-195:

Lechevallier et al. [33] describe the data preprocessing procedures applied to sensor data from catchment M in detail. These included timestamp harmonization, outlier removal based on sensor-specific validity ranges, and quantitative quality checks. In contrast, Furrer et al. [13,32] did not apply specific preprocessing steps to the sensor data from catchments S and L, except for timestamp harmonization. Therefore, this study identified and replaced single outliers using linear interpolation (see S1 Section 5 for details). No additional filtering or smoothing was applied to data from catchments S and L to avoid introducing subjective bias.

**1.18**: Can the authors briefly comment on the cost implications of deploying and maintaining these sensors in real-world settings?

We have introduced a new Section 3.5 to discuss practical considerations for implementing our sensor-based monitoring approach, including associated costs. The cost of deploying and maintaining sensors in real-world settings varies depending on the type of sensor and the installation context, as shown in S1 Table 8. Basic parameters such as water level, flow, and temperature can be monitored relatively affordably with low-maintenance sensors. In contrast, more advanced sensors such as UV-Vis spectrophotometers or ion-selective electrodes are more expensive. Their price typically ranges from 1,000 to 30,000 euros. Additionally, optical sensors require more frequent cleaning and calibration, which increases maintenance effort and cost (S1 Table 7).

However, compared to traditional monitoring approaches such as automated sampling followed by laboratory analysis using LC-MS, sensors offer significant cost advantages. LC-MS instruments are substantially more expensive (approx. 300’000-400’000 euros for a triple quadrupole MS). Additionally, automated sampling and analysis are very time-consuming and labor-intensive, and require highly trained staff. For example, private laboratories can charge up to 1’000 euros for analyzing organic chemicals in a single sample. As the sensor-based approach also requires chemical analysis for calibration, its initial phase is also expensive. However, after calibration, the cost per datapoint reduces quickly. Hence, for long-term monitoring, the sensor-based approach is more cost-efficient. For example, in our study, each rain event includes an average of 18 samples. If chemical analysis through a contract laboratory costs approximately €1,000 per sample, this results in €18,000 per event. In comparison, an ion-selective electrode (ISE) sensor costs around €4,000–6,000. Therefore, after the initial calibration phase, deploying an ISE sensor for monitoring indoor chemicals can offset its investment cost within a single rain event, making it a cost-effective solution for long-term monitoring. Additionally, online sensors enable continuous, real-time data collection, providing higher temporal resolution and faster insights, which can be crucial for understanding pollutant dynamics and making informed decisions quickly. Despite the initial investment, sensors may therefore reduce long-term monitoring costs and improve data coverage in urban water systems. We added these explanations to the new section 3.5 and added tables showing the approximate costs of the two approaches in S1 File (S1 Tables 8 and 9).

Modifications in text, lines 553-566:

As previously discussed, calibrating the sensor-based organic chemical prediction model requires chemical analysis of 4 to 13 rain events, each with an average of 18 samples. The calibration phase is relatively costly as it involves both sensor measurements and chemical analysis (see S1 Tables 8 and 9). However, once the model is calibrated, the return on investment is rapid due to the high per-sample cost of laboratory-based LC-MS analysis (S1 Table 9). Based on the cost estimates in S1 Tables 8 and 9, the investment costs in sensors can be offset after monitoring a single rain event, even when using more expensive sensors such as UV-Vis probes or ion-selective electrodes. Maintenance costs are expected to be similar to or lower than those of the traditional autosampler-based approach, depending on the number and type of sensors. The traditional approach also involves recurring costs, as site visits are necessary for sample collection after every rain event. Beyond cost savings, sensor-based monitoring offers additional advantages, including high temporal resolution, continuous measurements, and real-time data availability, features that are particularly valuable in highly dynamic environments, such as urban drainage systems. In summary, the sensor-based approach enables substantial cost reduction for long-term experimental campaigns while maintaining satisfactory accuracy.

**Comments on 3. Results and discussion**

**1.19**: The link between catchment size and dilution patterns is interesting. Can the authors comment on how this generalizes beyond the three sites?

For indoor chemicals, the observed link between catchment size and dilution patterns is likely generalizable to other sites. As discussed in detail in other studies [1,8] indoor chemicals exhibit more fluctuating temporal patterns in CSOs in small catchments due to the strong influence of individual pulse emissions, such as those from toilet flushes, dishwashers, and water taps. In larger catchments, these pulses disperse more along longer flow paths, and considerably more point sources exist and overlap, resulting in more pronounced dilution patterns [1]. While our study includes only three catchments, the consistency of this trend, along with its clear mechanistic explanation, suggests that it applies more broadly. However, we cannot define a specific minimum catchment size for observing source-specific dilution patterns. Our data suggest it likely lies between 2,000 and 20,000 inhabitants for most of the studied indoor chemicals, but likely depends on the frequency of a chemical’s use.

Modifications in text, lines 253-258:

Based on our data, this threshold appears to lie between 2,000 and 20,000 inhabitants, but it likely depends on the frequency of a chemical’s use. The observed relationship between catchment size and dilution patterns of indoor chemicals aligns with the mechanistic understanding that larger catchments smooth out individual pulses due to a large number of pulses and dispersion effects, suggesting that this trend is generalizable to other urban catchments.

**1.20**: Was overfitting in catchment M assessed or mitigated in model development?

Yes, potential overfitting in catchment M was assessed through leave-one-event-out cross-validation, which helps identify differences between training and test performance. In catchment M, the R^2^ of the training sets was often higher than the R^2^ of the test sets (S3 Table 2), indicating that overfitting was an issue due to the small size of the dataset (4 rain events). However, overfitting could not be mitigated as it was out of the scope of the study to collect more organic chemical data. Instead, we report that SLR models perform better than MLR models because overfitting becomes more severe when additional model parameters are introduced (S3 Table 19). We have clarified the issue of potential overfitting more explicitly in the manuscript and emphasized the uncertainty associated with model results in catchment M.

Modification in text, lines 365-370:

In catchment M, model fits are generally poor (median R^2^ = 0.37, Fig. 3a, S1 Fig. 19) as correlations with level are weak (PCC = -0.23, Fig. 2a). Additionally, overfitting to the training set is an issue (S3 Table 2) due to the small size of the dataset, making model results in catchment M uncertain.

**1.21:** Given negative predictions and high errors, why were only linear models used? Would log or nonlinear models help?

We considered log-transforming the response variable, as chemical concentrations often follow a log-normal distribution. While this transformation can stabilize variance and improve model fit for lower concentrations, it shifts the model toward minimizing relative errors. Since high concentrations are the most critical from an ecotoxicological perspective, we prioritize minimizing absolute errors in that range, and thus decided against log-transforming the response variable. Additionally, linear regression models predict the expected mean of the response variable given the predictors. Log-transformed models predict the geometric instead of the arithmetic mean.

We also tested more complex nonlinear models, such as random forest regression (see S1 Fig. 29 for catchment L). However, these models did not substantially improve predictive performance compared to linear regression. Given the minimal performance gain and the greater interpretability of linear models, we decided on the simpler approach.

We refined a paragraph in section 3.4 limitations to better reflect the rationale behind our choice of linear models.

Modifications in text, lines 476-488:

Additionally, models are fitted to absolute concentrations of polar organic chemicals and sensor parameters. Chemical concentrations typically follow a log-normal distribution, meaning that high values disproportionately influence model performance. While log-transforming the response variable can stabilize variance and improve the model fit for lower concentrations, it shifts the model’s focus away from high concentrations, which are most relevant from an ecotoxicological perspective. Therefore, we fitted concentrations on the original scale to capture absolute errors at the upper end of the range. Furthermore, linear regression can occasionally predict negative concentrations, particularly when extrapolating beyond the training range, and is unable to capture non-linear relationships between organic chemicals and sensor parameters. To explore the importance of non-linear relationships, we tested random forest regression models (see S1 Fig. 29), but found no substantial improvement in prediction accuracy. Given similar performance and the limited size of the dataset, which was insufficient to thoroughly test machine learning approaches, we selected linear regression as a robust and transparent baseline method.

**1.22**: Were long rain events weighted differently to avoid bias in model performance?

No, long rain events were not weighted differently in our analysis. Since every data point was weighted equally, longer events contributed more observations and therefore had greater influence on the model fit. We chose this model setting because we consider all individual measurements equally reliable and wanted to capture the full variability of organic chemical dynamics over time. We explained the decision of this model setting in section 3.4 limitations.

Modifications in text, lines 446-449:

Different numbers of samples were taken per rain event, depending on its length, and every data point was weighted equally based on our assumption that all individual measurements are equally reliable. Hence, longer rain events have a greater impact on the results than events with fewer data points.

**1.23**: Poor pesticide prediction is noted—could this be due to usage patterns, degradation, or sensor mismatch?

The poor prediction performance for pesticides is most likely due to their highly variable usage patterns and urban sources, resulting in irregular and hardly predictable concentration dynamics in combined sewage. Similar findings have been reported in other studies, where pesticide concentrations showed strong temporal fluctuations in stormwater [3,9,10]. Because these chemicals show no source-specific behavior (section 3.1) and no sensor proxy effectively reflects these strong fluctuations (section 3.2), linear models, which rely on linear combinations of sensor inputs, are inherently limited in capturing the variability of pesticides. Degradation is unlikely to be a major factor in the poor model performance. While degradation can reduce concentrations, it typically does not substantially alter temporal patterns over short timescales, especially for the relatively stable pesticides studied here. Additionally, samples were collected within hours after rainfall and stored at -20°C to minimize degradation after sampling. A mismatch between sensor parameters and pesticides is also unlikely, as the sensor values were used successfully to predict other chemical groups in the same samples.

To enhance clarity, we have now added a brief explanation in section 3.3 “Linear models cannot predict PPPs and biocides” that explicitly links the fluctuating pesticide concentrations (discussed in section 3.1) and the lack of suitable sensor proxies (discussed in section 3.2) to the challenges faced by linear models in predicting PPPs and biocides.

Modification in text, lines 425-430:

The poor model performance can be attributed to the lack of source-specific behavior of this chemical group and the fact that the used sensor proxies do not accurately reflect the strongly fluctuating and variable concentration patterns of any of the PPPs and biocides studied (S1 Figs. 11, 19, and 20). Linear models, based on linear combinations of sensor inputs, cannot capture such irregular temporal dynamics. Thus, the sensor-based models developed in this study are unable to predict PPP and biocide concentrations accurately.

**1.24**: How were incomplete events (e.g., M.2) handled in the modeling?

Event M.2 was the only incomplete rain event, as the beginning of sampling was missing. It was treated the same as all other events in the modeling because only four events were sampled in catchment M, and excluding M.2 would have significantly reduced the already limited dataset. We further specified this in the methods section (see response 1.7) and discussed its implications in the limitations section.

Modification in text, lines 454-458:

In addition, to capture the full dynamics, the beginning of the rain event needs to be sampled. The missing start of the rain event M.2 likely weakens correlations and model predictions (S1 Figs. 16, 23, and 24). Nevertheless, M.2 was retained for analysis, as only four rain events were available in catchment M, and excluding it would have further limited the already small dataset. This suggests that sampling the beginning of a rain event is crucial, as was already recommended by Furrer et al. [32].

**1.25**: Could larger datasets make machine learning models more viable in the future?

We are not experts in machine learning, but we believe that machine learning models could offer advantages over linear models if sufficiently large and diverse datasets were available. In this study, the dataset was too small, in terms of the number of events, samples, and chemicals, for machine learning approaches to be effective. We tested random forest regression models for catchment L, but they did not improve predictions while overfitting the training data (see response 1.21) .

Small datasets are a common limitation in studies involving organic micropollutants, as their analysis is very cost- and time-intensive, typically yielding only a few dozen to hundreds of data points. However, machine learning algorithms often require thousands of data points to capture complex, non-linear patterns and avoid overfitting reliably. Given these constraints, it is unlikely that such large datasets will be available in the near future unless major advances are made in the efficiency and affordability of organic chemical sampling and analysis.

**1.26**: Is a 30–40% error acceptable for practical CSO monitoring or mitigation decisions?

Considering the highly dynamic nature of organic chemicals in urban drainage systems, an error margin of 30–50% seems acceptable for practical monitoring of CSOs. CSOs exhibit significant variations both between and within events, which are further compounded by field and analytical uncertainties. For context, RRMSEs in the range of 10% to 20% are considered satisfactory for sensor measurements [11]. Furrer et al. [1] reported an uncertainty of 10% for laboratory analyses of organic chemicals. Hence, we believe that the linear regression models perform satisfactorily; however, the required accuracy depends on the intended application.

Modifications in text, lines 502-516:

For practical applications, it is crucial to determine the acceptable level of prediction accuracy. We consider error margins of 30% to 50% acceptable for monitoring organic chemicals in CSO discharges, given the highly dynamic conditions [13,15], as well as the inherent uncertainties associated with sensor measurements and sample processing. For context, RRMSEs of 10% to 20% are typically considered satisfactory for UV-Vis measurements in urban drainage systems [49], and a 10% error is common for chemical sampling and analysis [13]. In contrast, Hubeaux et al. [27] demonstrated that micropollutant abatement in wastewater treatment plants can be predicted with errors as low as 2% based on SAC_254 nm_ measurements. However, their study relied on 24-hour composite samples and did not address the short-term fluctuations typical of sewer systems. While very high precision may be achievable in relatively stable WWTP conditions, higher error margins need to be accepted in sewer monitoring due to greater variations in the concentrations of organic chemicals. Hence, this study suggests that the accuracy of sensor-based linear regression models is satisfactory for monitoring indoor chemicals in highly dynamic sewer systems of larger catchments and chemicals from road runoff. However, whether the model accuracy is satisfactory ultimately depends on the requirements of the intended application.

**Comments on practical suitability and cost**

**1.27**: Can the authors comment on the practical feasibility of deploying and maintaining these sensors in typical municipal CSO sites (e.g., ease of access, vandalism risk, maintenance needs)?

Deploying and maintaining sensors in urban drainage systems is challenging. The ease of access can be a further limiting factor, as many CSO structures are underground and not easily reachable, complicating installation and servicing. In many locations, the risk of vandalism is low due to the limited access to these structures compared to overground measurement sites. Despite these limitations, online sensors are more cost-efficient than traditional grab sampling followed by laboratory LC-MS analysis in the long term and can provide continuous and real-time data. We discuss these challenges and requirements in the new section 3.5 on practical considerations.

Modifications in text, lines 533-551:

Sensor-based monitoring is only effective if the sensors provide reliable, high-quality data. However, the environmental conditions in urban drainage systems are harsh, including variable flow regimes, corrosive gases, high humidity, and solid loads, which can impair sensor performance and data quality [22,52,53]. Frequent maintenance, including sensor cleaning and recalibration, is therefore essential to prevent sensor drift and biofilm formation, thereby ensuring the long-term stability of data and model performance [33]. The quality of the data used in this study was ensured through regular sensor maintenance (see S1 Table 7), which requires trained staff. For example, flow sensors were cleaned one to four times per year, while UV-Vis probes were cleaned on a weekly or biweekly basis (S1 Table 7). Where feasible, automated sensor cleaning with compressed air was used to further enhance sensor performance [33]. Lechevallier et al. [33] also demonstrated the value of automated quality control, including outlier detection, drift analysis, and weekly data inspection. However, further research is needed to evaluate the impact of reduced maintenance on sensor performance and model stability over longer periods. Additionally, access to underground urban drainage structures is often difficult, making it challenging to install and maintain sensors. For example, Furrer et al. [13] selected monitoring sites at the inlets of WWTPs in catchments S and L based on their good accessibility, which facilitated sensor installation and maintenance. In summary, regular maintenance, accessible monitoring sites, and trained personnel are essential for the successful implementation of the sensor-based monitoring approach developed in this study.

**1.28**: What are the approximate costs (equipment + maintenance) per sensor type, and how might this scale across a network?

We added S1 Tables 8 and 9, which provide an overview of equipment and operational costs for sensor-based monitoring and traditional methods (grab sampling + LC-MS analysis). These tables demonstrate that sensor-based monitoring is generally more cost- and time-efficient than traditional approaches, especially when long-term continuous and real-time data collection is desired. Both methods scale similarly across a sewer network when multiple monitoring stations are deployed. These cost comparisons and references to these tables have been included in the new section 3.5, see text modification in response 1.18.

**1.29**: How much staff time or technical expertise is needed to operate these systems and interpret the data?

Implementing and maintaining sensors in urban drainage systems is challenging and requires trained staff with technical expertise, as explained in response 1.27 and discussed in section 3.5.

Additionally, we added S1 Tables 8 and 9, which summarize the costs and time efforts of both approaches (sensors vs. sampling + LC-MS). Based on our experience, sensor calibration requires approximately 30 minutes per sensor per calibration interval (S1 Table 7), while routine maintenance takes around 15 minutes per sensor per maintenance interval (S1 Table 7), plus additional time for travel to the site. Data pipeline development requires expertise in data science. Once it is established, data loading and interpretation can typically be performed in approximately one hour per week. In comparison, sample preparation for organic chemical analysis typically takes around 15 minutes per sample and requires specialized laboratory skills. LC-MS measurement takes approximately 30 minutes per sample and requires high expertise. Data interpretation and validation add at least another 30 minutes per sample, depending on the number of compounds analyzed. However, samples can also be sent to contract laboratories for organic chemical analysis. As explained in response 1.18, S1 Tables 8 and 9, and discussed in Section 3.5 (see text modifications in response 1.18), the sensor-based approach outperforms LC-MS analysis in both cost and staff time required.

**1.30**: Are the proposed regression models robust enough for routine use by utilities without specialist data scientists?

At present, the regression models developed in this study are not robust enough for routine use. Currently, a local calibration is necessary at every measurement site, and the calibration requirements are not fully clear. Further research is needed to clarify calibration requirements and increase model accuracy and robustness. We discuss these implications in our new section 3.5.

Modification in text, lines 517-532:

The sensor-based monitoring approach developed in this study requires local calibration of the regression models. At each monitoring site, sufficient reference samples must be collected and analyzed to achieve satisfactory model performance. The necessity of local calibration was also highlighted in studies using UV absorbance as feedback control parameters for micropollutant abatement in WWTPs, due to differences in wastewater composition [29]. Our study suggests that a minimum of 4 to 13 events, containing an average of 18 samples, may be required for reliable model calibration. Overfitting was an issue for models in catchment M, which used data from only four events, whereas catchment L demonstrated good model performance with data from 13 events. However, more specific calibration requirements remain unclear and likely depend on catchment size and the variability of the substances and rain events monitored [20]. Mutzner et al. [2] reported a median minimum number of seven events for estimating event mean concentrations of organic chemicals in CSOs. Further studies should assess the minimum number of events needed for reliable calibration and investigate how this requirement varies with catchment size, characteristics, and selected chemicals. A practical approach is to apply an iterative cross-validation procedure, where rain events are sampled until the prediction error falls below a predefined threshold [50,51]. In summary, while our modelling approach shows promise, its future application by utilities remains limited due to unclear calibration demands.

Further, we added two new conclusion points, lines 597-606

- The regression models developed in this study require site-specific calibration based on sample collection and chemical analysis. Our analysis suggests that at least 4 to 13 events may be necessary for model calibration, depending on catchment characteristics and chemical variability.
- Sensor-based monitoring has the potential for real-world implementation and for informing mitigation strategies to reduce the environmental impact of CSO discharges. However, further refinements are necessary. Future research should focus on defining calibration requirements, expanding datasets with additional catchments, chemicals, and influence factors, and improving model accuracy. Hybrid approaches that combine sensor monitoring with traditional sampling and LC-MS analysis for selected samples could be explored as they may increase the robustness of chemical predictions.

**Comments on 4. Conclusion**

**1.31**: Why does catchment size impact indoor chemical behavior? Is there a size threshold affecting this?

See response 1.19, which discusses in detail how catchment size impacts the behavior of indoor chemicals.

Modifications in text, lines 571-574:

- Indoor chemicals show a source-specific dilution pattern during wet-weather events. This pattern is more stable in larger catchments (>20’000 inhabitants), whereas indoor chemicals exhibit more pronounced fluctuations due to pulse emissions in smaller catchments. Chemicals from road runoff show source-specific dynamics independent of catchment size.

**1.32**: Why are SAC_254_ nm and NH_4_-N more effective in larger catchments, and what challenges exist in smaller catchments?

SAC_254 nm_ and NH_4_-N are more effective proxies for indoor chemicals in larger catchments, as they reflect the dilution of wastewater by stormwater. Indoor chemicals exhibit clearer dilution patterns in larger catchments. In contrast, indoor chemicals in smaller catchments are more influenced by short-term pulse emissions, resulting in greater variability in chemical concentrations (see comments 1.19 and 1.31). This increased variability complicates the use of general proxies like SAC_254 nm_ and NH_4_-N in smaller catchments.

Modifications in text, lines 575-576:

- Level, flow, EC, SAC_254 nm_, and NH_4_-N are suitable sensor proxies for polar indoor chemicals in larger catchments (M and L), where these chemicals show a pronounced dilution pattern.

**1.33**: What other environmental factors could improve the prediction of road-runoff chemicals?

Several environmental factors could potentially improve predictions of road-runoff chemicals originating from tire wear particles. These include the antecedent dry period, rainfall intensity, and traffic volume. However, findings in the literature on the influence of these environmental factors are mixed. For example, different studies did not find a significant correlation between the length of the antecedent dry period and the concentrations of road-runoff chemicals [3,12]. Regarding rainfall intensity, Liu et al. [13] observed a negative correlation with rubber-derived chemicals, where peak concentrations occurred later during rainfall events. In contrast, our study found that road-runoff chemicals consistently peaked at the beginning of rainfall events, indicating different dynamics under our conditions. Traffic volume and speed are other potentially influential factors [14], but reliable data on traffic patterns is oftentimes not available. Overall, significant uncertainties remain regarding the transport and leaching mechanisms of tire-wear particles [15]. As the focus of our study was on sensor proxies, further research is needed to better understand the processes governing the release of these chemicals and to identify which environmental factors can reliably serve as proxies for predicting road-runoff pollution.

Modification in text, lines 410-416:

Both SLR and MLR models exhibit broad residual distributions, indicating limited predictive accuracy with the current set of sensor parameters (S1 Figs. 19 and 20). Hence, additional proxies need to be identified to account for other influencing factors. Current studies report mixed findings on the influence of other environmental factors, such as antecedent dry period, rainfall intensity, and traffic volume and speed, on chemicals from road runoff [32,46–48]. This underscores the need for further research to clarify the transport and leaching mechanisms of these substances and to better understand how these factors are interrelated.

Lines 588-592:

- No universally valid sensor proxy has been identified for polar organic chemicals from road runoff. Water flow and level have been identified as the most effective proxies, yielding median prediction errors of 41–44%. However, the role of environmental factors that potentially affect chemical leaching, such as antecedent dry period, rain intensity, or traffic volume, is currently unclear or limited by data availability [32,46,47,54]. Further research is needed to identify environmental factors or sensor proxies that better reflect the complex release mechanisms of these chemicals, given their ecotoxicological relevance [55,56].

**1.34**: Why do additional sensors not significantly improve predictions? Is it due to sensor limitations or chemical complexity?

Additional sensors often fail to improve predictions because their signals do not capture the actual patterns of specific organic chemicals. Instead, they capture noise, leading to overfitting. The main issue lies in the complex behavior of organic chemicals in sewer systems, which is challenging to represent with generic sensor signals. Thus, improving predictions requires proxies that more directly reflect the underlying chemical processes.

Modifications in text, lines 593-596:

- Adding more sensors as predictors generally does not improve prediction performance significantly (change in prediction error < 10%), because they often capture noise rather than actual chemical patterns. Thus, online measurements with a single sensor parameter (level, flow, SAC_254 nm_, NH_4_-N) are promising for the monitoring of indoor and road runoff chemicals.

**1.35**: How can future studies capture complex chemical behaviors like those of PPPs and biocides?

To date, no suitable sensor-based proxy has been identified that reliably reflects the behavior of PPPs and biocides in sewer systems. Due to the absence of a source-specific behavior, finding a proxy likely remains unfeasible in the foreseeable future. Therefore, future studies will still need to rely on the traditional approach of grab sampling combined with LC-MS analysis for these substances.

Modifications in text, lines 588-592:

- No suitable sensor proxies could be identified for PPPs, biocides, and chemicals originating from many diverse sources due to their non-source-specific nature and the lack of sensor proxies reflecting their highly variable concentrations. Given the potential toxicity of these substances, their monitoring is critical, but must continue to rely on traditional monitoring methods for the foreseeable future.

**1.36**: Could hybrid methods combining sensors and traditional monitoring improve accuracy for complex chemicals?

We believe that hybrid approaches, which combine sensor monitoring with traditional sampling and LC-MS analysis, have the potential to improve chemical predictions. For example, periodic (e.g., monthly) recalibration could help adjust sensor-based models to capture the highly variable behavior of organic chemicals in CSOs. However, the optimal frequency, number of samples, and implementation of such hybrid methods still need to be explored in future research. Hence, we added this idea as an outlook in the conclusion section.

Modifications in text, lines 598-6004:

- Sensor-based monitoring has the potential for real-world implementation and for informing mitigation strategies to reduce the environmental impact of CSO discharges. However, further refinements are necessary. Future research should focus on defining calibration requirements, expanding datasets with additional catchments, chemicals, and influence factors, and improving model accuracy. Hybrid approaches that combine sensor monitoring with traditional sampling and LC-MS analysis for selected samples could be explored as they may increase the robustness of chemical predictions.

**Comments on References**

**1.37**: Some references use different citation styles (e.g., references 3 and 18 don’t follow the journal article format properly).

Thank you for checking the references so carefully. We revised them and corrected the mistakes.

Modifications:

6. Manasa RL, Mehta A. Wastewater: Sources of pollutants and its remediation. In: Gothandam KM, Ranjan S, Dasgupta N, Lichtfouse E, editors. Environmental Biotechnology Vol 2. Cham: Springer International Publishing; 2020. p. 197–219. doi:10.1007/978-3-030-38196-7.

25. Mohan L, Leena A, Mithun S, Sathriyan V, Nachiappan S. Integrated sensor-based system for remote monitoring of sewage water quality and environmental conditions. AIP Conf Proc. 2024 Nov 25;3192(1):20033. doi:10.1063/5.0242458.

**1.38**: "Manasa RL, Mehta A. Environmental Biotechnology Vol. 2" is listed as a book chapter, but the journal article format isn't followed for the citation.

We corrected this reference (see response 1.37).

**1.39**: Some references lack proper DOI formatting (e.g., references 26 and 27).

Reference 38 (previously 26) is a report from a governmental institution of Austria and does not have a doi number. We corrected the reference and added the url. However, we checked the doi of reference 31 (previously 27) and believe that “doi:10.1021/acs.est.0c00872” is the correct format.

36. Zessner M, Schaar H, Zoboli O, Kreuzinger N, Kittlaus S, Muschalla D, et al. Zukünftige stoffliche und mikrobiologische Herausforderungen für die kommunale Siedlungswasserwirtschaft: SIWAWI-Endbericht [Internet]. Bundesministerium Land- und Forstwirtschaft, Regionen und Wasserwirtschaft. Wien; 2022. Available from https://www.bmluk.gv.at/service/publikationen/wasser/zukuenftige-stoffliche-und-mikrobiologische-herausforderungen-fuer-die-kommunale-siedlungswasserwirtschaft.html.

**1.40**: References 23 and 24 appear to be from the same author (Launay et al.) but are slightly different in formatting and may be a duplicate of the same research.

You correctly noted that references 1 and 31 are from the same authors. However, these are two different studies. Reference 1 characterized organic micropollutants in CSOs and was published in Water Research, while reference 23 is a conference paper about organic micropollutants in a receiving water body.

**1.41**: Reference 25 mentions a preprint ("OSF Prepr"), which should clearly indicate that it’s a preprint with proper notation.

Meanwhile, reference 25 has been published in Scientific Data from Nature, and is now referenced correctly.

33. Lechevallier P, Gruber G, Bares V, Neuenhofer N, Waldner L, Mahajan A, et al. Dataset on wastewater quality monitoring with adsorption and reflectance spectrometry in the UV-vis range. Sci Data. 2025;12:1296. doi:10.1038/s41597-025-05459-x.

**1.42**: There are inconsistencies in punctuation, such as missing periods at the end of some references (e.g., reference 16).

Thanks for pointing out, we have now removed all inconsistencies in punctuation.

## Reviewer 2: Kang Mao

This paper is the first to systematically explore the parameters of online sensors as alternative indicators for the dynamic changes of organic chemical substances in the combined sewer network during rainy days. I think the topic selection has certain technological innovation value in environmental monitoring. Through the data analysis of multi-scale catching-up areas (with 2,000 to 200,000 residents), the dynamic characteristics of chemical substances from different sources were revealed, and a prediction model framework based on sensor parameters was proposed, providing a feasible solution for reducing CSO pollution. The research design is reasonable, the data analysis method is rigorous, and the conclusion has direct reference significance for the decision-making of urban drainage management. Some important issues need to be considered and some problems to be further improved if the author try to submit to other appropriate journals:

Thank you for your detailed and encouraging assessment of our manuscript. We appreciate your acknowledgement of the study’s environmental significance. In response to your comments, we have carefully reviewed the manuscript and made several clarifications to address the issues you pointed out, including:

- Improvements of title and keywords
- Clarification of ambiguities in the abstract, introduction, and method sections
- New section 3.5 addressing practical considerations and further research needs for real-world implementation
- Additional figures and tables in supplementary files

These changes are now reflected in the revised manuscript (see the manuscript with tracked changes and answers below, line numbers refer to the clean manuscript). Except for a few comments that were not in the scope of our work, we implemented most of your suggestions in the manuscript and feel they substantially improve its quality.

**Comments on title**

**2.1**: The title of this article has a clear research theme, highlighting the research background under the specific condition of the rainy season and also reflecting its research purpose. However, the title expression lacks novelty and fails to highlight the uniqueness or innovation points of the research. It is suggested to adopt the dual-element structure of "methodology - innovation point". Meanwhile, the title information is not rich enough.

Thank you for your suggestion of adding the innovation aspect to the title.

We changed it to:

Exploring online sensors as proxies for polar organic chemicals - An innovative approach for combined sewer overflow monitoring

**Comments on Abstract**

**2.2**: Although this abstract mentions that the research method is to analyze the data of the three catchments, details such as the specific data collection methods and analysis methods can be appropriately mentioned to enable readers to have a clearer understanding of the research methods. The abstract does not mention the possible limitations of the research, such as the representativeness of the data and the applicable scope of the model. Moreover, appropriately mentioning the limitations in the abstract can enable readers to have a more comprehensive understanding of the reliability of the research and the potential directions for improvement.

Thank you for your suggestion. We modified the abstract so that it now contains more details on data collection, analysis methods, and limitations of the study.

Modifications in text, lines 28-30:

We therefore used sensor and organic chemical data collected in three urban catchments of varying sizes. Correlations between chemicals from the same source and sensor parameters were calculated.

Lines 32-34:

Additionally, linear regressions were developed to predict organic chemical concentrations from sensor data. Models based on SAC_254 nm_ and NH_4_-N predict indoor chemical concentrations with median relative errors of 32% and 29%, respectively, in the large catchment.

Lines 39-42:

However, for real-world implementation, further research is needed to refine calibration re-quirements and validate the models across diverse catchments. Nevertheless, these models are promising for cost-effective, long-term monitoring of organic chemicals and for mitigating the impact of CSO discharges.

**2.3**: Line 22-23: “Currently, most overflow sites are not monitored because traditional methods are costly and time-consuming.”What are the traditional detection methods and their shortcomings? Cite the literature that supports the shortcomings of traditional detection methods.

Traditionally, organic chemicals are analyzed by taking grab samples followed by laboratory LC-MS analysis. We added these details to the abstract as discussed in response 1.1 (see text modification there).

Additionally, we added further sources discussing the shortcomings of grab sampling and LC-MS analysis in the introduction.

Modifications in text, lines 64-65:

However, this approach is unsuitable for continuous monitoring because it is cost- and time-intensive, requires automated sampling, and provides only discrete time points [16-19].

**Comments on 1. Introduction**

**2.4**: Line 44-46: “These discharges of a mixture of wastewater and stormwater contain numerous organic chemicals [1,2] that threaten aquatic species.”How was the conclusion that aquatic species are threatened reached? Supplement the toxicological evidence of the threat to aquatic organisms. There is a lack of specific references.

Thank you for noting that the sources were missing for the ecotoxicological relevance of CSOs. We have now added three studies that examine the ecotoxicological risk of CSO discharges.

Modifications in text, lines 46-48:

These discharges of a mixture of wastewater and stormwater contain numerous organic chemi-cals [1,2] that threaten aquatic species [3–5].

**2.5**: Line 57-59: “For example, a sampling interval of 3 minutes has been recommended because organic chemicals from municipal wastewater, also termed indoor chemicals, exhibit very high concentration fluctuations, particularly in small catchments (2,700 inhabitants) [10].”Why is it recommended that the sampling time be three minutes instead of one minute or thirty seconds? Clarify the statistical basis of the 3-minute sampling interval.

Furrer et al. [1] compared 3-minute grab samples to 3-minute composite samples, to estimate whether the concentration dynamics of organic chemicals in CSOs are below 3 minutes. When the difference between the two sampling methods is smaller than the uncertainty of chemical analysis, they assume that the real dynamics do not exceed 3 minutes [1]. Based on visual assessment of the time series and comparison with the differences between grab and composite samples, they defined a threshold of 70% [1]. I.e., a difference between grab and composite samples larger than 70% suggests that a substance exhibits concentration dynamics below three minutes [1]. As they found that some indoor chemicals exceed this 3 min interval, they recommend a sampling interval of 2 minutes [1]. Also Ort [8] recommends a sampling interval of 2 minutes, depending on the variability of a substance. Based on these expert recommendations, we modified the text and specified a 2-minute interval as necessary to capture short-term concentration fluctuations. We cited both studies, allowing interested readers to refer to them for further methodological details.

Modifications in text, lines 59-62:

A sampling interval of 2 minutes is recommended to capture the actual dynamics of organic chemicals in CSOs [13,16]. Particularly, organic chemicals from municipal wastewater, also termed indoor chemicals, exhibit very high concentration fluctuations in small catchments due to pulsed wastewater flushes from households [13,16].

**Comments on 2. Material and methods:**

**2.6**: Line 108-109: “For rain event M.2, sampling started 50 minutes late due to problems with the autosampler.”Will sampling delay lead to missing data or underrepresentation? It is suggested to supplement and explain: "Cubic spline interpolation was adopted to fill the data gap of the first 50 minutes. The K-S test proved that there was no significant difference between the interpolated data and the measured value distribution (p>0.05)."

Interpolation was not possible for the first 50 minutes of rain event M.2 because no data existed before the gap to anchor a spline fit. Moreover, the initial phase of a rain event is highly dynamic, often characterized by first flush effects, and rapidly increasing chemical concentrations. In the case of M.2, the increasing part of the peak was missed, hence backward interpolation could falsely suggest decreasing concentrations of outdoor chemicals or increasing concentrations of indoor chemicals throughout the rain event. Also, the definition of an anchor and tipping point characterizing in-/decreasing concentrations at the beginning of a rain event is highly speculative. Therefore, using interpolation would not reliably reconstruct the missing data, which is unavoidably missed. This data cutoff likely weakens correlations and predictions for rain event M.2. The implications of this data cutoff are discussed in more detail in response 1.7 and section 3.4 (see text modification there).

**2.7**: Line 157-158: “Programming language. Python (version 3.9.18, Python Core Team, 2023) was used for data pretreatment and analysis.”Why choose Python instead of other Programming languages? Supplement the basis for Python language selection (such as the machine learning advantages of the scikit-learn library and the specificity of the Pandas library in time series processing)

Python was chosen because it is an open-source, freely available language widely used in scientific research and data science. It offers powerful libraries well-suited for our analysis: Pandas provides efficient handling and processing of time-series and tabular data, NumPy enables fast numerical operations, and scikit-learn includes robust and well-tested implementations for linear regression models. These reasons make Python highly effective for modeling environmental data.

Modifications in text, lines 186-188:

Python (version 3.9.18, Python Core Team, 2023) was used for data preprocessing and analysis, due to its widespread use in scientific research and its robust libraries for data processing and modeling.

**Comments on highlights:**

**2.8**: First of all, there is a lack of keywords related to the research results: The article has drawn relevant conclusions about the correlation between different types of organic chemicals and sensor parameters, as well as the prediction model through research. However, contents related to the research results, such as "correlation" and "prediction model," are not reflected in the keywords. It may make it difficult for readers to directly obtain the core research results information of the article when searching. Secondly, some keywords can be further refined: For example, "urban drainage" is relatively broad. If it could be refined to "combined sewer system drainage", it might more accurately reflect the specific context of the research.

Thank you for this input. We deleted broad terms and added more specific terms related to our research.

Modifications of keywords:

combined sewer overflows; stormwater; organic micropollutants; pharmaceuticals; personal care products; tire wear leachates; pesticides; biocides; plant protection products; automated sampling, LC-MS; sensors; wastewater characteristics; proxies, correlation; linear regression models

**Other comments**

**2.9**: The scope of application and verification of the model need to be further clarified. The article emphasizes that the applicability of the model should be based on the premise of similar characteristics to the catchment area in this study, but does not specifically define the dimension of "feature similarity" (such as the topological structure of the pipe network, land use type, rainfall intensity, etc.).

Suggestion: Firstly, a sensitivity analysis is supplemented to explore the influence of factors such as the scale of the catchment area, population density, and rainfall patterns on the prediction error of the model. Secondly, add the test results of independent validation datasets (such as catchment areas in other cities), or evaluate the generalization ability of the model through cross-validation.

Thank you for this valuable comment. We have removed the sentence in the abstract suggesting that the catchment area must be similar, as our data do not support this conclusion. As our dataset includes three catchments with differing characteristics, it does not allow us to assess the transferability of our models to other catchments with similar characteristics. Instead, we highlight the actual limitations of the models in their current state of development in sections 3.4 limitations and 3.5 practical considerations and research needs (new section), such as the need for local calibration. See response 2.2 for modifications in the abstract and response 1.30 in results & discussion.

Regarding the first suggestion: a sensitivity analysis exploring the influence of catchment size, population density, or rainfall patterns is not feasible because these variables are not included as predictors in our models. Instead, we calibrated local models for each catchment. Due to the different wastewater characteristics in each catchment/sample site, it is not possible to develop a global model to assess these influencing factors. Wittmer et al. [16] developed a model predicting micropollutant abatement in wastewater treatment plants based on SAC_254 nm,_ which also requires recalibration in every catchment. Calibration requirements are now discussed in our new section 3.5, see response 1.30.

Concerning the second suggestion: unfortunately, no independent validation datasets from other catchments are available to us, combining sensor and organic chemical data. In general, high-resolution chemical datasets are very scarce as they require months to years of sampling and analysis, which is very costly. If available, these datasets usually do not include sensor measurements. However, we did apply cross-validation to evaluate model performance across different rain events within each catchment, which provides an initial assessment of model robustness. Cross-validation also allowed us to identify overfitting in catchment M, see text modifications in response 1.20.

**2.10**: The reasons for the failure of prediction of pesticide chemical substances need to be explored in depth. The failure to predict pesticide concentrations was attributed to "diverse spatiotemporal patterns", but specific driving factors (such as the application cycles of different pesticides, differences in surface retention time, etc.) were not analyzed.

Suggestion: Firstly, supplement the source analysis of pesticides (such as agriculture vs. application in urban green spaces), and explore the internal mechanism of their low correlation with sensor parameters. Secondly, it is possible to attempt to introduce time lag variables or meteorological data (such as the number of drought days before rainfall) as supplementary parameters of the model.

Regarding your first suggestion: The sources of pesticides in our study are provided in S1 Table 3, as noted in lines 156-157. This table specifies whether each compound is used as a biocide, a plant protection product (PPP), or both. S1 Fig. 6 further differentiates their Pearson correlation coefficients (PCCs). However, because the PCCs are generally low for both biocides and PPPs (indicating no source-specific temporal dynamics) and because some substances are used as both, biocides and PPPs, we analyzed them as one combined group. Similar to other studies, we observed diverse, fluctuating pesticide and biocide concentrations [9]. Also, Furrer et al. [3] did not observe a source-specific behavior when analyzing a larger set of 16 pesticides in CSOs. When chemicals of a substance group exhibit diverse behavior (lack of source-specificity), no universal proxy can be identified for the entire group. Therefore, exploring additional proxies, such as specific use patterns or mobilization mechanisms, is unlikely to yield meaningful improvements on our dataset. Additionally, our study focused on sensor proxies and urban catchments where agricultural use is minimal. Unfortunately, data on pesticide applications in urban green spaces, private gardens, and households are generally not available and would need to be collected for each catchment through surveys. For example, Wittmer et al. [17] modeled facade leaching of biocides, which requires detailed data on applied quantities, substance-specific degradation rates, and the amount of rainfall turned into runoff. This data is unavailable for our catchments. Further research is needed to study the behavior of more than six pesticides in CSOs and clarify whether subgroups with similar/source-specific behavior can be identified. If such patterns can be identified, additional influencing parameters can be explored. The lack of source-specific behavior, due to the complexity of pesticide mobilization, is discussed in lines 269-277 (section 3.1) and 425-430 (section 3.3) of the manuscript and response 1.23 (see text modifications there). We additionally modified section 3.2 to improve our explanations on the implications of the lack of source-specific behavior on sensor proxies.

Modification of text, lines 334-339:

No sensor parameter proxy can be identified for this chemical group because their diverse, fluctuating concentrations are not reflected by any sensor parameter available (S1 Fig. 11). Given these chemicals’ lack of source-specific behavior [32], finding a sensor proxy for this chemical group may remain infeasible. For example, Carpenter et al. [31] developed separate models with different predictors for every pesticide studied in surface water.

Regarding your second suggestion: As explained above, due to the lack of source-specificity of the selected chemicals, a single parameter will not serve as a proxy for the entire group. We tested correlations between time-lagged water level and flow, but these were weak (S1 Fig. 14). Furthermore, meteorological variables like antecedent dry periods are unlikely to enhance predictions for biocides and PPPs in our urban context. Previous studies have shown that some of these substances occur even during dry weather, suggesting indoor uses [9,18], and Carpenter et al. [19] found that antecedent dry periods were not significant predictors for most pesticides.

Due to these complexities and data limitations, we were unable to explore the specific mechanisms or additional predictors necessary to improve pesticide modeling in our study. Instead, we focused on measurable sensor proxies. Further research, including more chemicals and targeted data collection on pesticide use and fate processes, is necessary to address these challenges.

**2.11**: The details of the method need to be refined to ensure repeatability. The measurement frequency of sensor parameters (such as SAC at 254 nm) and the chemical substance sampling interval are not clearly specified, which may affect the reliability of temporal correlation analysis. The minimum sample size required for traditional sampling data during the model calibration phase (such as event count, time coverage) is not mentioned.

Suggestion: First, supplement the technical details of sensor and sampling synchronization (such as time alignment methods and data interpolation strategies) in the Methods chapter. Secondly, clarify the data requirements for the calibration stage (such as at least how many rainfall events or the range of flow variation should be covered).

Regarding your first suggestion: The method section “Sampling procedure” and S1 Table 2 specify the sampling interval for organic chemicals, while S1 Table 7 details the measurement intervals for sensor parameters. To improve clarity, we strengthen the reference to S1 Table 7 in the manuscript (see text modification in response 1.16). Additionally, we included further details on sensor and sampling synchronization, explaining that linear interpolation was used to align sensor measurements with chemical sampling times (see text modification in response 1.18).

Regarding your second suggestion: The regression models developed in this study require site-specific calibration based on chemical sampling and analysis. Our data indicate that at least 4-13 rain events may be necessary for reliable model calibration, depending on catchment size and chemical variability. However, further studies are needed to more precisely define the minimum data requirements for robust model calibration across different settings (see explanation and text modification in response 1.30).

**2.12**: The practical application path needs to be specified. The conclusion states that "sensor monitoring can be implemented after passing the calibration stage", but it does not discuss the balance between calibration costs (such as traditional sampling frequency and analysis fees) and benefits.

Suggestion: First, add economic analysis to compare the long-term monitoring cost of sensors with the full-cycle cost of traditional methods. Secondly, specific strategies for optimization in the calibration stage are proposed (such as the selection of key parameters and the optimization of the sampling time window).

Regarding your first suggestion: we deleted the sentence “sensor monitoring can be implemented after passing the initial calibration stage” in the abstract, as we noticed that this conclusion cannot be inferred from our study. Instead, we added the limitations of our model in the abstract, see response 2.2.

Additionally, we added a new section 3.5, which discusses the cost of sensor-based monitoring with the traditional approach (grab sampling + LC-MS). More specifically, S1 Tables 8 and 9 compare the costs of both methods. The responses to comments 1.18, 1.27, and 1.29, and section 3.5 discuss costs in detail and show that the sensor-based approach is less costly than traditional monitoring based on automated sampling and LC-MS analysis in the long term (see text modifications there).

Regarding your second suggestion: Based on our limited dataset, which covers three catchments with different characteristics, we are unable to define universally valid strategies for model calibration. As discussed in detail in responses to comments 1.30 and 2.11, local calibration based on high-resolution (2 min) chemical sampling is required for every catchment due to variable wastewater characteristics. The minimum number of samples for calibration likely depends on catchment size and chemical variability. One possible approach to defining the local requirements for calibration is iterative cross-validation. More and more rain events are sampled and used for model training until the prediction error falls below a predefined threshold. We discuss the limitations of our study regarding calibration requirements and our suggestions for future model development in section 3.5 and responses 1.30 and 2.11 (see text modifications there).

**2.13**: Chart optimization. Supplement the correlation coefficient matrix diagrams of different chemical substance categories and sensor parameters to visually display the key correlations. In the result section, add a scatter plot of the model's predicted values and measured values, supplemented by an error distribution histogram.

Thanks for your suggestions. Unfortunately, we do not understand your first suggestion on supplementing the correlation coefficient matrix diagram. Figure 2 already displays the correlations between each chemical group and sensor parameter. Strong correlations are highlighted by darker colors, as indicated by the colorbar on the right-hand side.

Regarding your second suggestion: We agree that visualizing predicted vs. measured values and the associated residuals adds valuable information. We have therefore added new figures (S1 Figs. 19 and 20) to S1 File, showing scatter plots of normalized measured vs. predicted concentrations for each chemical group and catchment, along with histograms of the residual distributions. These plots are referenced multiple times in section 3.3. Due to their complexity and size, we chose to include them in the supplementary S1 File rather than the main text. In addition, Fig. 3 in the manuscript summarizes model performance using RRMSE between measured and predicted concentrations, providing a concise and informative overview.

Modifications in text, lines 360-366:

Hence, the model’s predictive accuracy improves with increasing catchment size (Fig. 3c, S1 Fig. 19) as dilution patterns become more stable (S1 Figs. 3-5). In the small catchment S, the model fits, and therefore, the predictions are only good for selected indoor chemicals (acesulfame, caffeine, cyclamate, citalopram, and hydrochlorothiazide) (R^2^ > 0.5, S3 Table 15) that show a dilution pattern and correlate well with sensor proxies (S1 Figs. 3 and 11). In catchment M, model fits are generally poor (median R^2^ = 0.37, Fig. 3a, S1 Fig. 19) as correlations with level are weak (PCC = -0.23, Fig. 2a).

Lines 410-412:

Both SLR and MLR models exhibit broad residual distributions, indicating limited predictive accuracy with the current set of sensor parameters (S1 Figs. 19 and 20).

Lines 417-421:

SLR models fail to predict PPP and biocide concentrations. Flow, temperature, and SAC_254 nm_ are selected as predictors in catchments S, M, and L, respectively (Table 4). However, models based on these sensors result in poor model fits (median R^2^ = 0.20, 0.11, and 0.15 in catchments S, M, and L, Fig. 3a), as they do not capture the PPP and biocide dynamics (S1 Fig. 19).

Lines 425-428:

The poor model performance can be attributed to the lack of source-specific behavior of this chemical group and the fact that the used sensor proxies do not accurately reflect the strongly fluctuating and variable concentration patterns of any of the PPPs and biocides studied (S1 Figs. 11, 19, and 20).

**2.14**: Supplement Table: Table 1: Minimum Data Requirement Matrix in Calibration Stage (Number of Rainfall Events × Flow Variation × Types of Pollutants)

We agree that understanding the minimum calibration requirements is essential for practical implementation. However, as explained in detail in our responses to comments 1.30 and 2.11, the limited size and scope of our dataset do not allow us to define such requirements systematically. Calibration requirements likely vary depending on pollutant type and catchment characteristics. Our study aimed primarily to explore the feasibility of correlating sensor measurements with organic chemical concentrations in CSOs, which is a relatively novel approach. Further studies with more extensive datasets across multiple sites are needed to define robust calibration thresholds and further steps towards real-world implementation. The practical limitations of our approach, including calibration demands and needs for further research, are now addressed in the new section 3.5, see text modifications in response 1.30.

**2.15**: Discussion extension. To explore the potential impact of sensor maintenance (such as biofilm interference and drift correction) on the long-term stability of the model. Compare the results of this study with the application differences of SAC_254nm_ as a substitute parameter for micro-pollutants in sewage treatment plants.

Regarding your first point: With functioning automated sensor cleaning and weekly manual maintenance, Lechevallier et al. [2] effectively prevented sensor drift and biofilm formation during our study. However, further investigations are needed to assess potential impacts on model stability under conditions without automated cleaning systems or with less frequent maintenance. We added these concerns to the new section 3.5. These text modifications are already shown in response 1.27, but are repeatedly shown here as the context of these reviewer comments differs.

Modification in text, lines 536-545:

Frequent maintenance, including sensor cleaning and recalibration, is therefore essential to prevent sensor drift and biofilm formation, thereby ensuring the long-term stability of data and model performance [33]. The quality of the data used in this study was ensured through regular sensor maintenance (see S1 Table 7), which requires trained staff. For example, flow sensors were cleaned one to four times per year, while UV-Vis probes were cleaned on a weekly or bi-weekly basis (S1 Table 7). Where feasible, automated sensor cleaning with compressed air was used to further enhance sensor performance [33]. Lechevallier et al. [33] also demonstrated the value of automated quality control, including outlier detection, drift analysis, and weekly data inspection. However, further research is needed to evaluate the impact of reduced maintenance on sensor performance and model stability over longer periods.

Regarding your second point: Thanks for the suggestion to compare our results with studies using SAC in WWTPs. We compared the requirements of model calibration, the model error, and measurement conditions to these studies and discussed these facts in the new section 3.5. These text modifications are already shown in responses 1.26 and 1.30, but are repeatedly shown here as the context of these reviewer comments differs.

Modifications in text, lines 506-513:

For context, RRMSEs of 10% to 20% are typically considered satisfactory for UV-Vis measurements in urban drainage systems [49], and a 10% error is common for chemical sampling and analysis [13]. In contrast, Hubeaux et al. [27] demonstrated that micropollutant abatement in wastewater treatment plants can be predicted with errors as low as 2% based on SAC_254 nm_ measurements. However, their study relied on 24-hour composite samples and did not address the short-term fluctuations typical of sewer systems. While very high precision may be achievable in relatively stable WWTP conditions, higher error margins need to be accepted in sewer monitoring due to greater variations in the concentrations of organic chemicals.

Modification in text, lines 517-521:

The sensor-based monitoring approach developed in this study requires local calibration of the regression models. At each monitoring site, sufficient reference samples must be collected and analyzed to achieve satisfactory model performance. The necessity of local calibration was also highlighted in studies using UV absorbance as feedback control parameters for micropollutant abatement in WWTPs, due to differences in wastewater composition [29].

## Additional changes

- We clarified the goal, scope, and limitations of our study throughout the manuscript. These revisions emphasize that this work represents an initial exploration of using sensor proxies for polar organic chemicals in CSOs and highlight the need for further research to support real-world implementation.
- We improved figure layouts, e.g., grid lines, more saturated colors.
- We checked that we comply with PLOS ONE’s style requirements (e.g., Fig. instead of Figures, table legends below tables, corrections of mistakes in references).
- We specified in the Methods section that we did not require a permit for this work because the data collected from three different field sites are published on public repositories.

Modification in text, lines 99-101:

Data for this study were collected by Furrer et al. [13,32] and Lechevallier et al. [33] and published as openly accessible datasets in the Eawag Research Data Institutional collection [34,35]. As the data were already published and the authors conducted no new sampling, this study did not require any permits.

- We added the following statement in the cover letter:

The funders had no role in study design, data collection and analysis, decision to publish, or preparation of the manuscript.

- We removed S1 Figs. 1-3 because we could not fully clarify whether they comply with the CC-BY licence. Instead, in the main text, we refer to the publications from which they were copied.

## References

1.	Furrer V, Mutzner L, Ort C, Singer H. Micropollutant concentration fluctuations in combined sewer overflows require short sampling intervals. Water Res X. 2023;21(July):100202. doi:10.1016/j.wroa.2023.100202.

2.	Lechevallier P, Gruber G, Bares V, Neuenhofer N, Waldner L, Mahajan A, et al. Dataset on wastewater quality monitoring with adsorption and reflectance spectrometry in the UV-vis range. Sci Data. 2025;12:1296. doi:10.1038/s41597-025-05459-x.

3.	Furrer V, Froemelt A, Singer H, Ort C. Source-specific dynamics of organic micropollutants in combined sewer overflows. Water Res. 2025;123416. doi:10.1016/j.watres.2025.123416.

4.	Zessner M, Schaar H, Zoboli O, Kreuzinger N, Kittlaus S, Muschalla D, et al. Zukünftige stoffliche und mikrobiologische Herausforderungen für die kommunale Siedlungswasserwirtschaft: SIWAWI-Endbericht [Internet]. Bundesministerium Land- und Forstwirtschaft, Regionen und Wasserwirtschaft. Wien; 2022.

5.	Furrer V, Junghans M, Singer H, Ort C. Realistic exposure scenarios in combined sewer overflows: how temporal resolution and selection of micropollutants impact risk assessment. Water Res. 2025;278(February):123318. doi:10.1016/j.watres.2025.123318.

6.	Furrer V, Froemelt A, Singer H, Ort C. Data for: Source-specific dynamics of organic micropollutants in combined sewer overflows (Version 1.0). 2024. doi:10.25678/000DNY.

7.	Lechevallier P, Gruber G, Bares V, Neuenhofer N, Waldner L, Mahajan A, et al. Dataset on wastewater quality monitoring with adsorption and reflectance spectroscopy in the UV/Vis range. 2024. doi:10.25678/000D3C.

8.	Ort C. Quality assurance/quality control in wastewater sampling. In: Quality assurance & quality control of environmental field samples. 2014. p. 146–68. doi:10.4155/EBO.13.476.

9.	Wittmer IK, Bader HP, Scheidegger R, Singer H, Lück A, Hanke I, et al. Significance of urban and agricultural land use for biocide and pesticide dynamics in surface waters. Water Res. 2010;44(9):2850–62. doi:10.1016/j.watres.2010.01.030.

10.	Burkhardt M, Zuleeg S, Vonbank R, Schmid P, Hean S, Lamani X, et al. Leaching of additives from construction materials to urban storm water runoff. Water Sci Technol. 2011;63(9):1974–82. doi:10.2166/wst.2011.128.

11.	Brito RS, Pinheiro HM, Ferreira F, Matos JS, Lourenço ND. In situ UV-Vis spectroscopy to estimate COD and TSS in wastewater drainage systems [Internet]. Vol. 11, Urban Water Journal. Taylor & Francis; 2014. p. 261–73. doi:10.1080/1573062X.2013.783087.

12.	Challis JK, Popick H, Prajapati S, Harder P, Giesy JP, McPhedran K, et al. Occurrences of tire rubber-derived contaminants in cold-climate urban runoff. Environ Sci Technol Lett. 2021;8(11):961–7. doi:10.1021/acs.estlett.1c00682.

13.	Liu YH, Mei YX, Liang XN, Ge ZY, Huang Z, Zhang HY, et al. Small-intensity rainfall triggers greater contamination of rubber-derived chemicals in road stormwater runoff from various functional areas in megalopolis cities. Environ Sci Technol. 2024;58(29):13056–64. doi:10.1021/acs.est.3c10737.

14.	Shin H, Hyun M, Jeong S, Ryu H, Lee MG, Chung W, et al. A correlation study of road dust pollutants, tire wear particles, air quality, and traffic conditions in the Seoul (South Korea). Atmos Pollut Res. 2024;15(12):102309. doi:10.1016/j.apr.2024.102309.

15.	Wagner S, Hüffer T, Klöckner P, Wehrhahn M, Hofmann T, Reemtsma T. Tire wear particles in the aquatic environment - A review on generation, analysis, occurrence, fate and effects. Water Res. 2018;139:83–100. doi:10.1016/j.watres.2018.03.051.

16.	Wittmer A, Heisele A, McArdell CS, Böhler M, Longree P, Siegrist H. Decreased UV absorbance as an indicator of micropollutant removal efficiency in wastewater treated with ozone. Water Sci Technol. 2015;71(7):980–5. doi:10.2166/wst.2015.053.

17.	Wittmer IK, Scheidegger R, Stamm C, Gujer W, Bader HP. Modelling biocide leaching from facades. Water Res. 2011;45(11):3453–60. doi:10.1016/j.watres.2011.04.003.

18.	Bollmann UE, Tang C, Eriksson E, Jönsson K, Vollertsen J, Bester K. Biocides in urban wastewater treatment plant influent at dry and wet weather: Concentrations, mass flows and possible sources. Water Res. 2014;60:64–74. doi:10.1016/j.watres.2014.04.014.

19.	Carpenter CMG, Wong LYJ, Gutema DL, Helbling DE. Fall creek monitoring station: Using environmental covariates to predict micropollutant dynamics and peak events in surface water systems. Environ Sci Technol. 2019;53(15):8599–610. doi:10.1021/acs.est.9b02665.
